# Supplementary material for: Global landscape of SARS-CoV-2 genomic surveillance, public availability extent of genomic data, and epidemic shaped by variants
Source: Res Sq. 2021 Sep 29:rs.3.rs-927070. Preprint. [Version 1] doi: 10.21203/rs.3.rs-927070/v1 (PMC8491853; doi:10.21203/rs.3.rs-927070/v1)
Supplement: Supplement 2 [file 2d96475c1883cbe2b9d975e5.pdf]

We thank all the authors contributed to generating and sharing sequences to GISAID, GenBank, National Genomics Data Center, National Microbiology Data Center, and China National GeneBank

1. ViroGenetics - BSL3 Laboratory of Virology, Maopolska Centre of Biotechnology, Jagiellonian University;

1270 Natividad Road Salinas, CA 93906

African Centre for Excellence for Genomics of Infectious Diseases (ACEGID), Redeemers University

Alaska State Virology Laboratory

All India Institute of Medical Sciences, Bhopal

AMES Centro Polidiagnostico Strumentale S.r.l.

Andersen lab at Scripps Research

Arizona State Public Health Laboratory

Arizona State University

AZ SINT-JAN BRUGGE

Bergthaler laboratory, CeMM Research Center for Molecular Medicine of the Austrian Academy of Sciences

Biocruces Bizkaia

Biolab Diagnostic Laboratories

Bioptick laborato, s.r.o.

Bundeswehr Institute of Microbiology

CBRN Defence and Security, Swedish Defence Research Agency

CDC Division of Viral Diseases, Pathogen Discovery

Center for Global Health, University of New Mexico Health Sciences Center

Center for Laboratory Medicine

Central Public Health Laboratory, National Public Health Organization

Centre de recherches mdicales de Lambarn (CERMEL)

Centre de Recherches Mdicales de Lambarn (CERMEL)

Centre for Dengue Research and AICBU, Department of Immunology and Molecular Medicine

Centro de Investigacin en Enfermedades Infecciosas (CIENI), Instituto Nacional de Enfermedades Respiratorias (INER)

Centro de Secuenciacin NASERTIC

Chan-Zuckerberg Biohub

Charite Universitatsmedizin Berlin, Institut fur Virologie/Labor Berlin

CHU Lille

CNR Virus des Infections Respiratoires - France SUD

CQRC_QUALITY CONTROL CHEMICAL BIOLOGICAL RISK_AOOR Villa Sofia Cervello Palermo

CSIR-Centre for Cellular and Molecular Biology - INSACOG

Department for Virology, Molecular Biology and Genome Research, R. G. Lugar Center for Public Health Research, National Center for Disease Control and Public Health (NCDC) of Georgia.

Department of Developmental Medicine, Research Institute, Osaka Women's and Children's Hospital

Department of Infectious Diseases, Kobe Institute of Health

Department of Virology

Department of Virology Nagasaki University

Departments of Pathology and Medicine, New York University School of Medicine

Division of Emerging Infectious Diseases, Bureau of Infectious Diseases Diagnosis Control, Korea Disease Control and Prevention Agency

Erasmus Medical Center

Fulgent Genetics

Gagnon Lab, Southern Illinois University

Genome Centre

GIGA Medical Genomics

Grubaugh Lab - Yale School of Public Health

Gunma Prefectural Institute of Public Health and Environmental Sciences

Hokkaido Institute of Public Health

Hospital General Universitario Gregorio Maran

HUG, Laboratory of Virology and the Health2030 Genome Center

Illinois Department of Public Health - Chicago Lab

Illinois Department of Public Health - Springfield Lab

IN State Department of Health Laboratory Services

inStem NCBS

Institut de Pathologie et Genetique (IPG)

Institute for Developing Science and Health Initiatives (ideSHi)

Institute for Infectious Diseases, University of Bern, Switzerland

Institute for Medical Research, Infectious Disease Research Centre, National Institutes of Health, Ministry

Institute for Medical Research, Infectious Disease Research Centre, National Institutes of Health, Ministry of Health Malaysia
Institute of Applied Biosciences, Centre for Research and Technology Hellas
Institute of Environmental Science and Research (ESR)
Institute of Medical Microbiology and Hospital Hygiene
Institute of Medical Virology
Institute of Microbiology and Immunology, Faculty of Medicine, University of Ljubljana
Institute of Molecular and Translational Medicine / Laboratory of Experimental Medicine, Faculty of Medicine and Dentistry, Palacky University
Institute of Tropical Medicine
Institute of Virology
Institute of Virology at Innsbruck Medical University
Instituto Butantan
Instituto Butantan / ESALQ-USP (Piracicaba)
Instituto Butantan / FZEA-USP (Pirassununga)
Instituto Butantan / Mendelics
Instituto Nacional de Saude (INSA)
Istituto di Genomica Applicata
Istituto Zooprofilattico Sperimentale della Puglia e della Basilicata
Istituto Zooprofilattico Sperimentale dellAbruzzo e Molise G. Caporale
Jessa
Kabara Cancer Research Institute
Kansas Health and Environmental Lab
Labo Klinische Biologie, UZA
Laboratoire de sant publique du Qubec
Laboratoire GenBio
Laboratori de Referencia de Catalunya
Laboratorio Aziendale di Microbiologia e Virologia, Azienda Sanitaria dell'Alto Adige
Laboratorio de Pesquisa em Virologia
Laboratorio di Genomica e Patologia Molecolare
Laboratorio di Microbiologia e Virologia, Universit Vita-Salute San Raffaele, Milano
Laboratory for Clinical Immunology and Molecular Genetics -University Clinic Golnik
Laboratory of Clinical Microbiology, Virology and Bioemergencies, ASST Fatebenefratelli Sacco - Sacco Hospital
Laboratory of genomics and metagenomics
Lauring Lab, University of Michigan, Department of Microbiology and Immunology
LSUHS Emerging Viral Threat Laboratory
Main Chemical Laboratories Egypt Army
Maryland Genomics, Institute for Genome Sciences, University of Maryland School of Medicine
Mathers Lab
MD PHL
MEPHI, Aix Marseille University
Microbial Genome Sequencing Center
Microbiology Department
Ministry of Health Turkey
Molecular Diagnostics
Molecular Diagnostics Malta
MRC/UVRI & LSHTM UgandaResearch Unit
National Institute for Public Health and the Environment (RIVM)
National Institute for Viral Disease Control and Prevention, China CDC
National Institute of Health Research and Development
National Institute of Infectious Diseases-Prof. Dr. Matei Bals Molecular Diagnostics Laboratory
National Institute of Public Health NIH - National Research Institute
National Platform bis UMONS/Jolimont
National Public Health Surveillance Laboratory
National Virus Reference Laboratory
NCDC Delhi, Biotechnology Division
Nevada State Public Health Laboratory
New Mexico Department of Health Scientific Laboratory
New York Genome Center
NIV Influenza

NJ_PHEL
North Dakota Department of Health, Public Health Laboratory
Norwegian Institute of Public Health, Department of Virology
Ohio Department of Health Laboratory
OLVZ Aalst
Omics Sciences Laboratory
Oregon State Public Health Laboratory
OSU Center for Genome Research and Biocomputing
Pathogen Genomics Center, National Institute of Infectious Diseases
PHV-FSS
Public Health Virology-Forensic and Scientific Services
Respiratory Infections Laboratory
Riga East Clinical University Hospital, National Microbiology Reference Laboratory; Eurofins Genomics Europe Sequencing GmbH
Santa Clara County public Health Laboratory
Santa Clara County Public Health Laboratory
Sapporo City Institute of Public Health
School of Public Health, The University of Hong Kong
Seattle Flu Study
Section for Molecular Diagnostics
SeqCOVID-SPAIN consortium/IBV(CSIC)
Son Espases
Sonic Reference Laboratory
State Veterinary Institute Prague
State Virus Research and Diagnostic Laboratory (VRDL), AIIMS Raipur
SVFI, Veterinary institute in Zvolen
SVFI, Veterinary institute in Zvolen, Pod Drahami 918, 960 86 Zvolen Slovakia
SVFI, Veterinary institute in Zvolen, Slovakia
The Jackson Laboratory
The Ohio State University Applied Microbiology Services Laboratory
Tripler Army Medical Center Microbiology Department
TXDSHS
UCLouvain/IREC/MBLG
Uhlemann Laboratory, Columbia University Irving Medical Center
UMR 8199/1283 EGID
UNC Charlotte Environmental Monitoring Laboratory
Universidad Nacional de Colombia - Laboratorio Genmico One Health
University of Bari Biomedical Sciences and Human Oncology
Vilnius University Hospital Santaros Klinikos, Center of Laboratory Medicine
ViroGenetics - BSL3 Laboratory of Virology, Maopolska Centre of Biotechnology, Jagiellonian University;
Virology Laboratory, Scientific Department, Army Medical Center
Virology Unit, Institut Pasteur du Cambodge
Wadsworth Center, New York State Department of Health
Wellcome Sanger Institute for the COVID-19 Genomics UK (COG-UK) Consortium
WHO National Influenza Centre Russian Federation
Wichita State University - Molecular Diagnostics Lab
Wyoming Public Health Laboratory
Wytemaweg 80, 3015 CN Rotterdam
Al-Quds Nutrition and Health Research Institute, Al-Quds University, ABU DEIS, Jerusalem, JERUSALEM 55076, State of Palestine
Animal Health - O.U. Virology, Istituto Zooprofilattico Sperimentale del Mezzogiorno, Via Salute, 2, Portici, Naples 80055, Italy
animal health and puplic health, Escola de Medicina Veterinaria, BR 153 Km 112, Araguaina, Tocantins 77804970, Brazil
ational Key Laboratory of Biochemical Engineering, Institute of Process Engineering, Chinese Academy of Sciences, Haidian District, Beijing 100190, China
Bacteriology, GPHL, 1749 Clairmont Road, Decatur, GA 30033, USA
BDRD Genomics & Bioinformatics, Naval Medical Research Center - Frederick, 8400 Research Plaza, Fort Detrick, MD 21702, USA
Biochemistry and Molecular Biology, University of Texas Medical Branch, 301 University Blvd, Galveston, TX 77555, USA
Biochemistry and Molecular Genetics, Israel Institute for Biological Research, P.O.B 19, Ness-Ziona 74100, Israel
BioInfoExperts, LLC, BioInfoExperts, LLC, 718 Bayou Lane, Thibodaux, LA 70301, USA
Bioinformatics Division, National Institute of Biotechnology, Ganakbari, Ashulia, Savar, Dhaka 1349, Bangladesh
Bioinformatics, Center for Genome Regulation (CRG), Av. Blanco Encalada 2085, 3rd floor., Santiago, Santiago 8320000, Chile

Bioinformatics, National institute of traditional medicine, Indian council for medical research, RMRC Layout, Belgaum, Karnataka 590010, India

Bioinformatics, Wadsworth Center, 150 New Scotland Avenue, Albany, NY 12208, USA

biological prevention, army, sinuris, fayoum, cairo 02, Egypt

Biology, College of Education, University of Garmian, Bardasur Campus, Kalar, Sulaymaniyah, Kurdistant Region 46021, Iraq

Biology, MCL, sinuris, fayoum, cairo 02, Egypt

Biomedical Informatics, UAMS, 4301 W. Markham St., Slot 782, Little Rock, AR 72205, USA

Biomedical Sciences and Public Health, Polytechnic University of Marche, via tronto 10/A, ANCONA, Ancona 60126, Italy

Biomedical Sciences for Health, University of Milan, Via Carlo Pascal, Milan 20133, Italy

Biosafety Department PCL3, Research and Medical Analysis Laboratory of GENDARMERIE ROYALE, Avenue Ibn Sina, Agdal, Rabat 10100, Morocco

BioScience, LANL, PO1663 MS888, Los Alamos, NM 87544, USA

Biotechnologie, MedBiotech laboratory, faculty of medicine and pharmacy, rabat/ Mohamed V university rabat, Mohamed Belarbi El alaoui, Rabat 6203, Morocco

BPHL, FLDOH, 1217 N Pearl St, Jacksonville, FL 32202, USA

Bundeswehr Institute of Microbiology, Bundeswehr Institute of Microbiology, Neuherbergstrasse 11, Munich 80937, Germany

Bureau of Laboratories, Michigan Department of Health and Human Services, 3350 N Martin Luther King Blvd., Lansing, MI 48906, USA

Cancer Biology Department, National Cancer Institute, FOM EL-KHALIG, Cairo, EGYPT 11976, Egypt

CardioVir EA-4684, University of Reims Champagne Ardenne, 51 rue Cognacq Jay, Reims 51100, France

CEIRS Data Processing and Coordinating Center, Center for Research on Influenza Pathogenesis (CRIP), New York, NY 10029-6574, USA

CEIRS Data Processing and Coordinating Center, St. Jude Center of Excellence for Influenza Research and Surveillance (CEIRS), Memphis, TN 38105, USA

Center for Genomics and System Biology, New York University, 12 Waverly Pl, New York, NY 10003, USA

Center for Precision Medicine, Meizhou People's Hospital (Huangtang Hospital), No 63 Huangtang Road, Meijiang District, Meizhou, Guangdong 514031, China

Center of Medical Microbiology, Virology, and Hospital Hygiene, Heinrich Heine University, Universitaetsstr. 1, Dusseldorf 40225, Germany

Central Laboratories, Egyptian Ministry of Health and Population, 19 El-Shaikh Rihan, Ad Dawawin, Abdeen, Cairo, Cairo 11613, Egypt

Central Public Health Laboratories, Egyptian Ministry of Health and Population, 19 El-Shaikh Rihan, Ad Dawawin, Abdeen, Cairo, Cairo 11613, Egypt

Centre For Biotechnology Research and Development, Kenya Medical Research Institute, Mbagathi Road, Nairobi 54840-00200, Kenya

Centre For Biotechnology Research and Development, Kenya Medical Research Institute, Mbagathi Road, Nairobi 54840, Kenya

Centre for Human Virology & Genomics, Nigerian Institute of Medical Research, 6, Edmund Crescent, Yaba, Lagos, Lagos 101212, Nigeria

Centre for Infectious Disease Control, CDC of PLA, 20 DongDa Street, Fengtai District, Beijing, Beijing 100071, China

Centre of Nanotechnologies, INCD IMT-Bucuresti (National Institute for Research and Development in Microtechnologies - Bucharest), Erou Iancu Nicolae 126 A, Bucharest, Voluntari 77190, Romania

Centro de Desenvolvimento Tecnologico em Saude, Fundacao Oswaldo Cruz, Avenida Brasil, Rio de Janeiro, Rio de Janeiro 21040900, Brazil

Civil Engineering, The University of Hong Kong, Pokfulam Road, HongKong 000000, China

Clinical Diagnostics and Research Center, 'Central Research Institute of Epidemiology' of The Federal Service on Customers' Rights Protection and Human Well-being Surveillance, Novogireevskaya, Moscow 111123, Russia

Clinical Laboratory Sciences, University of Babylon, Al-Tagia, Hilla, Babil 51001, Iraq

Clinical Laboratory, Hospital Israelita Albert Einstein, Av. Albert Einstein, 627/701, Sao Paulo, SP 05652-000, Brazil

Clinical virology, Institut Pasteur de Tunis, 13 place Pasteur le belvedere BP: 74, Tunis, *Sans objet 1002, Tunisia

CMBC, IVIC, Km 11 Panamericana IVIC, CARACAS, Miranda 1020A, Venezuela

CMBC, IVIC, Km 11 Panamericana, CARACAS, Miranda 1020A, Venezuela

CMBC, IVIC, Km11 Panamericana, Caracas, Miranda 1020A, Venezuela

College of life sciences, Anhui Medical University

College of Pharmacy, University of South Carolina, 715 Sumter Street, Columbia, SC 29208, USA

College of Veterinary Medicine, Chungnam National University, 220 Gung Dong, YuseongGu, Daejeon 34134, South Korea

Communicable Disease Laboratory, Public Health Directorate, 1124, Manama 12, Bahrain

Communicable Diseases, Interactive Research School for Health Affairs, Bharati Vidyapeeth (Deemed to be University), Pune Satara Road, Katraj, Pune, Maharashtra 411043, India

Computer Science and Engineering, University of Louisville, Duthie Center Room 208, Louisville, KY 40292, USA

Contact:Hiroyuki Asakura Tokyo Metropolitan Institute of Public Heaith, Department of Microbiology; 3-24-1 Hyakunincho, Shinjyuku, Tokyo 169-0073, Japan URL :http://www.tokyo-eiken.go.jp/

Contact:Hiroyuki Asakura Tokyo Metropolitan Institute of Public Health, Department of Microbiology; hyakunincho3-24-1, Shinjyuku-ku, Tokyo 169-0073, Japan URL :http://www.tokyo-eiken.go.jp/

Contact:Hiroyuki Asakura Tokyo Metropolitan Institute of Public Health, Department of Microbiology; Hyakunincho3-24-1, Shinjyuku-ku, Tokyo 169-0073, Japan URL :http://www.tokyo-eiken.go.jp/

Contact:Junko Tanaka Graduate School of Biomedical and Health Sciences, Hiroshima University, Department of Epidemiology, Infectious Disease Control and Prevention; 1-2-3, Kasumi, Minami Ku, Hiroshima, Hiroshima 734-8551, Japan

Contact:Ryota Kumagai Tokyo Metropolitan Institute of Public Health, Department of Microbiology; 3-24-1 Hyakunin-cho, Shinjuku-ku, Tokyo 169-007, Japan URL :http://www.tokyo-eiken.go.jp/

Contact:Ryota Kumagai Tokyo Metropolitan Institute of Public Health, Department of Microbiology; 3-24-1 Hyakunin-cho, Shinjuku-ku, Tokyo 169-0073, Japan URL :http://www.tokyo-eiken.go.jp/

Contact:Tsuyoshi Sekizuka National Institute of Infectious Diseases, Pathogen Genomics Center; 1-23-1 Toyama, Shinjuku-ku, Tokyo 162-8640, Japan

Core Sequencing, Maryland Department of Health, 1, 1, MD 1, USA

CoronaNet Lab- TaskForce Regione Campania, CEINGE Biotecnologie Avanzate, Via G. Salvatore, 486, Naples 80145, Italy

COVID-19 Task Force, Cairo University, Qasr El-Ainy Street, Cairo 11562, Egypt

Covid lab center, Yaftabad Hospital, Southern Al-Ghadir Blvd., Al-Ghadir Sq., beginning of Saveh Road, Tehran, Tehran 1449614535, Iran

CSL, Maryland Department of Health, 1, 1, MD 1, USA

CSL, Maryland Department of Health, 1770 Ashland Ave, Baltimore, MD 21205, USA

Csl, Mdh, 1, 1, MD 1, USA

Data Science, CZ Biohub, 499 Illinois St, San Francisco, CA 94158, USA

Department of Biochemistry, Cell and Molecular Biology, WACCBIP, University of Ghana, Old Volta Road, University of Ghana, Legon, Accra, Greater Accra +233, Ghana

Department of Biochemistry, Cell and Molecular Biology, WACCBIP, University of Ghana, Volta Road, University of Ghana, Legon, Accra, Greater Accra +233, Ghana

Department of Biology and Wildlife, Alaska State Virology Laboratory, 1051 Sheenjek Dr, Fairbanks, AK 99709, USA

Department of Biomedical Science and Human Oncology, University of Bari "Aldo Moro", Bari, Italy, Piazza G. Cesare 11, Policlinico, BARI 70124, Italy

Department of Biomedical, Surgical and Dental Sciences and Department of Biomedical Sciences for Health, Universita degli Studi di Milano, Via Carlo Pascal 36, Milan 20133, Italy

Department of Experimental Modeling and Pathogenesis of Infectious Diseases, Federal Research Center of Fundamental and Translational Medicine, Timakov 2, Novosibirsk 630117, Russia

Department of General Services Division of Consolidated Laboratory Services, Virginia Division of Consolidated Laboratory Services Sequencing Submission Group, 500 North 5th Street, Richmond, VA 23219, USA

Department of Health Technology and Informatics, The Hong Kong Polytechnic University, 11 Yuk Choi Rd, Hong Kong 852, Hong Kong

Department of Health, Utah Public Health Laboratory, 4431 2700 W, Salt Lake City, UT 84129, USA

Department of Immunology, The Scripps Research Institute, 10550 N Torrey Pines Rd, San Diego, CA 92122, USA

Department of Infection Prevention and Infectious Diseases, University Hospital Regensburg, Franz-Josef-Strauss Allee 11, Regensburg 93053, Germany

Department of Infectious Disease Prevention and Control, Henan Provincial CDC, Agricultural South Road Road, Zhengzhou, Henan 450016, China

Department of Infectious Diseases, Gothenburg University, Guldhedsgatan 10B, Gothenburg 41346, Sweden

Department of Laboratory Medicine, Lin-Kou Chang-Gung Memorial Hospital, No. 5, Fu-Shing St., Taoyuan 333, Taiwan

Department of Medical Laboratory Sciences, Arab American University, Jenin, Jenin, Palestine 91220, West Bank

Department of Medical Microbiology, Kafkas University, Faculty of Medicine, Kafkas University, Faculty of Medicine, Department of Medical Microbiology, Kars 36100, Turkey

Department of Medical Microbiology, Kafkas University, Faculty of Medicine, Kafkas University, Kars 36100, Turkey

Department of Medical Microbiology, Leiden University Medical Center, LUMC E4-P, room E-01-201m, Albinusdreef 2, Leiden 2333 ZA, The Netherlands

Department of Microbiology and Immunology, Stony Brook University, 100 Nicolls Rd Rm 126, STONY BROOK, NY 11790, USA

Department of Microbiology, Second Military Medical University, 8 Panshan Road, Yangpu District, shanghai 200433, China

Department of microbiology, the University of Hong Kong, 102 Pokfulam Road, Pokfulam, Hong Kong 999077, Hong Kong

Department of Microbiology, Zhejiang Provincial CDC, 3399 Binsheng Road, Hangzhou, Zhejiang 310051, P.R. China

Department of Molecular Medicine, University of Padova, via Gabelli 63, Padova 35121, Italy

Department of Science and Technology, Gujarat Biotechnology Research Centre, MS Building, Block B & D, 6th Floor, GH Road, Sector - 11, Gandhinagar, Gujarat 382011, India

Dept. of Pediatrics and Dept. Medical Microbiology, 2nd Faculty of Medicine, Charles University in Prague, V Uvalu 84, Prague 15006, Czech Republic

Dipartimento di Biotecnologie Mediche, University of Siena, viale Bracci 16, Siena 53100, Italy

Director General, Nigerian Institute of Medical Research, 6, Edmund Crescent, Yaba, Lagos 101212, Nigeria

Division of Infectious Diseases, Department of Medicine, CZ Biohub, UCSF, 499 Illinois St, San Francisco, CA 94158, USA

Division of Pathogen Resource Management, Korea National Institute of Health, Korea Disease Control and Prevention Agency, #200 Osongsaengmyeong 2-ro, Osong-eup, Heungdeok-gu, Cheongju-si, Chungcheongbuk-do 28160, South Korea

Division of Viral Diseases, CDC Pathogen Discovery Team, 1600 Clifton Road, Atlanta, GA 30329, USA

DPH, Massachusetts State Public Health Lab, 305 South, Boston, MA 02130, USA

Emergence des Maladies Virales, Centre Interdisciplinaire de Recherches Medicales de Franceville, CIRMF, Franceville BP769, Gabon

Environmental and Global Health, University of Florida, 1225 Center Drive, HPNP Bldg. Room 4155, Gainesville, FL 32610-0188, USA

Environmental and Global Health, University of Florida, Gainesville, 1225 Center Drive, HPNP Bldg. Room 4155, Gainesville, FL 32607, USA

Environmental and Global Health, University of Florida, Gainesville, 1225 Center Drive, HPNP Bldg. Room 4155, Gainesville, FL 32610-0188, USA

Environmental and Global Health, University of Florida, Gainesville, 1225 Center Drive, HPNP Bldg. Room 4155, Gainesville, FL 32610, USA

Environmental and Global Health, University of Florida, Gainesville, 204 NW 32nd St, Gainesville, FL 32607, USA

Environmental, Agricultural, and Occupational Health, University of Nebraska Medical Center, Wittson Hall lab 4032, Omaha, NE 68198, USA

Epidemiology of Microbial Diseases, Yale School of Public Health, 60 College Street, New Haven, CT 06510, USA

Faculty of Medicine, Al-Quds University, ABU DEIS, Jerusalem, JERUSALEM 55076, State of Palestine

Faculty of Medicine, Bursa Uludag University, Izmir yolu, Bursa 16120, Turkey

Federal Service on Consumers' Rights Protection and Human Well-Being Surveillance, Plague Control Center, Musorgskogo, 4, Moscow 127490, Russia

Genomic Research Lab, Bangladesh Council of Scientific and Industrial Research, Dr. Qudrat-E-Khuda Road, Dhaka 1205, Bangladesh

Genomic Research Lab, Bangladesh Council of Scientific and Industrial Research, Dr. Qudrat-E-Khuda Road, Dhaka, VIC 1205, Bangladesh

Genomic Sciences, Rehman Medical Institute, Peshawar, Pakistan, 5-B/2, Phase V, Hayatabad, Peshawar, Khyber Pakhtunkhuwa 25000, Pakistan

Genomics Program, Children Cancer Hospital, Zeinhom, El-Sayeda Zainab, Cairo Governorate, Cairo 11562, Egypt

Gonoshasthaya-RNA Research Center, Gonoshasthaya-RNA Molecular Diagnostics and Research Center, House 14/E, Road 6, Dhaka 1205, Bangladesh

Group of Genetic Engeneering and Biotechnology, Federal Budget Institution of Science 'Central Research Institute of Epidemiology' of The Federal Service on Customers' Rights Protection and Human Well-being Surveillance, Novogireyevskaya Street, 3a, Moscow 111123, Russia

Human Genome Variation Research Group, Malopolska Centre of Biotechnology, Gronostajowa 7A, Krakow 30-387, Poland

Infection and Immunology, Translational Health Science and Technology Institute, 3rd Milestone, Faridabad Gurgram Expressway, PO Box 04, Faridabad, Haryana 121001, India

Infectious Disease Control Center, CDC of PLA, 20 DongDa Street, Fengtai District, Beijing, Beijing 100071, China

Infectious Disease Program, Broad Institute of Harvard and MIT, 75 Ames St, Cambridge, MA 02142, USA

Infectious Diseases and Tropical Medicine Research Center, Infectious Diseases and Tropical Medicine Research Center, Isfahan University of Medical Sciences, Isfahan, Iran., Hezar jarib, Isahan, Isfahan 12345, Iran

Infectious Diseases Research, King Abdullah International Medical Research Center (KAIMRC), KAIMRC, P.O. Box 22490, Riyadh 11426, Saudi Arabia

Infectious diseases, Chan Zuckerberg Biohub, 499 Illinois st., San Francisco, CA 94158, USA

Infectious Diseases, NC SLPH COVID-19 Response Team, 4312 District Dr, Raleigh, NC 27699, USA

Infectious Diseases, Quest Diagnostics, 33608 Ortega Hwy, San Juan Capistrano, CA 92675, USA

Institute for Computational Biomedicine, Weill Cornell Medicine, 1305 York Ave, New York, NY 10065, USA

Institute for Forensic Medicine, Faculty of Medicine, University of Belgrade, Deligradska 31a, Belgrade, Serbia 11000, Serbia

Institute of Clinical Microbiology and Hygiene, University Hospital Regensburg, Franz-Josef-Strauss-Allee 11, Regensburg 93053, Germany

Institute of Human Genetics, Polish Academy of Sciences, Institute of Human Genetics, Polish Academy of Sciences, Strzeszynska 32, Poznan 60-479, Poland

Institute of Infection and Global Health, University of Liverpool, 146 Brownlow Hill, Liverpool L3 5RF, United Kingdom

Institute of Microbiology, University of Veterinary and Animal sciences, Out Fall road, Lahore, Punjab 54600, Pakistan

Institute of Molecular Biology and Biotechnology, National Institutes of Health, 623 Pedro Gil, Ermita, Manila 1000, Philippines

Institute of Virology, Charite, chariteplatz 1, Berlin, Berlin 10117, Germany

KAROLINSKA INSITUTET, Department of Cell and Molecular Biology, Solnavagen 9, 17165 Stockholm, Sweden

Laboratorio de Infecciones Respiratorias Agudas. Centro Nacional de Salud Publica, Instituto Nacional de Salud, Capac Yupanqui 1400 - Jesus Maria, LIMA, LIMA LIMA 01, Peru

Laboratorio Specialistico - UOC Hematology, ASSL Nuoro, via Manniròni 1, Nuoro, Nuoro 08100, Italy

Laboratory Diagnostic, Veterinary Specialized Institute Kraljevo, Zicka 34, Kraljevo 36103, Serbia

Laboratory Medicine, University of California San Francisco, 600-16th Street, San Francisco, CA 94158, USA

Laboratory Medicine, University of Washington, 1100 Fairview Avenue North, E5-110, Seattle, WA 98109, USA

Laboratory of Clinical Microbiology, Virology and Biomergencies, ASST Fatebenefratelli Sacco, L. Sacco University Hospital, via G.B. Grassi, 74, Milano, MI 20157, Italy

Laboratory Services Section, Texas Department of State Health Services-SARS-CoV-2 submission group, 1100 W. 49th St, Austin, TX 78756, USA

Laboratory, The Bio Arte Limited, Life Science Park, San Gwann 9012, Malta

Los Angeles County Public Health Laboratories, Los Angeles County Public Health Lab microbial pathogen submission group, 12750 Erickson Avenue, Downey, CA 90242, USA

Maximum Containment Laboratory, National Institute of Virology, Pashan, Pune, Maharashtra 411021, India

MDU-PHL, The Peter Doherty Institute for Infection and Immunity, 792 Elizabeth Street, Melbourne, Vic 3000, Australia

Medical Ain Shams Research Institute (MASRI), Faculty of Medicine, Ain Shams University, Ramsis Street, Abbassia Square, Cairo 11566, Egypt

Medical Laboratory Sciences, Arab American University, Jenin, Jenin, Jenin 91220, State of Palestine

Medical Microbiology, Leiden University Medical Center, Albinusdreef 2, Leiden, South Holland 2333ZA, Netherlands

Medicine and Surgery, University of Insubria, Via Guicciardini, Varese, VA 21100, Italy

Medicine and Surgery, University of Insubria, viale borri 57, 21100, varese, it 21100, Italy

Microbiology & Bioinformatics and Biostatistics, Kohat University of Science and Technology & Shanghai Jiao Tong University, China, Bannu Road, Near Jarma Bridge, Kohat, Khyber Pakhtunkwa 25000, Pakistan

Microbiology & Immunology, University of North Carolina, 450 West drive, chapel hill, NC 27514, USA

Microbiology & Immunology, University of North Carolina, 450 West Drive, Chapel Hill, NC 27514, USA

Microbiology and Immunology, The Peter Doherty Institute for Infection and Immunity, 792 Elizabeth Street, Melbourne, Vic 3000, Australia

Microbiology and Immunology, University of South Alabama, 610 Clinic Drive, LID, Mobile, AL 36688, USA

Microbiology Division, SC DHEC, 8231 Parklane Rd, Columbia, SC 29223, USA

Microbiology, Antimikrop ARGE ve Biyosidal Analiz Merkezi, Nasuh Akar Mh. Suleyman Haciabdullahoglu Cd. 37/1, Ankara 06520, Turkey

Microbiology, Canterbury Health Laboratories, cnr Tuam St and Hagley Ave, Christchurch 8011, New Zealand

Microbiology, Charles Nicolle Hospital, National Influenza Center, University of Tunis ElManar, Faculty of Medicine, LR99ES09, LR16SP02, LR01ES04, LR18SP03, LR99ES10, LR 16IPT09, Tunis, Tunisia, Boulevard 9 avril 1938, Tunis, Tunis 1006, Tunisia

Microbiology, Colorado Department of Public Health and Environment, 8100 E Lowry Blvd, Denver, CO 80230, USA

MICROBIOLOGY, ICMR-REGIONAL MEDICAL RESEARCH CENTRE, CHANDRASEKHARPUR, BHUBANESWAR, ODISHA 751023, India

Microbiology, Immunolgy and Transplantation, KU Leuven Rega Institute, Herestraat 49 BOX1040, Leuven 3000, Belgium

Microbiology, Infectious Diseases and Immunology, Centre de Recherche du Centre Hospitalier de l'Universite de Montreal, 900 Saint Denis Street, Montreal, Quebec H2X 0A9, Canada

Microbiology, Koc University, Davutpasa Caddesi, Istanbul 34010, Turkey

Microbiology, ND Dept of Health, 2635 E Main, Bismarck, ND 58501, USA

Microbiology, UNIVERSITY HOSPITAL DONOSTIA, PASEO DR BEGUIRISTAIN, SAN SEBASTIAN, GIPUZKOA 20014, Spain

Microbiology, University of Pennsylvania, 425 Johnson Pavilion, 3610 Hamilton Walk, Philadelphia, PA 19104, USA

Mikrobiologie, RARI, Universitetskaya 46, Saratov, Saratov region 410005, Russia

Molecular and Cell Biology, Globe Biotech Limited, 3/Ka (New), Tejgaon Industrial Area, Dhaka, Dhaka 1208, Bangladesh

Molecular and Cellular Screening Process Laboratory, Center of Biotechnology of Sfax, Sidi Mansour, Sfax 3018, Tunisia

Molecular Biology, IZS Sicilia, Via Gino Marinuzzi, Palermo 90129, Italy

Molecular Biology, New Mexico Department of Health Scientific Laboratory, 1101 Camino de Salud NE, Albuquerque, NM 87102, USA

Molecular Diagnostics, Central Public Health Laboratory, Safin Road, Erbil, Arbil, Iraq

MOLECULAR GENETICS, HUCA, GENETICA HUCA, OVIEDO, ASTURIAS 33011, Spain

Molecular Microbiology & Immunology, University of Missouri, 1201 Rollins St, Columbia, MO 65211, USA

Molecular Microbiology of Laboratory, Institute of Biotechnology, Vietnam Academy of Science and Technology, Viet Nam, 18, Hoang Quoc Viet street, Ha Noi 100000, Viet Nam

Molecular Virology, Instituto Carlos Chagas / Fiocruz PR, Rua Professor Algacyr Munhoz Mader 3775, CIC., Curitiba, PR 81350010, Brazil

Molecular, Florida Bureau of Public Health Laboratories, 1217 N. Pearl Street, Jacksonville, FL 32202, USA

Molecular, ProgenaBiome, 1845 Knoll Dr, Ventura, CA 93003, USA

National Reference Center for transfusion infectious risks, INTS, 6, rue Alexandre Cabanel, PARIS 75015, France

Neurovirology, National Institute of Mental Health and Neurosciences, Hosur Road, Bangalore 560029, India

NGS Lab, DNA SOLUTION LTD., Panthapath, Dhaka, state 1205, Bangladesh

Nigerian Institute of Medical Research, Nigerian Institute of Medical Research, 6, Edmund Crescent, Off Murtala Mohammed Way, Yaba, Lagos State 234-101212, Nigeria

Oklahoma Animal Disease Diagnostic Laboratory, Oklahoma State University, 1950 W Farm Road, Stillwater, OK 74078, USA

Pathology and Laboratory Medicine, Emory University School of Medicine, 101 Woodruff Circle, Atlanta, GA 30322, USA

Pathology and Laboratory Medicine, UW-Madison, 555 Science Drive, Madison, WI 53711, USA

Pharmaceutical Sciences Department - School of Pharmacy, Lebanese American University, Blat, Byblos 1401, Lebanon

Physiology, Istanbul Medeniyet University, Dumlupinar D100 Karayolu No:98, Istanbul 34720, Turkey

Population Medicine and Diagnostic Sciences, Cornell University, 240 Farrier Rd, Ithaca, NY 14853, USA

Prof. Massimo Zollo CEINGE TASK-FORCE COVID19 - Regione Campania, CEINGE Biotecnologie Avanzate, Via G. Salvatore, 486, Naples 80145, Italy

Public Health Laboratory - Infectious Disease Lab, Minnesota Department of Health Infectious Disease Laboratory Submission Group, 601 Robert St. N, St. Paul, MN 55164, USA

Public Health, United States Air Force School of Aerospace Medicine, 2510 Fifth St., Bldg 840, Wright-Patterson AFB, OH 45433-7913, USA

R&D, Pirogov Russian National Research Medical University, Ostrovityanova 1, Moscow 117997, Russia

Rapid Response Team, Pasteur Institute of Iran, 12th Farvardin, Tehran 1316943551, Iran

Rapid Response Team, Pasteur Institute of Iran, 12th Farvardin, Tehran 131943551, Iran

Research and Experiment Center, Meizhou People Hospital, 63 Huangtang Road, Meizhou, Guangdong 514031, China

Research Center Of Tropical and Infectious Of Medical Sciences, Kerman, IranDiseases Kerman University, SARS-COV2, Jomhury BLvd., kerman, kerman 7616913555, Iran

Research institute for Biotechnology and Bio-engineering, Isfahan University of Technology, Imam Khomeini, Isfahan, Isfahan 8415683111, Iran

Research Laboratories, King Fahad Medical City, Dabab street, Suleimaniyah, Riyadh 11522, Saudi Arabia

Research platform for Transfusion-transmitted Disease, Institute of Blood Transfusion, Chinese Academy of Medical Sciences, Chenghua District Huacai Road, Chengdu 610052, China

Respiratory virus Laboratory, Chinese Academy of Medical Science, alternating current road 935, Kunming, Yunnan 650221, China

Respiratory Viruses Branch, CDC, 1600 Clifton Rd, Atlanta, GA 30329, USA

RTS-Genomics, NIAID, 903 S. 4th St, Hamilton, MT 59840, USA

Russian Academy of Sciences, Federal Research Center for Virology and Microbiology, Academician Bakoulov Street, Volginsky, Vladimir region 601125, Russia

School of Pharmacy, Shenandoah University, 1775 N. Sector Court, Winchester, VA 22601, USA

School of Public Health, The University of Hong Kong, 21 Sassoon Road, Pokfulam NA, Hong Kong

School of Veterinary Medicine, Disease Control, The University of Zambia, Great east road, Lusaka, Lusaka 10101, Zambia

Science Department, SRCAMB, Building 1, Obolensk, Moscow region 142279, Russia

Special Infectious Agents Unit, King Fahd Medical Research Center, King Abdulaziz University, University St, Jeddah, Jeddah 21589, Saudi Arabia

Special Operations Medical Research Division, Defence Services Medical Research Centre, DSMRC, Nay Pyi Taw 15013, Myanmar

Tewhey Lab, The Jackson Laboratory, 600 Main Street, Bar Harbor, ME 04609, USA

U de Chile, Animal Virology Laboratory, 11735 Santa Rosa, Santiago 8820808, Chile

UDTI, InDRE, Francisco de P. Miranda 177 bis Lomas de Plateros, Mexico, Mexico 01480, Mexico

UMR 190 - Faculte de medecine, UMR 'Emergence des Pathologies Virales' (EPV: Aix-Marseille University - IRD 190 - Inserm 1207 - EHE, 19-21 boulevard Jean Moulin 5eme etage aile bleue, Marseille 13005, France

UMR190 Unite des Virus Emergents U1207, IHU mediterranee, 19-21 boulevard Jean Moulin, Marseille 13005, France

Unidad de Investigaciones en Biomedicina, Zurita & Zurita Laboratorios, Av. Prensa N49-221, Quito, Pichincha 170104, Ecuador

Unite des virus emergents, UMR190, 27 BD JEAN MOULIN, faculty of medecine, Marseille, Bouches du Rhone 13005, France

University Clinical Research Center, University of Sciences, Techniques and Technologies of Bamako, Point G, Bamako ML91093, Mali

University of Washington Medical Center, Seattle Flu Study, 1959 NE Pacific Street, Seattle, WA 98195, USA

URMITE, IHU - Mediterranee Infection, 19-21 Boulevard Jean Moulin, 13005 Marseille, France

Utah Public Health Laboratory, Utah Public Health Laboratory Infectious Disease submission group, 4431 S 2700 W, Salt Lake City, UT 84129, USA

Viral Hemorrhagic Fever Program, Kenema Government Hospital, 1 Combema Road, Kenema, Kenema, Sierra Leone

Viral vaccines, VSVRI- Veterinary serum and vaccine research institute, El sekka elbedaa street, Cairo, Abbasia 11535, Egypt

Virology (microbiology), HUCA, Av. Roma, Oviedo, Asturias 33011, Spain

Virology Laboratory, INMI L. Spallanzani, Via Portuense 292, Rome, Rome 00149, Italy

VIROLOGY, AFRIMS, 315/6 Ratchawithi, Ratchatewi, Bangkok 10400, Thailand

Virology, CHU Pitie Salpetriere Charles Foix, 47/83 boulevard de l'hopital, Paris 75013, France

Virology, Ecole Nationale Veterinaire de Toulouse, 23 chemin des Capelles, Toulouse 31300, France

Virology, Hamadan university of medical sciences, Shahid fahvideh Blvd, Hamadan 08133333333, Iran

Virology, icddr, b, 68, Shaheed Tajuddin Ahmed Sarani, Dhaka, Dhaka 1212, Bangladesh

Virology, Iran University of Medical Sciences, Tehran Hemat Highway next to Milad Tower, Tehran, Tehran 14535, Iran

Virology, Mechnikov Research Institute for Vaccines & Sera, Malyi Kazennyi pereulok 5a, Moscow 105064, Russia

Virology, Mechnikov Research Institute of Vaccines & Sera, Malyi Kazennyi pereulok 5a, Moscow 105064, Russia

Virology, National Institute for Biological Standards and Control, Blanche Lane, South Mimms EN6 3QG, United Kingdom

Virology, Saman gene Arad, Sadoghi, qom 3716647968, Iran

virology, tehran university of medical sciences, Poursina, Tehran 1417613151, Iran

Virus Ecology, NIH, 903 S 4th street, Hamilton, MT 59840, USA

WHO WPRO Regional Polio Reference Laboratory, National Institute for Viral Disease Control and Prevention, Chinese CDC, No155, Changbai road, Changping District, Beijing, Beijing, China 102206, China

HOSPITAL UNIVERSITARY 12 DE OCTUBRE, Microbiology, Avenida de Cordoba sn 28041 Madrid, Spain

[Romania, Bucharest] National Institute for Infectious Diseases Prof. Dr. Matei Bal

1-Clinical and Experimental Pharmacology Lab, LR16SP02, National Center of Pharmacovigilance, University of Tunis El Manar, Tunis, Tunisia. 2-Neurodegenerative diseases and psychiatric troubles, LR18SP03, Razi Hospital, University of Tunis El Manar, Tunis, Tunisia. 3- Ministry of Health, National Observatory of New and Emerging Diseases, 1006, Tunis, Tunisia

1. Academic Center for Pathomorphological and Genetic-Molecular Diagnostics ltd, Bialystok, Poland 2. National Institute of Public Health - National Institute of Hygiene, Warsaw, Poland

1. Tricity SARS-CoV-2 sequencing consortium: University of Gdansk, Medical University of Gdansk, Vaxican Ltd., Invicta Ltd. 2. National Institute of Public Health - National Institute of Hygiene, Warsaw, Poland

Wojewdzka Stacja Sanitarno-Epidemiologiczna w Katowicach

TaskForce Covid19-Ceinge Regione Campania

1. ViroGenetics - BSL3 Laboratory of Virology, Maopolska Centre of Biotechnology, Jagiellonian University; 2. Human Genome Variation Research Group, Malopolska Centre of Biotechnology, Jagiellonian University;

1. ViroGenetics - BSL3 Laboratory of Virology, Maopolska Centre of Biotechnology, Jagiellonian University; 2. genXone SA, Research & Development Laboratory

1. ViroGenetics - BSL3 Laboratory of Virology, Maopolska Centre of Biotechnology, Jagiellonian University; 2. genXone SA, Research & Development Laboratory

1. ViroGenetics - BSL3 Laboratory of Virology, Maopolska Centre of Biotechnology, Jagiellonian University; 2. Diagtron Laboratoria ukasz Rbalski

1. ViroGenetics - BSL3 Laboratory of Virology, Maopolska Centre of Biotechnology, Jagiellonian University; 2. Diagtron Laboratoria Lukasz Rabalski;

1. National Institute of Public Health - National Institute of Hygiene, Warsaw, Poland 2. Biobank Lab, University of Lodz 3. Laboratory of Respiratory Viruses, Teaching and Clinical Center of the Medical University of Lodz

1. ViroGenetics - BSL3 Laboratory of Virology, Maopolska Centre of Biotechnology, Jagiellonian University; 2. II Department of Internal Medicine, Faculty of Medicine, Jagiellonian University Medical College.

1. ViroGenetics - BSL3 Laboratory of Virology, Maopolska Centre of Biotechnology, Jagiellonian University; 2. II Department of Internal Medicine, Faculty of Medicine, Jagiellonian University Medical College; 3. Narodowy Instytut Zdrowia Publicznego Pastwowy Zakad Higieny (NIZP PZH)

1. ViroGenetics - BSL3 Laboratory of Virology, Maopolska Centre of Biotechnology, Jagiellonian University; 2. II Department of Internal Medicine, Faculty of Medicine, Jagiellonian University Medical College; 3. Narodowy Instytut Zdrowia Publicznego Pastwowy Zakad Higieny (NIZP PZH)

1. Genome Research Center for Health (CRGS) / 2. Laboratory of Molecular Medicine and Genomics(LMMGe) / 3. Center for Research in Pure and Applied Mathematics (CRMPA)

National Institute of Public Health - National Institute of Hygiene

Wojewodzka Stacja Sanitarno-Epidemiologiczna w Gorzowie Wielkopolskim

National Health Laboratory Service/UCT

NHLS/UCT

IRBA, 2MI

Central National Laboratory ,Public Health Organization

1. National Institute of Public Health - National Institute of Hygiene; 2. Eurofins Genomics Europe Sequencing GmbH

WVU and Marshall University Combined Genomics Core Facilities

NSW Health Pathology - Institute of Clinical Pathology and Medical Research; Westmead Hospital; University of Sydney

54gene Molecular Genetics Laboratory, Nigeria

Elling and Cochella laboratories, IMBA/IMP

Charit Universittsmedizin Berlin, Institut fr Virologie

Charit Universittsmedizin Berlin, Institute of Virology

Charite Universitatsmedizin Berlin, Institut fur Virologie

National Institute for Infectious Diseases (INMI) L. Spallanzani I.R.C.C.S

A.O.U Policlinico G.Rodolico-S.Marco P.O. Rodolico and BIOMETEC UNICT Catania

Fondazione del Piemonte per l'Oncologia IRCCS

U.O. Genomics, S.S. Genetics and Advanced Omics Techniques, Istituto Zooprofilattico Sperimentale del Piemonte Liguria e Valle d'Aosta

Department of General Diagnostics; Department of Virology; Istituto Zooprofilattico Sperimentale del Lazio e della Toscana (IZSLT)

The Public Health Agency of Sweden
Indira Gandhi Memorial Hospital
National Institute for Communicable Diseases of the National Health Laboratory Service
Instituto Nacional de Saude (INSA) and Faculdade de Medicina da Universidade de Coimbra
Instituto Nacional de Saude (INSA) and Institute of Biomedicine (iBiMed), Universidade de Aveiro
Central Biological Research Laboratory and Department of Biochemistry and Molecular Biology
University Hospital Basel, Clinical Bacteriology
Centre Muraz
Centre de Recherches Mdicales de Lambarn
RSE National Center of Expertise and RSE National center for Biotechnology
Academic Center for Pathomorphological and Genetic-Molecular Diagnostics ltd, Bialystok, Poland
Genetics Working Group (Pokja Genetik) Faculty of Medicine, Public Health and Nursing Universitas Gadjah Mada (FK-KMK UGM); Disease Investigation Center Wates Ministry of Agriculture Indonesia; Department of Microbiology FK-KMK UGM; Laboratorium Diagnostik Yayasan Tahija World Mosquito Program (WMP) Yogyakarta Center for Tropical Medicine FK-KMK UGM; Integrated Research center FK-KMK UGM; Department of Computer Science and Electronics FMIPA UGM
Access DX
CERBA HealthCare
Acibadem Mehmet Ali Aydinlar University School of Medicine, Medical Genetics Department
INMI Lazzaro Spallanzani IRCCS
Cerba lab
Schwessinger Lab
Idaho Bureau of Laboratories
Cancer Control Center Center
Kuwait cancer Control Center
Kuwait Cancer control Center
Kuwait Cancer Control Center
Virology Unit, Department of Microbiology, Faculty of Medicine, Kuwait University, Kuwait
Philippine Genome Center
Institute of Tropical Disease, Universitas Airlangga
Laboratorio Departamental de Salud Publica de Antioquia
Laboratory of genomics and metagenomics, Institute of Microbiology, University Hospital Centre and University of Lausanne, Switzerland
Genomics and Transcriptomics, Philip Morris International
Robert Koch Institute
Retrovirus Laboratory Adolfo Lutz Institute
ADPH
Virology Laboratory, Department of Microbiology, King George's Medical University
Genomic Research Lab, BCSIR
Advanced Molecular Diagnostics, Arizona Department of Health Services
Faculty of Medicine, Universitas Sumatera Utara; Institute of Tropical Disease, Universitas Airlangga
Los Angeles County PHL
Instituto Adolfo Lutz, Interdiciplinary Procedures Center, Strategic Laboratory
Institut fr Hygiene und Umwelt Hamburg
Heinrich Pette Institute, Leibniz Institute for Experimental Virology
KRISP, KZn Research Innovation and Sequencing Platform
CERI, Centre for Epidemic Response and Innvoation, Stellenbosch University and KRISP, KZN Research Innovation and Sequencing Platform, UKZN
African Centre of Excellence for Genomics of Infectious Diseases (ACEGID), Redeemer's University, Ede, Osun State, Nigeria
Istitituto Zooprofilattico Sperimentale dell'Abruzzo e Molise 'G.Caporale'
Greek Genome Center, Biomedical Research Foundation of the Academy of Athens (BRFAA)
KRISP, KZN Research Innovation and Sequencing Platform
KRISP, KZN Research Innovation and Sequencing Platform
Instituto Nacional de Salud- Direccin de Investigacin en Salud Pblica
AIDS Vaccine Research Laboratories
Irish Coronavirus Sequencing Consortium-Teagasc Grange
Irish Coronavirus Sequencing Consortium - National University of Ireland Galway
Irish Coronavirus Sequencing Consortium - National Virus Reference Laboratory
Institute of Life Sciences - INSACOG
Virology Department,Central Health Laboratory
UMR PIMIT
Virology Department, Central Health Laboratory ,Victoria Hospital, Candos,Ministry of Health and Wellness, Mauritius

Genomics and Discovery, Respiratory Viruses Branch, Division of Viral Diseases, CDC
Respiratory Viruses Branch, Division of Viral Diseases, CDC
Akademiska Sjukhuset, Clinical Microbiology and Hospital Hygiene
Dept. of Laboratory Medicine
AKUH Laboratories, Karachi, Pakistan
Al-Quds Nutrition and Health Research Institute, Al-Quds University
West Java Health Laboratory; School of Life Sciences and Technology, Institut Teknologi Bandung
Al Jalila Childrens Hospital
Al Jalila Genomics Center
WSSEw Warszawie
1. Academic Center for Pathomorphological and Genetic-Molecular Diagnostics ltd, Bialystok, Poland 2. National Institute of Public Health - National Institute of Hygiene, Warsaw, Poland
Alabama Department of Public Health (ADPH)
Pathogen Discovery, Respiratory Viruses Branch, Division of Viral Diseases, CDC
Alameda County Public Health Department
Alaska State Public Health Virology Laboratory
Alaska State Virology Laboratory, 1051 Sheenjek Dr, Fairbanks, AK 99709, USA
Wadsworth Center, New York State Department.of Health
Alberta Precision Labs (APL)
Public Health Agency of Canada (PHAC) National Microbiology Laboratory
Albertsen lab, Department of Chemistry and Bioscience, Aalborg University, Denmark
Alea Genetic Centre
Alea Genetic Center
ONCOGENE LLC
KU Leuven, Rega Institute, Clinical and Epidemiological Virology
UAntwerp, Laboratory of Medical Microbiology
UAntwerp, Laboratory of Medical Microbiology,
Hong Kong Department of Health
Indian Council of Medical Research-National Institute of Virology,Microbial Containment Complex
Virology Laboratory, AIIMS Delhi
National Institute of Biomedical Genomics
National Institute of Biomedical Genomics -DBT's PAN-INDIA 1000 SARS--CoV-2 RNA Genome Sequencing Consortium
Institute of Life Sciences-INSACOG
Indian Council of Medical Research-National Institute of Virology, Microbial Containment Complex
Allergy, Immunology and Cell Biology Unit (AICBU)
Minnesota Department of Health, Public Health Laboratory
RSE National Center for Biotechnology
RSE National Center for Biotechnology and RSE National Center of Expertise
LBM ALPHABIO, Marseille
Alsafar
Alsafar - Khalifa University Abu Dhabi
IrsiCaixa
IrsiCaixa - Can Ruti CovidSeq
Altius Institute for Biomedical Research
Fred Hutchinson Cancer Research Center
RIPHL at Rush University Medical Center
Amazon Dx, AUV2 Laboratory
Crosetto lab, Karolinska Institutet, SciLifeLab
Colleen B. Jonsson
National Health Laboratory Service (NHLS), Tygerberg
EHPI lab Universit di catania
National Reference Center for Viruses of Respiratory Infections, Institut Pasteur, Paris
Institute of Medical Virology, University of Zurich
Microbiology University Politecnica delle Marche
Andersen Lab, The Scripps Research Institute
Anhui Provincial CDC, 12560, Fanhua Avenue, Anhui Province, China
Robert Koch Institute, Influenza and respiratory viruses FG17 & Bioinformatics MF1, Berlin, Germany
PPHC
ANOUAL

ANOUAL
Aomori Prefectural Public Health and Environment Center
AOP Policlinico G. Rodolico- S. Marco PO Rodolico Catania and BIOMETEC UNICT
Istituto Zooprofilattico Sperimentale delle Venezie
Laboratorio di patologia clinica e microbiologia A.O.R - San Carlo Potenza
AOU Polclinico G Rodolico-S.Marco PO Rodolico and BIOMETEC UNICT CATANIA
AOU Policlinico G. Rodolico- S. Marco PO Rodolico and BIOMETEC department UNICT
AOU Policlinico G. Rodolico- S. Marco PO G. Rodolico and Biometec UNICT
AOU Policlinico G. Rodolico-S. Marco, P.O. G. Rodolico
AOU Policlinico G. Rodolico-S. Marco, P.O. G. Rodolico and BIOMETEC Department University of Catania
CSIR-Centre for Cellular and Molecular Biology-INSACOG
CSIR-Centre for Cellular and Molecular Biology
CDFD
Applied DNA Sciences Inc
Istituto Zooprofilattico Sperimentale della Lombardia e dell'Emilia Romagna (IZSLER), Risk Analysis and Genomic Epidemiology Unit
Area Biologia Molecolare - Istituto Zooprofilattico Sperimentale della Sicilia
Area Biologia Molecolare Istituto Zooprofilattico Sperimentale della Sicilia
Inciensa, Instituto Costarricense de Investigacin y Enseanza en Nutricin y Salud
Area of Virology, Serology and Virology Division (SAViD), New South Wales Health Pathology Randwick
Microbiology RPAH
Virology Research Laboratory; Area of Virology, Serology and Virology Division (SAViD), New South Wales Health Pathology Randwick
Area of Virology, Serology and Virology Division (SAVID), New South Wales Health Pathology Randwick
ARGO Open Lab Platform for Genome sequencing
ARGO Open Lab Platform for Genome Sequencing
Instituto Nacional de Medicina Genomica
University of Minnesota Genomics Center
Plateforme de testing Namuroise
Armauer Hansen Research Institute
Ethiopian Defense Force Health Promotion and Disease Prevention Department, Ethiopian Defense Force Health Main Department; Department of Infectious Diseases, Southwest Hospital, Army Medical University
Ethiopian Defense Force Health Promotion and Disease Prevention Department, Ethiopian Defense Force Health Main Department; Department of Infectious Diseases, Southwest Hospital, Army Medical University.
National Centre For Cell Science
Army Medical and Veterinary Research Center
Army Medical Center, Scientific Department, Virology Laboratory
University Campus Bio-Medico of Rome (UCBM)
King Khalid Bin Abdul Aziz street, South Surra, Kuwait City
ARUP Laboratories
INSACOG-KA, NIMHANS
Research Institute for Tropical Medicine
University campus Bio-Medico of Rome
University Campus Bio-Medico of Rome
University Campus Bio-Medico, Rome
Univesrity Campus Bio_Medico of Rome
Associao Fundo de Incentivo Pesquisa (AFIP)
Associao Fundo de Incentivo Pesquisa (AFIP).
Associao Fundo de Incentivo a Pesquisa
ASST Grande ospedale Metropolitano Niguarda
Dep. Of Oncology and Hemato-Oncology University of Milan
ASST MONZA
National Institute of Health, Department of Medical Sciences, Ministry of Public Health, Thailand
Atlas Genomics
UW Virology Lab
Division of Genomic Medicine and Innovation support,Department of Medical Sciences, Ministry of Public Health, Thailand
Reference laboratory for the control of viral infections
Synergy Laboratories
UOSD Genetica e Citogenetica - Azienda ULSS 3 Serenissima; Istituto Zooprofilattico Sperimentale delle Venezie
South Dakota Public Health Laboratory
AUSTRAL-omics

AUSTRAL-omics, UACh
CIDM-PH, Westmead Hospital
Center for Virology
Ayass Bioscience LLC
Instituto Nacional de Salud - Direccin de Investigacin en Salud Pblica
AZ Delta
Imeda Hospital
Imelda
Imelda Hospital
Imelda Ziekenhuis
AZ Klina
AZ KLina
AZ Sint Jan
AZ Sint-Lucas Gent
Imelda hospital Bonheiden
TGen North
AZ St.Jan Brugge-Oostende
Azarian Lab at Burnett School of Biomedical Sciences
AZ Delta Medical Laboratories in Roeselare, Belgium
AZDelta
Azienda Ospedaliera Pugliese Ciaccio di Catanzaro SOC Microbiologia e Virologia
University Campus Bio-Medico of Rome (UCBM
Istituto di Genomica Applicata; Istituto Zooprofilattico Sperimentale delle Venezie
Zooprofilattico Sperimentale dell'Emilia Romagna e della Lombardia (IZSLER), Risk Analysis and Genomic Epidemiology Unit
Azienda Sanitaria dell'Alto Adige
U.O. Microbiologia, Laboratorio Unico Centro Servizi - AUSL della Romagna
Centro Polidiagnostico strumentale AMES
U.O. Genomics, S.S. Genetics and Advanced Omics Techniques, Istituto Zooprofilattico Sperimentale del Piemonte, Liguria e Valle d'Aosta
National Public Health Laboratory/CSIR-Institute of Genomics and Integrative Biology
Gujarat Biotechnology Research Centre
Bacteriology, Georgia Public Health Laboratory (GPHL)
Biosafety Level-3 Laboratory, Indonesian Institute of Sciences (LIPI)
virology lab
Laboratory of Molecular Biology and Cancer Immunology,Lebanese University Public Health England
Guangdong Provincial Center for Diseases Control and prevention
Institute for Infectious Diseases
Institute for Infectious Diseases, University of Bern
Lithuanian University of Health Sciences Hospital, Department of Genetics and Molecular Medicine
National Institute of Health. Department of medical Sciences, Ministry of Public Health, Thailand
Molecular HIV and Emerging Infectious Diseases Laboratory
ACME Lab, Oswaldo Cruz Foundation, FIOCRUZ/CE
inStem NCBS INSACOG
Department of Neurovirology, National Institute of Mental Health and Neurosciences (NIMHANS)
Barts Health NHS Trust
Barts Health NHS TRust
COVID-19 Genomics UK (COG-UK) Consortium
Wellcome Sanger Institute for the COVID-19 Genomics UK Consortium
Basic and Applied Research on Jute Project
Biocruces
USAMRD-A, Basic Science Laboratory
Salemi Lab, University of Florida
National Reference Laboratory, Nigeria Centre for Disease Control
Bayerisches Landesamt fr Gesundheit und Lebensmittelsicherheit (LGL)
Baylor College of Medicine: HGSC
Baylor College of Medicine/ GCID
Baylor Esoteric + Molecular Lab
Baylor Esoteric and Molecular Lab
Baylor Scott & White-Temple

Baylor Scott & White - Temple
Baylor Scott & White Medical Center - Temple
Genetics Working Group (Pokja Genetik) Faculty of Medicine, Public Health and Nursing Universitas Gadjah Mada (FK-KMK UGM); Disease Investigation Center Wates Ministry of Agriculture Indonesia; Department of Microbiology FK-KMK UGM; Laboratorium Diagnostik Yayasan Tahija World Mosquito Program (WMP) Yogyakarta Center for Tropical Medicine FK-KMK UGM; Integrated Research Center FK-KMK UGM; Department of Computer Science and Electronics FMIPA UGM; RSUP Dr. Sardjito
Genesupport
BCCDC Public Health Laboratory
BCCDC Public Health Laboratory
BIOBANCO / COCTI
Delaware Public Health Lab
Access DX Laboratory
Beijing Center for Disease Prevention and Control
Beijing Ditan Hospital, Capital Medical University
Beijing Institute of Microbiology and Epidemiology
New York City Public Health Laboratory
Laboratrio de Virologia Clnica e Molecular
Respiratory Virus Unit, National Infection Service, Public Health England
Department of Virology, Henri Mondor University Hospital, Assistance Publique Hpitaux de Paris, Universit Paris-Est Crteil, INSERM U955
AGES, Institute for Medical Microbiology and Hygiene
Bezmialem Vakif University, Medical School & Beykoz Institute of Life Sciences & Biotechnology
BGI-shenzhen & The First Affiliated Hospital of Guangzhou Medical University
BGI PathoGenesis Pharmaceutical Technology Co., Ltd; China CDC; Shandong First Medical University & Shandong Academy of Medical Sciences; Hubei Provincial CDC
Institute of Tropical Disease, Universitas Airlangga; Faculty of Medicine, Universitas Sumatera Utara
Wojewodzka Stacja Sanitarno-Epidemiologiczna w Lodzi, Oddzial Laboratoryjny Mikrobiologii i Parazytologii
WSSE w Warszawie
WSSE Warszawa
WSSE_Gorzow
iPROMISE, UiTM
Expert Microbiology, National Institute for Health and Welfare
Department of Neurovirology, National Institute of Mental Health and Neuroscience (NIMHANS)
Institute of Tropical Disease, Universitas Airlangga; Fakultas Kedokteran, Universitas Sumatra Utara
Institute of Health and Community Medicine
Laboratoire de virologie, CNR arbovirus Associ, Chu de la Runion
Bio Info Experts, Bio Info Experts, 718 Bayou Lane, Thibodaux, LA 70301, USA
CHU Poitiers
Clinical Bacteriology
Biochemistry and Molecular Biology Department-Faculty of Medicine, Al-Quds University
Biochemistry and Molecular Biology, Israel Institute for Biological Research, Reuven 24, Ness Ziona 74100, Israel
GenBio
Bioinfo, Vision Medicals
Bioinfoexperts, LLC
BioInfoExperts, LLC
BioInfoExperts, LLC, 718 Bayou Lane, Thibodaux, LA 70301, USA
Bioinformatics and Genomics Department, Naval Medical Research Center Biological Defense Research Directorate, 8400 Research Plaza, Frederick, MD 21702, USA
Bioinformatics Laboratory
Bioinformatics Laboratory - LNCC
Bioinformatics Laboratory / LNCC
Bioinformatics Research Group, Szentgothai Research Centre
Bioinformatics Research Group, Szentgothai Research Centre, University of Pcs
Princess Haya Biotechnology Center, Jordan University of Science and Technology
Princess Haya Biotechnology Center/ Jordan University of Science & Technology
Biologia molecular de enfermedades emergentes y EPOC, Instituto Nacional de Enfermedades Respiratorias
Biological prevention, army
Department of Virology, Faculty of Medicine, University of Helsinki, Helsinki, Finland generated and submitted to GISAID
Microbiology and Infections Diseases
Microbiology and Infectious Diseases Dpt
Biology, College of Education

Biology, MCL
Biology, UFLA
Swiss Tropical and Public Health Institute
Biomdicos de Mrida
Biomedical Laboratory 2
Biomedical Laboratory 2,
Biomedical Research Center (BRC)
Biomedical Science and Technology, Konkuk University, Laboratory of Cytokine Immunology (Room 601-1) IBST,Konkuk University
Microbiologia Molecular, Instituto SELADIS, Universidad Mayor de San Andrs
Molecular Genetics Laboratory, Instituto de Investigaciones Qumicas, Universidad Mayor de San Andrs
Laboratoire national de sante, Microbiology, Microbial Genomics Platform
Bioptick laborato s.r.o.
Institute of Applied Biotechnologies a.s.
Biopticka Laboratory
Latvian Biomedical Research and Study Centre
NJ Public Health and Environmental Laboratories
Northwestern University - Center for Pathogen Genomics and Microbial Evolution
Ozer Lab
Africa Centre for Excellence for Genomics of Infectious Diseases (ACEGID), Redeemers University
National Research and Innovation Agency (BRIN-Indonesia)
Biotechnology, Center for Advanced Technologies, Talabalar kuchasi 3A, Tashkent 100000, Uzbekistan
Biotechnology, National Centre for Disease Control
Biotia
Child Health Research Foundation
Virology Laboratory, International Centre for Diarrhoeal Disease Research, Bangladesh (ICDDR,B)
IISER Pune
Institute of Virology Department of Hygiene, Microbiology and Public Health at Innsbruck Medical University
Eijkman Institute for Molecular Biology, National Research and Innovation Agency
The Institute of Molecular Biology and Genetics of NASU
Boise State University Genetics and Infectious Disease Laboratory
Genetics Research Center, University of Social Welfare and Rehabilitation Sciences
Boston Universit/National Emerging Infectious Disease Laboratories
Boston University/National Emerging Infectious Disease Laboratories
Botswana Harvard AIDS Institute Partnership
Botswana Harvard HIV Reference Laboratory
Wiedenheft lab, Montana State University
Wiendenheft lab, Montana State University
Central Biological Research Laboratory and Department of Biochemistry and Molecular Biology Central Biological Research Laboratory and Department of Biochemistry and Molecular Biology
Breuer Lab, UCL
Jonathan Li laboratory
Infectious Disease Program, Broad Institute of Harvard and MIT
Brotman Baty Institute for Precision Medicine
HEGP - Laboratoire de Virologie
BSL-3 Lab, National Institute for Viral Disease Control and Prevention, Chinese CDC
Department of Medicinal Genetics, Bursa Uluda University, Faculty of medicine By Sehime Glsn Temel, Adem Alemdar, Kadir Yeilba
Genomic Laboratory (GLAB), Istanbul Technical University
BSWMC-Temple Molecular
BTC, Khalifa University
Indonesian Institute of Sciences (LIPI)/National Agency of Research and Innovation (BRIN) and National Institute of Health Research and Development (NIHRD)
National Research and Innovation Agency (BRIN-Indonesia, VenomCoV Project Team); National Institute of Health Research and Development (NIHRD)
Eijkman Institute for Molecular Biology, National Research and Innovation Agency; National Institute of Health Research and Development
Eijkman Institute for Molecular Biology, Ministry of Research and Technology/National Agency for Research and Innovation; National Institute of Health Research and Development
Genomik Solidaritas Indonesia Laboratorium

Bureau of Laboratories
Bureau of Laboratories, Michigan Department of Health and Human Services, 3350 N Martin Luther King Jr Blvd, Lansing, MI 48906, USA
Instituto de Salud Carlos III

Centre Hospitalier Universitaire Dijon Laboratoire de Virologie Plateforme de Biologie Hospitalo-Universitaire
Cadham Provincial laboratory
National Microbiology Laboratory (NML)
National Microbiology Laboratory
California Department of Public Health
Chiu Laboratory, University of California, San Francisco
University of California, San Francisco
Los Angeles County Public Health Laboratories
SON ESPASES UNIVERSITARY HOSPITAL
Cantacuzino Institute
Cantacuzino Institute Virology
Clinical Center, University of Sarajevo; Unit for Clinical Microbiology
Banc de Sang i Teixits
CardioVir EA-4684, University of Reims Champagne Ardenne
Carrington Lab, Department of PreClinical Sciences
Carrington Lab, Department of PreClinical Sciences, Building 36, First Floor Biochemistry Unit, Faculty of Medical Sciences, The University of the West Indies
Carrington Lab, Department of PreClinical Sciences, Faculty of Medical Sciences, The University of the West Indies
Carrington Lab, Department ofBuilding 36, First Floor Biochemistry Unit, Faculty of Medical Sciences, The University of the West Indies
CAS Key Laboratory of Pathogenic Microbiology and Immunology, Institute of Microbiology, Chinese Academy of Sciences
CAS Key Laboratory of Special Pathogens and Biosafety and Center for Emerging Infectious Diseases, Wuhan Institute of Virology, Chinese Academy of Sciences
Oswaldo Cruz Institute, FIOCRUZ/CE
COVID LAB
Department of InspectionCDC of Lishui
CDC, Dengue Branch
Instituto de Medicina Tropical de Sao Paulo
CHU Clermont-Ferrand, service de virologie
CSIR-CDRI/SGPGI
Cedars-Sinai Medical Center, Molecular Pathology Laboratory of Department of Pathology & Laboratory Medicine and Genomic Core
CHU Purpan - Laboratoire de Virologie - Institut Fdratif de Biologie
CEIRS Data Processing and Coordinating Center, St. Jude Center of Excellence for Influenza Research and Surveillance (CEIRS)
CEIRS Data Processing and Coordinating Center, Center for Research on Influenza Pathogenesis (CRIP)
CEIRS Data Processing and Coordinating Center, St. Jude Center of Excellence for Influenza Research and Surveillance (CEIRS)
St. Jude Children's Research Hospital
1. Tricity SARS-CoV-2 sequencing consortium: University of Gdansk, Medical University of Gdansk, Vaxican Ltd., Invicta Ltd. 2. National Institute of Public Health - National Institute of Hygiene, Warsaw, Poland
Functional Genomic Platform UATRS-biology, CNRST
Wojewodzka Stacja Sanitarno-Epidemiologiczna w Rzeszowie, Laboratorium Diagnostyki Medycznej
Center for Biotechnology and Cell Therapy, So Rafael Hospital, Salvador, Brazil
Central Public Health Laboratory - LACEN -Bahia, Salvador, Brazil
Center for Devices and Radiological Health, US Food and Drug Administration, 10903 New Hampshire Avenue, Silver Spring, MD 20993-0002, USA
Center for Diagnostics, Institute of Medical Microbiology, Virology and Hygiene, Martinistr. 52, Hamburg 20251, Germany
Center for Disease prevetion and Control of PLA
Center for Mathematical Modeling and Center for Genome Regulation. Santiago, Chile
Center for Genome Research and Biocomputing
Center for Genome Sciences, USAMRIID
Center for Genome Sciences, US Army Medical Research Institute of Infectious Disease (USAMRIID)
Center for Laboratory Control of Infectious Diseases, Korea Centers for Diseases Control and Prevention
Center for Laboratory Medicine St. Gallen
Department of Microbiology, Faculty of Medicine, University of Yamanashi
CDC Atlanta
Center for Personalized Medicine, Children's Hospital Los Angeles
Research Center for Genetic Engineering and Biotechnology Georgi D. Efremov , Macedonian Academy of Sciences and Arts
Center for Research and Innovation, Faculty of Medical Technology, Mahidol University
Institute of Microbiology, Universidad San Francisco de Quito
Center for Vaccine Research, University of Pittsburgh School of Medicine
Center of Advanced Studies and Technology, CAST
Center of Advanced Studies and Technology, Molecular Genetics Laboratory
Center of Excellence in Clinical Virology

Research Unit of Systems Microbiology
Center of Excellence in Clinical Virology, Faculty of Medical, Chulalongkorn University, 1873 Pathumwan Road, Bangkok, Bangkok 10330, Thailand
Center of Medical Microbiology, Virology, and Hospital Hygiene
Center of Medical Microbiology, Virology, and Hospital Hygiene, University of Duesseldorf
Center of Medical Microbiology, Virology, and Hospital Hygiene, University of Duesseldorf
Center of Scientific Excellence for Influenza Viruses, National Research Centre (NRC), Egypt.
Center of Scientific Excellence for Influenza Viruses,National Research Centre (NRC), Egypt.
Centers for Disease Control (Taiwan)
Centers for Disease Control, R.O.C. (Taiwan)
Centogene
Centogene AG
Unidad Universitaria de Secuenciacion Masiva y Bioinformatica
Central Health Laboratory ,Victoria Hospital, Candos,Ministry of Health and Wellness, Mauritius
Central laboratory
Laboratory of Respiratory Viruses and Measles, Oswaldo Cruz Institute, FIOCRUZ
State Center for Health Surveillance of the Health Department of the State of Rio Grande do Sul(CEVS_SES-RS)
Central laboratory, Infectious disease hospital, China, Guangrui road 1215, Wuxi, Jiangsu 214016, China
Next Generation Sequencing Reference Laboratory, Faculty of Medicine, CAIRO UNIVERSITY and The Center for Genome and Microbiome Research, Faculty of Pharmacy, CAIRO UNIVERSITY
Next Generation Sequencing Reference Laboratory, Faculty of Medicine, Cairo University and The Center for Genome and Microbiome Research, Faculty of Pharmacy, CAIRO UNIVERSITY
Central Public Health Lab, National Public Health Organization
National Public Health Laboratory, National Centre for Infectious Diseases
Laboratory of Genomics and Bioinformatics of the Forest Research Institute of the NAS of Belarus
Central Virology Laboratory
Central Virology Laboratory, Israel Ministry of Health
Israel Institute for Biological Research
Central Virology Laboratory, Ministry of Health
TransVIHMI, IRD/INSERM/Monpellier University
Department of Emerging Infectious Diseases, Institute of Tropical Medicine, Nagasaki University
Functional Genomic Platform/Service Analyses Biologique/UATRS/ Centre National Pour la Recherche Scientifique Et Technique (CNRST)
THE AFRICA GENOMICS CENTRE AND CONSULTANCY
THE AFRICA GENOMICS CENTRE AND CONSULTANCY LIMITED
Respiratory Virus Unit, Microbiology Services Colindale, Public Health England
Centre for Clinical Infection and Diagnostics Research and Genomics Innovation Unit, Guy's and St. Thomas' NHS Trust
Centre for Dengue Research
Centre for Dengue Research, USJ, SL
Centre for Dengue Research, Department of Immunology and Molecular Medicine
Centre for Dengue Research, Department of Immunology and Molecular Medicine,
Centre for DNA Fingerprinting and Diagnostics
Central Research Laboratory, Nigerian Institute of Medical Research
Centre for Human Virology and Genomics, Microbiology Department, Nigerian Institute of Medical Research
Centre for Infectious Diseases, CSIR-NEIST
Centre Hospitalier Universitaire de Rouen Laboratoire de Virologie
Institut Pasteur de la Guyane
Institut de Pathologie et Gntique (IPG)
Centre Interdisciplinaire de Recherches Mdicales de Franceville
Project group Epidemology of Highly Pathogenic Microorganisms, Robert Koch Institut
Institut Pasteur de Dakar
Virology Service, Centre Pasteur of Cameroun
Riga East University Hospital, National Microbiology Reference Laboratory; Eurofins Genomics Europe Sequencing GmbH
Riga East University Hospital-National Microbiology Reference Laboratory; Eurofins Genomics Europe Sequencing GmbH
Centro de Desenvolvimento Tecnolgico em Sade - CDTS
Epiclin
Laboratorio de Biologa Molecular, Instituto de Medicina Regional on behalf of 'Proyecto Argentino Interinstitucional de genomica de SARS-CoV-2' (PAIS Consortium)
Centro de Investigacin Biomdica de La Rioja
Instituto de Biotecnologa de la UNAM
Instituto Nacional de Enfermedades Respiratorias (INER): Centro de Investigacin en Enfermedades Infecciosas (CIENI)
Unidad de Genomica Avanzada

Centro de Investigacin en Ciencias de la Salud y Biomedicina, U.A.S.L.P

Centro de Investigacin en Ciencias de la Salud y Biomedicina, U.A.S.L.P.

Centro de Investigacin en Ciencias de la Salud y Biomedicina, U.A.S.LP.

CINVESTAV

rea de Secuenciacin del Laboratorio de Virologa del Hospital de Nios Dr. Ricardo Gutierrez on behalf of 'Proyecto Argentino Interinstitucional de genomica de SARS-CoV-2' (PAIS Consortium)

Centro de Investigaciones en Microbiologa y Biotecnologa-UR (CIMBIUR), Facultad de Ciencias Naturales, Universidad del Rosario, Bogot, Colombia Instituto Nacional de Salud, Bogot, Colombia Icahn School of Medicine at Mount Sinai, New York, USA

Instituto de Biomedicina de Valncia - CSIC

Laboratorio Central Mg. Luis Alfredo Pianciola

Laboratorio Mixto de Biotecnologa Acutica (LMBA) on behalf of 'Proyecto Argentino Interinstitucional de genomica de SARS-CoV-2' (PAIS Consortium)

Laboratorio Mixto de Biotecnologa Acutica (LMBA), on behalf of 'Proyecto Argentino Interinstitucional de genomica de SARS-CoV-2' (PAIS Consortium)

Institute of Biomedicine (IBiMED), Universidade de Aveiro

Laboratorio de salud pblica (Bogot) and Gencore (Universidad de los Andes)

CENTRO MEDICO NAVAL SEMAR LAB PATOLOGIA MOLECULAR

CENTRO MEDICO NAVAL SEMAR LABORATORIO DE PATOLOGIA MOLECULAR

CENTRO MEDICO NAVAL SEMAR LABORATORIO DE PATOLOGA MOLECULAR

Instituto Butantan / ESALQ-USP

Diagtron Laboratoria

Institut Pasteur de Montevideo

CERBA LAB

Cerba

Cerba Lab

Cerba LAb

CHU Lille - Laboratoire de Virologie

CHU Nantes Virology

Institut Pasteur

Charit Universittsmedizin Berlin, Institute of Virology, Charitplatz 1, 10117 Berlin, Germany

Charite Universitaetsmedizin Berlin, Institute of Virology

Charite Universitatsmedizin Berlin, Institut fur Virologie, Charitplatz 1, 10117 Berlin, Germany

Charite Universitatsmedizin Berlin, Institute of Virology

HLAGYN - Laboratorio de Imunologia de Transplantes de Goias

Division of Virology and Zoology, Chiba Prefectural Institute of Public Health

Child Health Research Lab

van Bakel Laboratory, Genetics and Genomics Sciences, Icahn School of Medicine at Mount Sinai

Children's National Hospital

China CDC; Shandong First Medical University & Shandong Academy of Medical Sciences; Hubei Provincial CDC; BGI PathoGenesis Pharmaceutical Technology Co., Ltd

Chinese CDC

Chiu Laboratory UCSF-Abbott Viral Diagnostics and Discovery Center

Chiu Laboratory UCSF-Abbott Viral Diagnostics and Discovery Center University of California, San Francisco

Chiu Laboratory, UCSF-Abbott Viral Diagnostics and Discovery Center, University of California, San Francisco

Chiu Laboratory, UCSF-Abbott Viral Diagnostics and Discovery Center, University of California, San Francisco

Chongqing Municipal CDC, Chongqing, China

CHU LILLE

CHU NIMES

CHU Pontchaillou

Laboratoire de virologie - cole Nationale Vtrinaire de Toulouse

IBP-laboratoire de virologie

CIAD Hermosillo

Cibic Laboratorios on behalf of 'Proyecto Argentino Interinstitucional de genomica de SARS-CoV-2' (PAIS Consortium)

Cicin-Sain Lab

Malaria Research and Training Center (MRTC-Parasito)

Bundeswehr Institut of Microbiology

Malaria Research and Training Center-Bamako

Malaria Research and Training Center-Parasito

Instituto de Diagnostico y Referencia Epidemiologicos (INDRE)

City of Milwaukee Health Department Laboratory

CSIR-Institute of Microbial Technology

Stern Lab

Laboratory of virology and molecular diagnostics, Institute of Public Health

Instituto Nacional de Salud

Molecular Genetics and Antimicrobial Resistance - UGRA, Universidad El Bosque

Instituto Nacional de Salud- Direccin de Investigacin en Salud Pblica, Universidad de los Andes- Applied genomics research group, Vicerrectoria de Investigacin y Creacin, Universidad de los Andes- Systems and Computing Engineering Department

Facultad de Ciencias de la Vida, UNAB

Telethon Institute of Genetics and Medicine (TIGEM)

Corporacion CorpoGen-Universidad de los Andes-Universidad Central

Clinical Diagnostics Laboratory, Diagnostic & Experimental Pathology, Lilly Research Laboratories

Clinical Diagnostics Laboratory, Diagnostic & Experimental Pathology, Lilly Research Laboratories

Clinical Division, Fred Hutchinson Cancer Research Center

Croatian Institute of Public Health

Infectious Diseases Research Laboratory, Department of Pediatrics and Child Health

Department of Clinical Laboratory Medicine, Kyoto University Graduate School of Medicine

IDF medical corps

Infectious Disease Research Department, King Abdullah International Medical Research Center (KAIMRC)

Clinical Microbiology Lab RSPTN Universitas Hasanuddin

Infectious Diseases Research, King Abdullah International Medical Research Center (KAIMRC), Riyadh, Saudi Arabia

Clinical microbiology lab, RSPTN Universitas Hasanuddin

James Molecular Laboratory

Biocruces-Bizkaia

BioCruces Bizkaia

Clinical Microbiology Laboratory, Faculty of Medicine, Universitas Indonesia

Faculty of Medicine, Universitas Indonesia

ClickSeq Lab, University of Texas Medical Branch, UTMB, Galveston

Dirk Dittmer

Jeremy Wang

National Institute for Infectious Diseases, INMI, L. Spallanzani IRCCS

Centre de Recherche et de Formation en Infectiologie Guine

Enteric Viruses Group, ICMR-National Institute of Virology

Center for Personalized Medicine, Children's Hospital Los Angeles

ILBS

ILBS - IGIB

Laboratorio de Genmica Microbiana, Unidad de Bioqumica, Departamento Biomdico, Facultad de Ciencias de la Salud, Universidad de Antofagasta, Chile

Clnica Rotger Laboratorio de Anlisis Clnicos Dpto. Biologa Molecular

CMBG FN Brno

Laboratorio Central Mg. Luis Alfredo Plancíola on behalf of "Proyecto Argentino Interinstitucional de genómica de SARS-CoV-2" (PAIS Consortium)

Synergy Laboratories, Inc.

N.F. Gamaleya Research Center for Epidemiology and Microbiology

Center for Precision Genome Editing and Genetic Technologies for Biomedicine, Pirogov Medical University Moscow Russian Federation

Laboratory of genome analysis, N.F. Gamaleya National Research Center for Epidemiology and Microbiology of the Ministry of Health of the Russian Federation

Colorado Department of Public Health & Environment

Colorado Department of Public Health and Environment

Colorado Department of Puplic Health and Environment

Colorado State University - Ebel Lab

Communicable Disease Laboratory, Public Health Directorate

Communicable Diseases, Interactive Research School for Health Affairs (IRSHA)

Communicable Diseases, Interactive Research School for Health Affairs

Compass Laboratory Services

Centro de Investigaciones en Microbiologa y Biotecnologa-UR (CIMBIUR), Facultad de Ciencias Naturales, Universidad del Rosario, Bogot, Colombia

Microbiology Department. Complexo Hospitalario Universitario de Vigo

Yale Center for Genomic Analysis

Contra Costa Public Health Laboratory

TIGEM

Institute of Medical Microbiology and Virology, University Hospital Carl Gustav Carus, TU Dresden

County of San Luis Obispo Public Health Laboratory

Genomic Research Lab, Bangladesh Council of Scientific and Industrial Research

Covid-19 laboratory, Institute of Human Genetics, Polish Academy of Sciences

COVID-19 National Reference Laboratory
COVID-19 Network Investigations (CONI) Alliance
Cayman Islands Forensic Lab
Cayman Islands Forensic Science Lab
Health Services Authority KY1-1103
Covid lab center, Yaftabad Hospital, Southern Al-Ghadir Blvd
Department of Pathology, Bangladesh Agricultural University and Department of Microbiology, Mymensingh Medical College
Department of Pathology, Bangladesh Agricultural University and Department of Microbiology, Mymensingh Medical College,
Department of Pathology, Bangladesh Agricultural University Department of Microbiology, Mymensingh Medical College
Molecular Hepatology AOU Messina
Molecular Hepatology Lab AOU Messina
IEDCR-ideSHi Genomics Lab
CPHL, MOH, EGYPT
CPHL
CPHL/MOH/EGYPT
Tropical Infectious Diseases Research & Education Centre (TIDREC), Universiti Malaya
Creighton COVID Consortium
TransVIHMI(Recherches Translationnelles sur le VIH et les Maladies Infectieuses)
University of Zagreb, Centre for research and knowledge transfer in biotechnology
CSIR-CDRI/SGPGI, Lucknow
CSIR-CDRI, Lucknow
CSIr-CDRI/SGPGI, Lucknow
CSIR-CDRi/SGPGI, Lucknow
CSIR-IGIB
CSIR-Indian Institute of Chemical Biology, MEDICA Supercpecialty Hospital Kolkata
CSIR Institute of Himalayan Bioresource Technology
CSL, Maryland Department of Health
CT Department of Public Health
CTMR, Karolinska Institutet, Stockholm, Sweden
CTvacinas
CUB Hopital Erasme Laboratoire d'Anatomie Pathologique
Creighton University School of Medicine, Departments of Medical Microbiology and Pharmacology and Neuroscience
Curative Inc
Curative Labs
University Hospital Brno, CMBG
DASA
Dasman diabetes Institute
Dasman Diabetes Institute
Data Science, Chan-Zuckerberg Biohub, 499 Illinois St, San Francisco, CA 94158, USA
Laboratrio de Parasitologia Mdica - Instituto de Medicina Tropical - Universidade de So Paulo
Instituto de Medicina Tropical da Univesidade de So Paulo
DC Public Health Lab/ Dept. of Forensic Sciences
deCODE genetics
Defence Research & Development Establishment
Defence Research & Development Establishment (DRDE)
Defence Services Medical Research Center, Biological Research Laboratory
Delaware Public Health Laboratory
rea de Secuenciacin del Laboratorio de Virologa del Hospital de Nios Dr. Ricardo Gutierrez
Laboratorio de Secuenciacin-Centro de Instrumentos, IMSS Mxico /Unidad de Investigacin Mdica en Inmunoqumica, UMAE Hospital de Especialidades Bernardo Seplveda Gutirrez, Centro Mdico Nacional Siglo XXI, Instituto Mexicano del Seguro Social (IMSS), Mxico.
Department for Virology, Molecular Biology and Genome Research, R. G. Lugar Center for Public Health Research, National Center for Disease Control and Public Health (NCDC) of Georgia.
Institute of Molecular Medicine, Section for Molecular Cell Biology
Department of Acute Infectious Diseases Control and Prevention, Yunnan Provincial CDC
Department of Acute Infectious Diseases Control and Prevention,Yunnan Provincial CDC
Statens Serum Institut Bioinformatics and Microbial Genomics
Department of Biochemistry, Cell and Molecular Biology
WACCBIP, University of Ghana
Department of Biochemistry, Cell and Molecular Biology, West African Centre for Cell Biology of Infectious Pathogens (WACCBIP), University of Ghana

Department of Biology, University of Basrah
Department of Biomedical Sciences for Health Department of Biomedical, Surgical and Dental Sciences University of Milano
Department of Biomedical, Surgical and Dental Sciences and Istituto Clinico Citt Studi, University of Milan
Department of Clinical Diagnostics, Macrogen
Department of Clinical Laboratory, the First People's Hospital of Yunnan Province
Department of Clinical Laboratory, Tongji Hospital, Tongji Medical College, Huazhong University of Science and Technology
Switzerland
Department of Epidemiology, Infectious Disease Control and Prevention, Hiroshima University, Japan
Department of Infectious, Tropical Diseases and Immune Deficiency, Pomeranian Medical University in Szczecin, Szczecin, Poland
Department of General Services Division of Consolidated Laboratory Services, Virginia Division of Consolidated Laboratory Services Sequencing Submission Group
Department of General Services, Division of Consolidated Laboratory Services, 600 N 5th Street, Richmond, VA 23219, USA
Department of Microbiology, University Hospital Motol
Laboratory of Genomics and Bioinformatics, Comenius University Science Park
Gifu Prefectural Research Institute for Health and Environmental Sciences
Iwate Prefectural Institute of Public Health and Environment
Department of Health Technology and Informatics, Faculty of Health and Social Science, The Hong Kong Polytechnic University
Department of Health Technology and Informatics, Faculty of Health and Social Science, The Hong Kong Polytechnic University
Department of Health Technology and Informatics, Hong Kong Polytechnic University of Hong Kong
Department of Health Technology and Informatics, The Hong Kong Polytechnic University
Department of Health, Utah Public Health Laboratory, 4431 2700 W, SLC, UT 84129, USA
Department of Immunology, The Scripps Research Institute
Department of Immunology, The Scripps Research Institute, 10550 N Torrey Pines Rd, La Jolla, CA 92037-1000, USA
Department of Infection, Immunity and Cardiovascular Disease, The Florey Institute, The Medical School, University of Sheffield
Department of Infection, Immunity and Cardiovascular Disease, The Florey Institute, The Medical School, University of Sheffield
NBACC
Clinical Research Center, National Hospital Organization Nagoya Medical Center
Istituto Superiore di Sanit (ISS)
Department of Infectious Diseases, Istituto Superiore di Sanit
Center for Medical Genetics, Keio University School of Medicine, Tokyo, Japan
Genome Information Research Center, Research Institute for Microbial Diseases, Osaka University
Department of Internal Medicine, College of Medicine, Chosun University
Microbiological Analysis Team, Biometrology Group, Division of Chemical and Biological Metrology, Korea Research Institute of Standards and Science (KRISS)
Department of Laboratory Medicine and Molecular Diagnostics, Sunnybrook Health Sciences Centre
Department of Laboratory Medicine Tan Tock Seng Hospital
Laboratory of Parasitic Diseases, Systems Genomics Section, National Institute of Allergy and Infectious Diseases, National Institutes of Health
Department of Laboratory Medicine, Lin-Kou Chang Gung Memorial Hospital, Taoyuan, Taiwan
Microbial Genomics Core Lab, National Taiwan University Centers of Genomic and Precision Medicine
Department of Laboratory Medicine, Tan Tock Seng Hospital
Laboratory of Infectious Diseases, Department of Biomedical and Clinical Sciences L. Sacco, University of Milan
Department of Medical Microbiology
Department of Medical Microbiology & Infection prevention, Amsterdam University Medical Centers location AMC
DEPARTMENT OF MEDICAL MICROBIOLOGY & PARASITOLOGY, SCHOOL OF MEDICAL SCIENCES
Molecular Research Laboratory
Department of Medical Microbiology, Faculty of Medicine, University of Malaya
Department of Medical Microbiology, Faculty of Medicine, University of Malaya
Department of Medical Microbiology, Hospital Pengajar Universiti Putra Malaysia
Malaysia Genome Institute
DMR
DMR_Myanmar
Department of Medical Sciences, Ministry of Public Health, Thailand; Thai Red Cross Emerging Infectious Diseases - Health Science Centre; Department of Disease Control, Ministry of Public Health, Thailand
Department of Medical, Biotechnologies University of Siena
Department of Biomedical, Surgical and Dental Sciences; University of Milan
Department of Medicine, Democritus University of Thrace, Alexandroupolis, 68100, Department of Medicine, National and Kapodistrian University of Athens, University Campus, Athens 11527, Greece
Department of Medicine, Democritus University of Thrace, University Campus, Alexandroupolis 68100, Greece
Department of Microbial Biotechnology, Genetic Engineering Division, National Research Centre
Department of Microbiology
Department of Microbiology and immunology, McGill University Health Centre

Department of Microbiology and Immunology, The University of Melbourne at The Peter Doherty Institute for Infection and Immunity
Department of microbiology laboratory,Anhui Provincial CDC
Department of Microbiology, Mahidol University
Department of Microbiology, College of Medicine and Medical Research Institute Chungbuk National University
Department of Microbiology, Erciyes University, Yeni Dogan District, Turhan Baytop Street No:1, Kayseri 38280, Turkey
Department of Microbiology, Faculty of Medicine, Chinese University of Hong Kong, Hong Kong SAR, China
Department of Microbiology, Gandhi Medical College and Hospital, Secendrabad, Hyderabad
Department of Microbiology, Gandhi Medical College and Hospital, Secendrabad, Hyderabad, India
Department of Microbiology, Guangdong Provincial CDC
MSHS Pathogen Surveillance Program
Department of Microbiology, Institute for Viral Diseases, College of Medicine, Korea University
Department of Microbiology, PathWest QEII Medical Centre
Department of Microbiology, PathWest QEII Medical Centre
Department of Microbiology, The University of Hong Kong
The University of Hong Kong Department of Microbiology
Department of Microbiology, The University of Hong Kong, 21/F, Block T, Queen Mary Hospital, 102 Pokfulam Road, Pokfulam, Hong Kong 518000, China
Genome Analysis Center, Kamma Memorial Hospital
Department of Microbiology, University Hospital in Pilsen
Plzen Region
Genome Analysis Center, Yamanashi Central Hospital
Department of Microbiology, Yokohama City University School of Medicine
Department of Microbiology, Zhejiang Provincial CDC
Virus Research Laboratory, Department of Zoology, Osmania University, Hyderabad, India
Department of Molecular and Translational Medicine, Section of Microbiology, University of Brescia, ASST Spedali Civili, Brescia
Department of Molecular Medicine, University of Padova
Department of Molecular Virology, Cyprus Institute of Neurology and Genetics
Centre for Research in Advanced Tropical Bioscience
Centre for Research in Advanced Tropical Bioscience Universiti Malaysia Pahang, 26300 Gambang, Pahang, Malaysia
Centre for Research in Advanced Tropical Bioscience (Biotropic Centre)
Department of Pathology and Medicine, New York University School of Medicine
Department of Pathology, National Institute of Infectious Diseases
Department for Public Health Microbiology Ljubljana, National Laboratory for Health, Environment and Food
Department of Public Health Microbiology Ljubljana, National Laboratory for Health, Environment and Food
Department of Respiratory and Critical Care Medicine, Peking University People's Hospital, No. 11 Xizhimen South Street, Xicheng District, Beijing 100044, China
Department of Respiratory and other Viral Infections of L.V.Gromashevsky Institute of Epidemiology & Infectious Diseases NAMS of Ukrain, JSC Farmak
Department of Virology, Institute of Tropical Medicine, Nagasaki University, Nagasaki, Japan
Tumor Immunology Unit, Department of Health Sciences, University of Palermo School of Medicine, Section of Microbiology, University of Palermo School of Medicine; National Research Council of Italy - High Performance Computing and Networking Institute (CNR-ICAR) of Palermo
Department of Surgical Sciences, University of Cagliari - AOU, Via Ospedale 54, Cagliari, Ca 09121, Italy
Department of Surgical Sciences, University of Cagliari - AOU, Via Ospedale 54, Cagliari, Ca 09124, Italy
Department of Veterinary Biotechnalogy, College of Veterinary Science, Rajendranagar, PV Narsimha Rao Telengana Veterinary University
Center for Influenza and Respiratory Virus Research, National Institute of Infectious Diseases
Center for Influenza and Respiratory Virus Research
Department of Veterinary Science, National Institute of Infectious Diseases
Beijing Institute of Genomics, Chinese Academy of Sciences
Department of Virology, Faculty of Medicine, University of Helsinki, Helsinki, Finland
Department of Virology Faculty of Medicine, Medicum University of Helsinki
Department of Virology, Biomedical Primate Research Centre
Department of Virology, Piti-Salptrire hospital
Bioinformatics Division, National Institute of Biotechnology (NIB)
Department of Virology, Bangabandhu Sheikh Mujib Medical University
Department of Virology, Charite - Universitaetsmedizin Berlin, Chariteplatz 1, Berlin, Berlin 10117, Germany
Department of Virology, Public Health Laboratories Division
Department of Virology, Public Health Laboratories Division, National Institute of Health
Department of Virology, University of Helsinki and Helsinki University Hospital, Helsinki, Finland
Department of Virology, University of Helsinki, Haartmaninkatu 3, Helsinki 00280, Finland
Aalborg University
Albertsen Lab, Department of Chemistry and Bioscience, Aalborg University, Denmark

Department of Developmental Medicine, Research Institute,

1) Dept. Infectious, Tropical Diseases & Microbiology, IRCCS Sacro Cuore Don Calabria Hospital; 2) Centro Piattaforme Tecnologiche, University of Verona; 3) Dept. Neurosciences, Biomedicine and Movement Sciences, University of Verona.

1) Dept. Infectious, Tropical Diseases & Microbiology, IRCCS Sacro Cuore Don Calabria Hospital; 2) Dept. Biotechnology, University of Verona

Dept. of Microbiology and Infection Control, Akershus University Hospital HF

Laboratory of Histology-Embryology, Molecular Carcinogenesis Group, Faculty of Medicine, National and Kapodistrian University of Athens

Dept. of Pediatrics and Dept. Medical Microbiology, 2nd Faculty of Medicine, Charles University in Prague, V Uvalu 84, Prague, CZ 15006, Czech Republic

Dept. of Virology III, National Institute of Infectious Diseases

Dept. OPA, Beijing Institute of Microbiology and Epidemiology, 20 Dongda Street, Fengtai District, Beijing, Beijing 100071, China

DeRisi Lab, University of California, San Francisco

DNA SOLUTION LTD.

CSIR-IGIB/Max

CSIR Institute of Genomics and Integrative Biology (CSIR-IGIB) / Max

CSIR Institute of Genomics and Integrative Biology

Molecular and Genomics Research Lab, Dhulikhel Hospital, Kathmandu University Hospital

Diagen

Diagnostic- and Research Institute of Pathology, Medical University of Graz

Diagnostic and Research Center of Infectious Diseases, Medical Faculty, Andalas University

Genomik Solidaritas Indonesia Laboratorium / Diagnostic and Research Center of Infectious Diseases, Medical Faculty, Andalas University

Diagnostic Genomics Lab and Functional Genomics Core, University of South Carolina

Diagnostic Genomics Lab and Functional Genomics Core, University of South Carolina

Diagnostic Genomics Laboratory

Biomedical Laboratory-2

Armin Ensser

1. Tricity SARS-CoV-2 sequencing consortium: University of Gdansk, Medical University of Gdansk, Vaxican Ltd., Invicta Ltd. 2. National Institute of Public Health - National Institute of Hygiene, Warsaw, Poland

WSSE Gorzow

1. ViroGenetics - BSL3 Laboratory of Virology, Maopolska Centre of Biotechnology, Jagiellonian University; 2. Diagtron Laboratoria ukasz Rbalski

Corporacin para Investigaciones Biolgicas-CIB

Instituto de Medicina Tropical Universidade de So Paulo

Eijkman Institute for Molecular Biology, National Research and Innovation Agency; West Java Health Laboratory

School of Life Sciences and Technology & School of Pharmacy-Institut Teknologi Bandung; Molecular Genetics Laboratory-Faculty of Medicine-Universitas Padjadjaran; Laboratorium Kesehatan Provinsi Jawa Barat

Eijkman Institute for Molecular Biology, Ministry of Research and Technology/National Agency for Research and Innovation

Dipartimento di Medicina di Laboratorio, Azienda sanitaria universitaria Friuli Centrale (ASU FC)

Dipartimento di Medicina e Chirurgia, University of Insubria and Ospedale di Circolo e Fondazione Macchi

Istitituto Zooprofilattico Sperimentale dell'Abruzzo e Molise G.Caporale

Centro de Investigaciones Tecnolgicas, Biomdicas y Medioambientales (CITBM)

Direccion de Investigacion en Salud Publica, Instituto Nacional de Salud, Avenida calle 26 No. 51-20 - Zona 6 CAN, Bogota, Bogota 111321, Colombia

Discovery DNA

Division of Consolidated Laboratories

Division of Consolidated Laboratory Services

Steininger Lab

Department of biotechnology, Yonsei University

Department of Biotechnology, Yonsei University

Division of Infectious Diseases, Sejong Institute of Health and Environment

CERI, Centre for Epidemic Response and Innovation, Stellenbosch University and CERI-KRISP, KZN Research Innovation and Sequencing Platform

Division of Medical Virology, National Health Laboratory Service (NHLS), Tygerberg Hospital / Stellenbosch University

Division of Medical Virology, Stellenbosch University and NHLS Tygerberg Hospital

Division of Microbiology, Osaka Institute of Public Health

Division of Viral Diseases, CDC

Division of Viral Diseases, CDC, 1600 Clifton Rd NE, Atlanta, GA 30033, USA

Division of Viral Diseases, Center for Laboratory Control of Infectious Diseases, Korea Centers for Diseases Control and Prevention

Genetics Working Group (Pokja Genetik) Faculty of Medicine, Public Health and Nursing Universitas Gadjah Mada (FK-KMK UGM); Disease Investigation Center Wates Ministry of Agriculture Indonesia; Department of Microbiology FK-KMK UGM; Laboratorium Diagnostik Yayasan Tahija World Mosquito Program (WMP) Yogyakarta Center for Tropical Medicine FK-KMK UGM; Integrated Research Center FK-KMK UGM; Department of Computer Science and

Genetics Working Group (Pokja Genetik) Faculty of Medicine, Public Health and Nursing Universitas Gadjah Mada (FK-KMK UGM); Disease Investigation Center Wates Ministry of Agriculture Indonesia; Department of Microbiology FK-KMK UGM; Laboratorium Diagnostik Yayasan Tahija World Mosquito Program (WMP) Yogyakarta Center for Tropical Medicine FK-KMK UGM; Integrated Research Center FK-KMK UGM; Department of Computer Science and Electronics FMIPA UGM; RSUP Dr.

DNA Solution Ltd

DNA Solution Ltd.
DNA Solution Ltd. L-5
Laboratrio de Gentica & Biodiversidade - LGBio
LGBio - Laboratrio de Gentica & Biodiversidade
LGBio - Laboratrio de Gentica e Biodiversidade
LGBio (Laboratorio de Genetica & Biodiversidade)
Laboratoire des Procds de Criblage Molculaire et Cellulaire-Centre de Biotechnologie de Sfax
DPH, Massachusetts State Public Health Lab
DPHL
Institute of Applied Genomics
Dr. Thomas Vanderford's Lab
Dr. B. Lal Institute of Biotechnology
Stefan S. NicolauInstitute of Virology
New Jersey Public Health Environmental Laboratories (NJ_PHEL)
Dr. Gernot Walder GmbH
School of Pharmacy & School of Life Sciences and Technology - Institut Teknologi Bandung; Molecular Genetics Laboratory-Faculty of Medicine-Universitas Padjadjaran; Laboratorium Kesehatan Provinsi Jawa Barat
Microbiology, Dr. Risch
Research Center for Vaccine Technology and Development, Institute of Tropical Disease, Airlangga University
Dr. Jeff Wrana, Senior Investigator
Dr.Chiaho Shih
DSMRC
Duke Center for Genomic and Computational Biology (GCB), Duke University
University of Otago
Erasmus Medical Center Department of Virology
Medical Microbiology, Maastricht University Medical Centre
Manitoba Cadham Provincial Laboratory
McMaster University
Unidad Universitaria de Secuenciacin Masiva y Bioinformtica (UUSMB). IBT-UNAM
Ehime Prefectural Institute of Public Health and Environmental Science
Razi Vaccine and Serum Research Institute
Emergence des Maladies Virales, Centre Interdisciplinaire de Recherches Medicales de Franceville
Stefan cel Mare, University Metagenomics lab
Stefan cel Mare University Metagenomics Lab
National Institute for Viral Disease Control and Prevention
Piantadosi Lab, Emory Department of Pathology
Environmental and Global Health
Environmental and Global Health, University of Florida
Environmental and Global Health, University of Florida
Environmental and Global Health, University of Florida - Gainesville, 1225 Center Drive, Room 4155, Gainesville, FL 32610, USA
Epidemiology of Microbial Diseases, Yale School of Public Health
Yale School of Public Health
University at Buffalo Genomics and Bioinformatics Core
Etlik Veterinary Control Central Research Institute
Eurofins-NMDL
Hudsonalpha Genome Sequencing Center
Unilabs/Eskilstuna/Sweden
Evandro Chagas Institute
Evandro Chagas Institute Virology
Bielefeld University
National Reference Laboratory, Nigeria Centre for Disease Control Durumi III, Gaduwa, Abuja, Nigeria
National reference Laboratory, NCDC, Gaduwa, Abuja, Nigeria
Agiomix
Facultad de Ciencias (Seccin Gentica Evolutiva, Seccin Virologa).
Faculty of Medicine, Chulalongkorn University
Faculty of Medicine, Chulalongkorn University, Rama IV Rd, Bangkok 10330, Thailand
Faculty of Science, University of South Bohemia
Institute of Molecular Genetics CAS
Fakultn nemocnice Hradec Krlov

University Hospital Hradec Kralove
Fakultni nemocnice Ostrava
Faroese National Reference Laboratory for Fish and Animal Diseases
FBIS SRC AMB
Federal Budget Institution of Science STATE RESEARCH CENTER FOR APPLIED MICROBIOLOGY & BIOTECHNOLOGY
Hyde Lab
Fimlab Laboratories
Fimlab Laboratories, Arvo Ylpn katu 4, 33520 Tampere, Finland
UMR PIMIT Universit de La Runion
Florida Bureau of Public Health Laboratories
Florida Bureau of Public Health Laboratories, Florida Department of Health
Center of Molecular Biology and Gene Therapy (CMBG)
CMBG
Fondation Congolaise pour la Recherche Mdicale
Fondation Congolaise pour la recherche medicale (FCRM), Francine Ntoumi
NGS Competence Center Tuebingen, Institut fr Medizinische Mikrobiologie und Hygiene, Universitaetsklinikum Tbingen
Fondazione IRCCS Ca' Granda Ospedale Maggiore Policlinico
Fourth affiliated hospital, college of medicine, zhejiang university
Center for Precision Genome Editing and Genetic Technologies for Biomedicine, Pirogov Medical University, Moscow, Russian Federation
FSBSI Chumakov Federal Scientific Center for Research and Development of Immune-and-Biological Products of Russian Academy of Sciences & NRC Kurchatov institute
Fudan University
Fujian CDC
Fujita Health University School of Medicine, Department of Microbiology
Department of Virology and Parasitology, Fujita Health University School of Medicin
Department of Virology and Parasitology, Fujita Health University School of Medicine
Fujita health University, Department of Microbiology
Fukui Prefectural Institute of Public Health and Environmental Science
Fukuoka Institute of Health and Environmental Sciences
Fukushima Prefectural Institute of Public Health
Functional Genomics Core, Center For Targeted Therapeutics,
Functional Genomics Core, University of South Carolina
Functional Genomics Core, University of South Carolina,
IrsiCaixa AIDS Research Lab
Laboratorio de Biotecnologa, Universidad Icesi
Coordenao Geral de Laboratrios de Sade Pblica (CGLAB)
Coordenao Geral de Laboratrios de Sade Pblica (CGLAB/DAEVS/SVS/MS)
Fundao Ezequiel Dias
Laboratrio de Estudos de Vrus Emergentes
Maness Lab
Fuyang city CDC
FYR Diagnostics
GA Department of Public Health
UHAS COVID-19 Lab
University of Health and Allied Sciences (UHAS) COVID-19 Testing and Research Centre
Cruces University Hospital
MDRU-DHR,Gandhi Medical College
VRDL-Gandhi Medical College
Gastrointestinal and Liver Diseases Research Center, Iran University of Medical Sciences
Gastrointestinal and Liver Diseases Research Center, Iran University of Medical Sciences, Firoozgar Hospital
Gastrointestinal and Liver Diseases Research Center, Iran University of Medical Sciences, Firoozgar Hospital, Tehran, Tehran 1449614535, Iran
Gazi University Faculty of Medicine, Medical Virology Laboratory
Geelong Centre for Emerging Infectious Diseases
Laboratorio de biotecnologa, Universidad Icesi
Gencore - Universidad de los Andes
Genelabs Medical (Pvt) Ltd
124. Tricity SARS-CoV-2 sequencing consortium: University of Gdansk, Medical University of Gdansk, Vaxican Ltd., Invicta Ltd. 2. National Institute of Public Health - National Institute of Hygiene, Warsaw, Poland
125. Tricity SARS-CoV-2 sequencing consortium: University of Gdansk, Medical University of Gdansk, Vaxican Ltd., Invicta Ltd. 2. National Institute of Public Health - National Institute of Hygiene, Warsaw, Poland

126. Tricity SARS-CoV-2 sequencing consortium: University of Gdansk, Medical University of Gdansk, Vaxican Ltd., Invicta Ltd. 2. National Institute of Public Health - National Institute of Hygiene, Warsaw, Poland
127. Tricity SARS-CoV-2 sequencing consortium: University of Gdansk, Medical University of Gdansk, Vaxican Ltd., Invicta Ltd. 2. National Institute of Public Health - National Institute of Hygiene, Warsaw, Poland
128. Tricity SARS-CoV-2 sequencing consortium: University of Gdansk, Medical University of Gdansk, Vaxican Ltd., Invicta Ltd. 2. National Institute of Public Health - National Institute of Hygiene, Warsaw, Poland
129. Tricity SARS-CoV-2 sequencing consortium: University of Gdansk, Medical University of Gdansk, Vaxican Ltd., Invicta Ltd. 2. National Institute of Public Health - National Institute of Hygiene, Warsaw, Poland

Research Center for Genetic Engineering and Biotechnology Georgi D. Efremov , Macedonian Academ

Research Center for Genetic Engineering and Biotechnology Georgi D. Efremov, Macedonian Academy of Sciences and Arts

Research Center for Genetic Engineering and Biotechnology Georgi D. Efremov , Macedoni

Research Center for Genetic Engineering and Biotechnology Georgi D. Efr

Research Center for Genetic Engineering and Biotechnology Georgi D. Efremov , Macedon

Alea Genetic Center

Alela Genetic Centre

Institute of Public Health

Public Health Institute of Republic of Srpska

Pathogen Laboratory (BSL3), Biomedical Innovation Department, Experimental and Applied Biology Division, Scientific Research Center and High Education from Ensenada (CICESE)

Laboratory of Virology and Molecular Diagnostics, Institute of Public Health of Republic of North Macedonia

National Reference Laboratory for Influenza and Respiratory Viruses CZE

Genesis Institutes for Genetic Research (GIGR)

Insituto de Salud Publica de Chile

Instituto de Salud Publica de Chile

Genetica y Virologia (FC), Facultad de Ciencias & DLSP

Genolife

Genome Center

Laboratory of Bioinformatics and Computational Biology, A.C.Camargo Cancer Center

Genomic platform, APHP, 51 avenue du Marechal de Lattre Tassiny, Creteil 94000, France

Bangladesh Council of Scientific and Industrial Research

Genomic Research Laboratory, BSMMU

Genomics and Computational Biology Lab, Scientific Research Institute of Physical-Chemical Medicine, FMBA of Russia

Genomics Center, University of Minnesota

Genomics Program, Children Cancer Hospital

Genomics Program, Children Cancer Hospital

Genomics, Poplar Healthcare

genXone SA, Research & Development Laboratory

Instituto Gulbenkian de Ciencia

GHC Genetics, s.r.o.

Gibraltar Health Authority Covid-19 Laboratory

GIGA Medical Genomics

Utah Public Health Laboratory

UKM Medical Molecular Biology Institute (UMBI)

TIGSS

TxGen

Texas A&M Institute for Genomic Sciences and Society (TIGSS)

Virology Lab, Jaber Al Ahmad Hospital

Goethe University Hospital Frankfurt, Institute for Medical Virology

Gonoshasthya-RNA Molecular Diagnostic and Research Center

Gonoshasthya-RNA Molecular Research Center

Gorgas memorial Institute for Health Studies

Gorgas memorial Institute For Health Studies

Gorgas Memorial Institute for Health Studies

Gorgas Memorial Laboratory of Health Studies

LSU Health Sciences Center Emerging Viral Threat Laboratory

Kentucky State Public Health Lab

Gravity Diagnostics, LLC

G42 Healthcare

Group for molecular genetics of pathogens

Group of Genetic Engeneering and Biotechnology, Federal Budget Institution of Science 'Central Research Institute of Epidemiology' of The Federal Service on Customers' Rights Protection and Human Well-being Surveillance

Group of Genetic Engineering and Biotechnology, Federal Budget Institution of Science 'Central Research Institute of Epidemiology' of The Federal Service on Customers' Rights Protection and Human Well-being Surveillance

Grubaugh Lab - Yale School of Public Health

Grupo de Genmica y Bioinformtica del Instituto de Investigacin de la Cadena Lctea CONICET-INTA on behalf of 'Proyecto Argentino Interinstitucional de genomica de SARS-CoV-2' (PAIS Consortium)

Grupo de Investigaciones Microbiolgicas-UR (GIMUR), Departamento de Biologa, Facultad de Ciencias Naturales, Universidad del Rosario, Bogot, Colombia Instituto Nacional de Salud, Bogot, Colombia Icahn School of Medicine at Mount Sinai, New York, USA

Guangdong Provincial Center for Diseases Control and Prevention

Guangdong Provincial Institution of Public Health

Guangzhou Eighth People's Hospital, Guangzhou, China

Gujarat Biotechnology Research Centre, Gandhinagar

Gujarat Biotechnology Research Centre, Sixth floor, Block B&D, MS Building, Gh Road, Sector-11, Gandhinagar, Gujarat 382010, India

Gyncentrum

UAntwerp, Laboratory of Medical Microbiology, Campus Drie Eiken S6.26, Universiteitsplein 1, 2610, Wilrijk, Antwerp, Belgium

UAntwerp, Laboratory of Medical Microbiology, Campus Drie Eiken S6.26, Universiteitsplein 1, 2610, Wilrijk, Belgium

Laboratorio de Virologa HUCA

Hamadan University of Medical Sciences, Department of Virology, Hamadan 0000000, Iran

Hangzhou CDC

Hangzhou Center for Diseases Control and Prevention

Hannover Medical School, Institute of Virology

HCRC

Health & Environment Institute of Gwangju

Health and Environmental Research Institute of Gwangju Metropolitan city

Hebei Provincial CDC, Shijiazhuang, Hebei Province; National Institute for Viral Disease Control and Prevention, China CDC

Helix

Hellenic Pasteur Institute, National Influenza Reference laboratory of Southern Greece & Unit of Bioinformatics and Applied Genomics

Hellenic Pasteur Institute, Public Health Laboratories, Unit of Bioinformatics and Applied Genomics

1. Laboratory of Recombinant Vaccines 2. Department of Biology and Medical Genetics 3. Laboratory of Clinical Genetics

Laboratory of Recombinant Vaccines

Hematopathology Laboratory, ACTREC, TMC

Hemocentro de Ribeirao Preto/FMRP-USP

Universidade Federal de Ciencias da Saude de Porto Alegre

Hiroshima Prefectural Technology Research Institute, Public Health and Environment Center

HIV Molecular Lab

HIV Molecular Laboratory

HIV Molecular Laboratory, Ethiopian Public Health Institute

HIV molecular lab, Ethiopian Public Health Institute, Ethiopian

Laboratorio INAC

School of Public Health, The University of Hon g Kong

Hopital Europeen George-Pompidou

Hospital So Rafael - IDOR

Laboratorio de Genmica Microbiana, Universidad Peruana Cayetano Heredia

HOSPITAL UNIVERSITARIO SON ESPASES

Nodo de Secuenciacin Tierra del Fuego - Hospital Regional Ushuaia - Centro Austral De Investigaciones Cientficas - Universidad Nacional De Tierra Del Fuego on behalf of 'Proyecto Argentino Interinstitucional de genomica de SARS-CoV-2' (PAIS Consortium)

Charit Virology-University of Costa Rica

SeqCOVID-SPAIN consortium/Institute of Biomedicine of Valencia, IBV-CSIC

Hospital Clnico Universitario Virgen de la Arrixaca

Instituto Ren Rachou / Fiocruz Minas

Laboratorio de Virologa / Hospital Universitario Central de Asturias (HUCA)

HOSPITAL UNIVERSITARIO 12 DE OCTUBRE

Unidad de Secuenciacin y Genmica, USACH

Hritas

Hospital Universitari Vall d'Hebron - Vall d'Hebron Institut de Rercerca

Centro de Innovacin en Vigilancia Epidemiolgica (CiVE), Institut Pasteur Montevideo, Uruguay

1. Virogenetics Laboratory of Virology, Maopolska Centre of Biotechnology, Jagiellonian University. 2. Intercollegiate Faculty of Biotechnology University of Gdansk and Medical University of Gdansk

Laboratorio nacional-LANIIA-CIAD

Hospital General de Mxico, Medicina Genmica

Hospital General Universitario de Ciudad Real

Asociacin de Salud Integral/Clnica Familiar Luis ngel Garca
SeqCOVID-SPAIN consortium / IBV (CSIC)
Hospital Israelita Albert Einstein
Instituto Adolfo Lutz Interdisciplinary Procedures Center Strategic Laboratory
Institute for Medical Research, Infectious Disease Research Centre, National Institutes of Health, Ministry of Health Malaysia Jalan Setia Murni U13/52, Bandar Setia Alam, 40170 Selangor, Malaysia
CIAD LDM-LGM
Microbial Genomics Laboratory
Instituto Butantan (genome assembly and bioinformatics) and Mendelics (sequencing)
Bushman
Bushman Lab - University of Pennsylvania
Laboratorio de Virologa Hospital Universitario Central de Asturias (HUCA)
Instituto de Patologia Vegetal (CIAP-INTA) on behalf of 'Proyecto Argentino Interinstitucional de genomica de SARS-CoV-2' (PAIS Consortium)
Institute for Medical Research, Infectious Disease Research Centre, National Institutes of Health, Ministry of Health
Hospital Universitari Vall d'Hebron
Hospital Universitari Vall d'Hebron - Vall d'Hebron Institut de Recerca
Hospital Universitari Vall d'Hebron - Vall d'Hebron Research Institute
Hospital Universitari Vall d'Hebron - Vall Hebron Institut de Recerca
Hospital Universitari Joan XXIII
Hospital Universitari Vall d'Hebron -
Hospital Universitari Vall d'Hebron - Vall d'hebron Research Institut (VHIR)
Hospital Universitari Vall d'Hebron - Vall d'Hebron Research Institute (VHIR)
Hospital Universitari Vall d'Hebron (HUVH) - Vall d'Hebron Research Institute (VHIR)
Hospital Universitario 12 de Octubre
Hospital Universitario 12 de Octubre
Hospital Universitario La Paz
Hospital Universitario La Paz
Hospital Universitario Marqus de Valdecilla
Hospital Universitario Son Espases
Houston Health Department
Houston Health Department, Disease Prevention and Control
Houston Health Department, Disease Prevention and Control
Houston Health Dept.
Houston Methodist Hospital
IAME UMR1137 Inserm, Universit de Paris, Hpital Bichat
Centre Hospitalier Universitaire Dijon Laboratoire de Virologie Plateforme de Biologie Hospitalo-Universitaire
Hpitaux universitaires de Genve Laboratoire de Virologie
Hpitaux universitaires de Genve Laboratoire de Virologie
Hrvatski zavod za javno zdravstvo
Hubei Provincial CDC
IPEC Guarapuava
Human Genetic Research Center, Kawsar Biotech Company, Majlesi St, Valiasr Ave, Tehran 1595645513, Iran
Human Genome Center
Humboldt County Public Health Laboratory
National Laboratory of Virology, Szentgothai Research Centre
Ibaraki Prefectural Institute of Public Health
ICAR-National Institute of High Security Animal Diseases
University Campus Bio-Medico di Roma
CSIR Institute of Genomics and Integrative Biology (CSIR-IGIB)
Boise VA Medical Center, PALMS
IBL
Department of Molecular Biology, Medical Research Institute, Colombo
IDEEL
ITV-Vale Institute of Technology
ID Genomics
IEH and ID Genomics
19-21, boulevard Jean Moulin, 13005 Marseille
IHU Mditerrane Infection
IHU Mediterranee Infection

IICS-UNA
IIP Institute of Genomics and Integrative Biology
IL Department of Public Health Chicago Laboratory
RCMI-Center for Research Resources, Ponce Research Institute
ILTM
Molecular Genetics Laboratory-Faculty of Medicine-Universitas Padjadjaran; School of Life Sciences and Technology & School of Pharmacy-Institut Teknologi Bandung; Laboratorium Kesehatan Provinsi Jawa Barat
Immunogenomics lab, Institute of Life Sciences, Bhubaneswar
Immunology, Noguchi Memorial Institute for Medical Research
Imperial College London
Laboratorio Mixto de Biotecnologa Acutica (LMBA)
Incubadora Venezolana de Ciencia, Venezuela / Instituto Nacional de Salud, Bogot, Colombia / Grupo de Investigaciones Microbiolgicas-UR (GIMUR), Departamento de Biologa, Facultad de Ciencias Naturales, Universidad del Rosario, Bogot, Colombia / Icahn School of Medicine at Mount Sinai, New York, USA
National Influenza Center, Indian Council of Medical Research-National Institute of Virology
Indian Council of Medical Research-National Institute of Virology, Microbial Containment Complex
Indian Council of Medical Research-National Institute of Virology,Maximum Containment Laboratory
National Influenza Center, Indian Council of Medical Research - National Institute of Virology
Carpi Laboratory - Purdue University
Quantigen Biosciences
Indiar Gandhi Memorial Hospital
NCDC Delhi, Biotechnology Division INSACOG
Infection and Immunology, Translational Health Science and Technology Institute
Central Virology laboratory, Ministry of Health
GIMAP and Virpath teams-CIRI
Infectious Disease Biology, Institute of Life Sciences
Infectious Disease Biology, Institute of Life Sciences
Infectious Disease Control and Prevention Institute
Infectious Disease Control Center, CDC of PLA
Infectious Disease Core Research, Abbott Diagnostics Division
Infectious Disease Department of Epidemiology, Beijing Institute of Microbiology and Epidemiology
Planet Lab, Children's Hospital of Philadelphia
Infectious Disease Program, Broad Institute of Harvard and MIT
Infectious Disease, JHU SARS-CoV-2 Genome Sequencing, 3400 N. Charles St., Baltimore, MD 21218, USA
Instituto Nacional de Ciencias Medicas y Nutricion Infectious Diseases
Infectious Diseases, Chan-Zuckerberg Biohub Covidtracker
Infectious Diseases, Columbia University
Human virology Department
Human Virology Department
Human virology deprtment
Pathogenic Microorganisms Variability Laboratory
Influenza Virus Research Center, National Institute of Infectious Diseases
Laboratorio de Virologa Molecular
Innovative Genomics Institute, UC Berkeley
Innovative Genomics Institute, UCB
InnovoLab Chile
Inovie AS GenBio
Inovie AS Genbio Clermont-Ferrand
Inovie AS GenBio Clermont-Ferrand
National Institute of Biomedical Genomics INSACOG
INSACOG at CSIR Institute of Genomics and Integrative Biology
Insititut Paasteur du Maroc
INSPI-Centro de Investigacin Multidisciplinaria de la DTIDI
NIC-INSPI
Av. Julin Coronel 905 entre Esmeraldas y Jos Mascote Av. Juan Tanca Marengo No. 100 y Av. de las Amricas
Inst. for Med. Virology, University Hospital Frankfurt, Goethe University Frankfurt
Inst. Hygiene
Department of Biosystems Science and Engineering, ETH Zrich
Institut de Pathologie et Genetique

Institut de Pathologie et Gntique

Institut de virologie du CHU de Strasbourg

Institut de Virologie du CHU de Strasbourg

Institute of Virology, Clinial Virus Genomics, Medical Center, University of Freiburg, Freiburg, Germany

Institut fr Medizinische Virologie, Universittsklinikum Frankfurt

NGS Competence Center Tbingen, Institut fr Medizinische Mikrobiologie und Hygiene, Universittsklinikum Tbingen

Unit Mixte Internationale TransVIHMI (UMI 233 IRD U1175 INSERM - Universit de Montpellier) IRD (Institut de recherche pour le dveloppement)

Noguchi Memorial Institute for Medical Research, University of Ghana, Legon, Ghana

Unit Mixte Internationale TransVIHMI (UMI 233 IRD U1175 INSERM - Universit de Montpellier)IRD (Institut de recherche pour le dveloppement)

Institut Pasteur CIBU / ERI

Institut Pasteur CIBU /ERI

Institut Pateur de Dakar

Institut Pasteur de la Guadeloupe

Functional Genomic Plateform/CNRST

Institut Pasteur, Laboratory for Urgent Response to biological Threats

Institute of microbiology and Immunology, Faculty of Medicine, University of Belgrade

Institute for Computational Biomedicine, Weill Cornell Medicine

IEDCR-ideSHi-icddr,b

Institute for Forensic Medicine, University of Belgrade, Faculty of Medicine, dr Subotica 8, Belgrade, Serbia 11000, Serbia

Abbott

Insitute for medical Microbiology and hospital Hygene

Institute for medical Microbiology and hospital Hygene

Institute for Medical Research Infectious Disease Research Centre, National Institutes of Health, Ministry of Health Malaysia

Institute for Medical Research, Infectious Disease Research Centre, National Institutes of Health, Minis

Laboratory for advanced genomics

National Centre for Biological Sciences

Institute for Vector and Reservoir Control, Research and Development (IVRCRD)- National Institute of Health Research and Development (NIHRD), Indonesia

Institute for Virology, University Hospital Duesseldorf, Medical Faculty, Heinrich-Heine-University Duesseldorf

Institute information KU Leuven, Clinical and Epidemiological Virology

Virology Department Institute of Microbiology and Immunology Faculty of Medicine University of Belgrade

Virology department Institute of microbiology and immunology Faculty of Medicine University of Belgrade

Virology Department, Institute of microbiology and immunology, Faculty of Medicine University of Blegrade

Institute of Biomedical & Genetic Engineering

Department of Molecular & Medical Virology, Ruhr University Bochum, 44801 Bochum, Germany | Institute of Virology, Charit - Universittsmedizin Berlin, corporate member of Freie Universitt Berlin, Humboldt-Universitt zu Berlin and Berlin Institute of Health (BIH), Berlin, Germany

Institute of Diagnostic Virology, Friedrich-Loeffler-Institut

Institute of Disease Control and Prevention, People's Liberation Army

Institute for Developing Science and Health Initiatives

IEDCR-ideSHi-icddr

Institute of Genomics Core Facility, University of Tartu

Institute of Human Genetics, Polish Academy of Sciences

Institute of Human Virology, Zhongshan School of Medicine, Sun Yat-sen University

Institute of Life Sciences

Institute of Medical Biology, Chinese Academy of Medical Sciences and Peking Union Medical College, No. 935, Jiao Ling Road, Kunming, Yunnan 650118, China

Institute of Medical Genetics and Applied Genomics

Institute of medical Microbiology and hospital Hygiene

Institute of medical Microbiology and Hospital Hygiene, Otto-von-Guericke University Magdeburg

DRESDEN-concept Genome Center, CMCB, TU Dresden

Institute of Human Genetics, University Medcal Center Goettingen

The National Laboratory of Health, Environment and Food- Centre for Medical Microbiology Maribor

Institute of Microbiology Universidad San Francisco de Quito

Institute of Microbiology, University of Veterinary and Animal sciences

Institute of Molecular and Translational Medicine / Laboratory of Experimental Medicine

Institute of Organic Chemistry and Biochemistry of the CAS Virology

Institute of Pathogen Biology, Chinese Academy of Medical Sciences & Peking Union Medical College

Institute of Pathogen Biology, Chinese Academy of Medical Sciences & Peking Union Medical College; China National Center for Bioinformation

Institute of Pathogen Biology, Chinese Academy of Medical Sciences & Peking Union Medical College; Vision Medicals Co., Ltd

Institute of Pathology

Veterinary Specialized Institute Kraljevo

Institute of Tropical Disease, Universitas Airlanga

Institute of Virology Department of Hygiene, Microbiology and Public Health at Innsbruck Medical University

Mnch Lab / Ulm University Medical Center Kirchhoff Lab / Ulm University Medical Center Sparrer Lab / Ulm University Medical Center Blum Lab / LMU Munich

Faculty of Mathematics, Physics and Informatics, Comenius University, Bratislava

Faculty of Natural Sciences, Comenius University, Bratislava

Institute of Virology, Biomedical Research Center of the Slovak Academy of Sciences, Bratislava; Comenius University Science Park, Bratislava

Institute of Virology, Biomedical Research Center of the Slovak Academy of Sciences, Bratislava; Comenius University Science Park, Bratislava

Institute of Virology, University Hospital, University of Bonn and German Center for Infection Research (DZIF), Bonn-Cologne, Bonn, Germany

Institute of Virology, University of Cologne

Instituto Adolfo Lutz Laboratrio de Vrus Respiratrios

Instituto Adolfo Lutz, Interdisciplinary Procedures Center, Strategic Laboratory

Instituto Adolfo Lutz, Rapid Response Center, Strategic Laboratory

Instituto Adolfo Lutz, Interdiciplinary Procedures Center, Strategic Laboratory

Laboratrio Metabolismo Macromolecular FirminoTorres de Castro, Instituto de Biofsica Carlos Chagas Filho, Universidade Federal do Rio de Janeiro

Instituto Carlos Chagas - Fiocruz

Instituto de Biotecnologia - UNESP-Botucatu-SP

Instituto de Diagnostico y Referencia Epidemiologicos

Instituto de diagnstico y Referencia Epidemiologicos (INDRE)

Instituto de Diagnstico y Referencia Epidemiolgicos

Instituto de Diagnstico y Referencia Epidemiologicos

INEI - ANLIS. Instituto Nacional de Enfermedades Infecciosas, Administracin Nacional de. Laboratorios e Institutos de Salud Dr. Carlos G. Malbran'

INEI - ANLIS. Instituto Nacional de Enfermedades Infecciosas, Administracin Nacional de. Laboratorios e Institutos de Salud Dr. Carlos G. Malbran' /INBIRS

INEI - ANLIS. Instituto Nacional de Enfermedades Infecciosas, Administracin Nacional de. Laboratorios e Institutos de Salud Dr. Carlos G. Malbran'/ INBIRS

INEI - ANLIS. Instituto Nacional de Enfermedades Infecciosas, Administracin Nacional de. Laboratorios e Institutos de Salud Dr. Carlos G. Malbran'/INBIRS

INEI - ANLIS. Instituto Nacional de Enfermedades Infecciosas, Administracin Nacional de. Laboratorios e Institutos de Salud Dr. Carlos G. Malbran

INEI - ANLIS. Instituto Nacional de Enfermedades Infecciosas, Administracin Nacional de. Laboratorios e Institutos de Salud Dr. Carlos G. Malbran / INBIRS

Instituto de Medicina Tropical & Salud Global, Universidad Iberoamericana (UNIBE)

Centro de Investigaciones Agropecuarias (CIAP), Instituto Nacional de Tecnologa Agropecuaria (INTA), Crdoba, Argentina on behalf of Proyecto Argentino Interinstitucional de Genmica de SARS-CoV-2 (PAIS Consortium)

Centro de Investigaciones Agropecuarias (CIAP), Instituto Nacional de Tecnologia Agropecuaria (INTA), Crdoba, Argentina on behalf of Proyecto Argentino Interinstitucional de Genmica de SARS-CoV-2 (PAIS Consortium).

Laboratorio de Virologa, Hospital de Nios Ricardo Gutirrez, CABA, Argentina.

Laboratrio de Imunofarmacologia

Instituto Gulbenkian de Cincia

Instituto Gulbenkian de Cincia

Instituto Nacional de Ciencias Medicas y Nutricion

Instituto Nacional de Ciencias Medicas y Nutricion Salvador Zubiran

Instituto Nacional de Enfermedades Respiratorias

Instituto Nacional de Cancerologa

Instituto Nacional de Enfermedades Respiratorias (INER)

Laboratorio de Virologa Molecular, CMBC, IVIC

Laboratorio de Virologa Molecular, CMBC, IVIC.

Instituto Nacional de Investigacin en Salud Pblica

Instituto Nacional de Investigacin en Salud Publica

INSPI - Charit

Instituto Nacional de Investigacin y Tecnologa Agraria y Alimentaria (INIA)/ Departamento de Mejora Gentica Animal

Instituto Nacional de Investigacin en Salud Pblica

CERI, Centre for Epidemic Response and Innovation and KRISP, KZN Research Innovation and Sequencing Platform

CERI, Centre for Epidemic Response and Innovation, Stellenbosch Univeristy & KRISP, KZN Research Innovation and Sequencing Platform

Instituto Nacional de Medicina Genmica

Instituto Nacional de Enfermedades Respiratorias (INER), Centro de Investigacin en Enfermedades Infecciosas (CIENI)

Corporacion Corpogen Universidad de los Andes Universidad Central

Laboratorio de Infecciones Respiratorias Agudas

Instituto Nacional de Salud, Universidad Cooperativa de Colombia, Instituto Alexander von Humboldt, Imperial College-London, London School of Hygiene & Tropical Medicine

Instituto Nacional de Salud Universidad Cooperativa de Colombia Instituto Alexander von Humboldt Imperial College-London London School of Hygiene & Tropical Medicine

Instituto Nacional de Salud, Bogot, Colombia

Instituto Nacional de Saude (INSA) and Instituto Gulbenkian de Ciencia (IGC)
Instituto Nacional de Saude (INSA) and BioSystems & Integrative Sciences Institute (BioISI) Genomics Unit, FCUL
Instituto Nacional de Saude (INSA) and i3S - Instituto de Investigao e Inovao em Sade
Instituto Octvio Magalhes / Fundao Ezequiel Dias (IOM/Funed)
Laboratorio de Virologia, Faculdade de Medicina, Universidade Federal de Mato Grosso, campus Cuiab
Instituto Oswaldo Cruz FIOCRUZ - Laboratory of Respiratory Viruses and Measles (LVRS)
Institut of Human Genetics, University Medicine Goettingen
INT Fondazione Pascale
Molecular Biology Laboratory
International Livestock Research Institute
National Research and Innovation Agency (BRIN-Indonesia, VenomCoV Project Team)
1. Tricity SARS-CoV-2 sequencing consortium: University of Gdansk, Medical University of Gdansk, Vaxican Ltd., Invicta Ltd. 2. National Institute of Public Health - National Institute of Hygiene, Warsaw, Poland
Institut Pasteur du Laos
Iran National Influenza Center
Department of Infectious, Tropical Diseases & Microbiology,IRCCS Sacro Cuore Don Calabria Hospital
University of Verona, Department of Biotechnology
IRCCS Regina Elena National Cancer Institute
Iressef Genomics lab
IRESSEF
L'institut de Recherche en Sant, de Surveillance pidmiologique et de Formation (IRESSEF)
IRESSEF GENOMICS LAB
IrsiCaixa Retrovirology Lab
Ishikawa Prefectural Institute of Public Health and Enviromental Science
Israel Central Virology laboratory
Israel National Consortium for SARS-CoV-2 sequencing
Istanbul University-Cerrahpasa, Cerrahpasa School of Medicine, COVID-19 Laboratory
Istinye university genetic diseases assessment center
Istituto Auxologico Italiano
Istituto Nazionale Malattie Infettive Lazzaro Spallanzani IRCCS
Istituto Zooprofilattico Sperimentale del Mezzogiorno-U.O.C. Virologia
IZSM-U.O.C. Virologia
Lab. Microbiologia e Virologia, Cotugno, A.O. dei Colli
U.O. Diagnostica Virologica Dip. Sanit Animale IZSM
Telethon Institute of Genetics and Medicine - Telethon Institute of Genetics and Medicine - TIGEM
Telethon Institute of Genetics and Medicine - TIGEM
Istituto Zooprofilattico Sperimentale del Mezzogiorno (IZSM)
U.O. Genomics, S.S. Genetics and Advanced Omics Techniques Istituto Zooprofilattico Sperimentale del Piemonte, Liguria e Valle d'Aosta
Istituto Superiore di Sanit
Department of Food safety, Nutrition and Veterinary public health - Istituto Superiore di Sanit
Istituto Zooprofilattico Sperimentale dell'Abruzzo e del Molise
Istituto Zooprofilattico Sperimentale dell'Abruzzo e del Molise G. Caporale
Istituto Zooprofilattico Sperimentale dell'Abruzzo e del Molise G.Caporale
Istituto Zooprofilattico Sperimentale dell'Abruzzo e Molise G. Caporale
Istituto Zooprofilattico Sperimentale dell'Abruzzo e Molise G.Caporale
Beaconlab (Bioinformatics, Evolution and Comparative Genomics lab), Dept of Biosciences, University on Mila
Beaconlab (Bioinformatics Evolution and Comparative Genomics lab), Dept of Biosciences, University of Milan
Beaconlab (Bioinformatics, Evolution and Comparative Genomics lab), Dept of Biosciences, University on Milan
Illumina Miseq
IU-Cerrahpasa, Cerrahpasa School of Medicine, COVID-19 Lab
Mert Kuskucu, Yesim Tuyji Tok, Kenan Midilli
University of Michigan, Department of Microbiology and Immunology, Lauring Lab
University campus Bio-Medico di Roma
IZSM
Istituto Zooprofilattico Sperimentale del Mezzogiorno
Istituto Zooprofilattico Sperimentale del Mezzogiorno - Unit Operativa Complessa di Virologia
Virology Lab, Jaber Al Ahmad Al Sabah Hospital
Kuwait cancer control center
James Molecular Lab - OSUWMC

Jamil-ur-Rahman Center for Genome Research, Dr. Panjwani Center for Molecular Medicine and Drug Research
Jamil-ur-Rahman Center for Genome Research, Dr. Panjwani Center for Molecular Medicine and Drug Research, International Center for Chemical and Biological Sciences, University of Karachi
Jamil-ur-Rahman Center for Genome Research, Dr. Panjwani Center for Molecular Medicine and Drug Research, International Center for Chemical and Biological Sciences, University of Karachi, University Road, Karachi, Sindh 75270, Pakistan
Institute for infectious medicine & hospital hygiene, CaSe-Group
Institute of infectious medicine & hospital hygiene, CaSe-Group
Jessa Ziekenhuis
Jiangsu Provincial CDC
Jiangsu Provincial Center for Disease Control & Prevention
Jiangxi province CDC
Jiangxi Province CDC
Institute of Parasitology, Biology Centre, CAS
Jining CDC
Johns Hopkins Hospital Department of Pathology
Jordan Royal Medical Services Oncology Lab
Princess Haya Biotechnology Center/ Jordan University of Science   Technology
Juntendo University Hospital
Kagawa Prefectural Research Institute for Environmental Sciences and Public Health
Kagoshima Prefectural Institute for Environmental Research and Public Health
OHSU-MM Lab
OHSU MM Lab
Kanagawa Prefectural Institute of Public Health
Kanagawa Prefectural Institute of Public Health; 1-3-1 Shimomachiya, Chigasaki, Kanagawa 253-0087, Japan
Clinical Reference Laboratory
Cytocheck Laboratory
Karolinska University Hospital
Kashi Clinical Laboratory
Nepal Health Research Council
Tokyo Medical University Department of Microbiology
Kawsar Human Genetic Research Center
Kawsar Human Genetic Research Company
Keio University Hospital
Keio University School of Medicine
KEMRI & ICGEB
KEMRI-Wellcome Trust Research Programme/KEMRI-CGMR-C Kilifi
KEMRI-Wellcome Trust Research Programme,Kilifi
Key Laboratory of Human Diseases Comparative Medicine, Institute of Laboratory Animal Science, CAMS&PUMC
King Faisal Specialist Hospital & Research Center
King Faisal Specialist Hospital & Research Centre
CSIR-National Botanical Research Institute
Queen's Genomics Lab at Ongwanada (Q-GLO)
Ontario Institute for Cancer Research
Lithuanian University of Health Sciences
Klinisch Laboratorium ZNA
Unit for Biological Agents, Department for CBRN Defence and Security, Swedish Defence Research Agency
Kochi Public Health and Environmental Science Research Institute
Korea Centers for Disease Control & Prevention (KCDC) Center for Laboratory Control of Infectious Diseases Division of Viral Diseases
Genetics Working Group (Pokja Genetik) Faculty of Medicine, Public Health and Nursing Universitas Gadjah Mada (FK-KMK UGM); Disease Investigation Center Wates Ministry of Agriculture Indonesia; Department of Microbiology FK-KMK UGM; Laboratorium Diagnostik Yayasan Tahija World Mosquito Program (WMP) Yogyakarta Center for
Koupenova Lab
KRISP, KZN Research Innovation and Sequencing Platform
Marshfield Clinic Research Institute - Integrated Research and Development Laboratory
KU Leuven, Clincal and Epidemiological Virology
KU Leuven, Clinical and Epidemiological Virology
Kumamoto City Environmental Reserch Institute
Sato Laboratory, Division of Genomics and Transcriptomics, The Joint Research Center for Human Retrovirus Infection, Kumamoto University
Kumamoto Prefectural Institute of Public-Health and Environmental Science
Institute of Virology, Charit Universittsmedizin Berlin

Human Genetics Laboratory, National Institute of Genetics
Hong Kong Childrens Hospital
Kyoto Prefectural Institute Of Public Health And Environment
CDC Division of Viral Diseases, Pathogen Discovery
Lab voor klinische biologie
Onderzoeksgroep Virologie
Laboratorio de Biologia Molecular, Universidad Cooperativa de Colombia, Santa Marta
LABCOVID_HCPA
LABRESIS_HCPA
Labinfo, LNCC
Laboratorio di Microbiologia
Laboratoire de Biotechnologie
Functional Genomic Platform_UATRS_CNRST_Rabat
Valais Hospital, Central Institute
Laboratoire de Biologie Mdicale Hpital Sainte Musse
Laboratoire de biologie molculaire - Plateforme Clinique
IHU Mditerrane Infection.
Laboratoire de microbiologie CHU NIMES
Smith Laboratory, Centre de Recherche CHU Sainte-Justine
Laboratoire de Recherche et d'Analyses Mdicales de la Gendarmerie Royale
1-Laboratory of Microbiology, National Reference Lab, Charles Nicolle Hospital; 2-University of Tunis ElManar, Faculty of Medicine of Tunis, LR99ES09, Tunis, Tunisia
GRAM2.0, Universit de Caen Normandie
GRAM2.0, Universit de Caen Normandie Laboratoire de Virologie, CHU de Caen
Swiss National Reference Centre for Influenza
Charit-Universittsmedizin Berlin
Institut fr Virologie - Institute of Virology - Charite
Laboratoire Hpital Sainte Musse
Pathogen Genomics Lab, National Institute for Biomedical Research (INRB)
Genomics and Proteomics Departament, Gorgas Memorial Institute For Health Studies
Laboratoire National de Sant, Microbiology, Epidemiology and Microbial Genomics
Laboratoire national de sant, Microbiology, Epidemiology and Microbial Genomics
Laboratoire national de sant, Microbiology, Microbial Genomics Platform
Laboratoire National de Sante, Microbiology, Epidemiology and Microbial Genomics
Laboratoire Nationale de Sant, Microbiology, Epidemiology and Microbial Genomics
Laboratoire Virpath, CIRI U111, UCBL1, INSERM, CNRS, ENS Lyon
Laboratoire Virpath, CIRI U111, UCBL1, INSERM, CNRS, ENS Lyon
Can Ruti SARS-CoV-2 Sequencing Hub (HUGTiP/IrsiCaixa/IGTP)
LABORATORI NACIONAL EPIDEMIOLOGIA
LBM de CHU de Toulouse, Hpitaux de Toulouse
Hemocentro de Ribeirao Preto FMRP USP
Laboratorio de Biologia Molecular Hemocentro de Ribeirao Preto FMRP USP
Laboratorio de Biologia Molecular-Hemocentro de Ribeirao Preto
Laboratorio de Genmica Microbiana, Departamento Biomdico, Facultad de Ciencias de la Salud, Universidad de Antofagasta.
LABORATORIO SPECIALISTICO UOC EMATOLOGIA- Ospedale San Francesco - ATS-ASSLNUoro
Laboratorio specialistico UOC Ematologia - Ospedale San Francesco- ATS-ASSL Nuoro
Laboratorio Biologia Molecolare Sars Cov2 - UOC Laboratorio Analisi - Servizio Medicina di Laboratorio, Ospedale San Francesco - ATS-ASSL Nuoro
Laboratorio specialistico UOC Ematologia - Ospedale 'San Francesco' - ATS-ASSL Nuoro
Laboratorio Biologia Molecolare Sars Cov2 - UOC Laboratorio Analisi - Servizio Medicina di Laboratorio, Ospedale San Francesco - ATS-ASSL Nuoro and Laboratorio specialistico UOC Ematologia - Ospedale San Francesco - ATS-ASSL Nuoro
Laboratorio Biologia Molecolare Sars Cov2 - UOC Laboratorio Analisi - Servizio Medicina di Laboratorio, Ospedale San Francesco - ATS-ASSL Nuoro and Laboratorio specialistico UOC Ematologia - Ospedale San Francesco - ATS-ASSL Nuoro
Laboratorio Specialistico Ematologia, UOC Ematologia, Ospedale San Francesco - ATS-ASSL Nuoro
Laboratorio specialistico UOC Ematologia - Ospedale San Francesco - ATS-ASSL Nuoro
Laboratorio specialistico UOC Ematologia - Ospedale San Francesco - ATS-ASSL Nuoro
Laboratorio Specialistico UOC Ematologia Ospedale San Francesco - ATS-ASSL Nuoro
Laboratorio Specialistico UOC Ematologia, Ospedale San Francesco - ATS-ASSL Nuoro
Laboratorio specialistico UOC Ematologia - Ospedale San Francesco - ATS-ASSL Nuoro Nuoro
Laboratorio Specialistico UOC Ematologia Ospedale San Francesco - ATS ASSL NUORO
Laboratorio Central de Epidemiologa-DLVIE / Laboratorio de Secuenciacin-Centro de Instrumentos. Instituto Mexicano del Seguro Social

Grupo de Genmica y Bioinformtica del Instituto de Investigacin de la Cadena Lctea CONICET-INTA
Laboratorio Central de Salud Publica de Paraguay
Laboratorio de Ecologia de Doencas Transmissiveis na Amazonia, Instituto Leonidas e Maria Deane - Fiocruz Amazonia
Laboratrio de Biologia Molecular da Universidade Federal de Cincias da Sade de Porto Alegre
Laboratorio Central do Estado do Parana
Hospital Regional Ushuaia - Centro Austral De Investigaciones Cientficas - Universidad Nacional De Tierra Del Fuego
Instituto de Patologia Vegetal (CIAP-INTA)
Sequencing and Genomics facility (SGF)
Gencore - Universidad de los Andes.
MELISA Institute Genomics and Proteomics SpA
Universit degli Studi di Palermo
Laboratorio de Infectologa Molecular, Departamento de Bioqumica y Medicina Molecular,Facultad de Medicina - Universidad Autnoma de Nuevo Len
Centro de Gentica y Biologa Molecular - Universidad del Magdalena
LABORATORIO DE PATOLOGA MOLECULAR CENTRO MEDICO NAVAL SEMAR
LABORATORIO DE PATOLOGA MOLECULAR CENTRO MEDICO NAVAL SEMAR
Laboratorio de Genmica Microbiana, Departamento Biomdico, Facultad de Ciencias de la Salud.
Laboratorio de Biologia Molecular de Flavivirus, Instituto Oswaldo Cruz
Instituto de Biotecnologa, IABIMO (CONICET), Instituto de Virologa, IVIT(CONICET), Instituto de Patobiologa, IPVET(CONICET), CICVyA, INTA
Departamento de Ciencias Bsicas Universidad Nacional de Lujn y Unidad de Genmica Instituto Nacional de Tecnologa Agropecuaria on behalf of 'Proyecto Argentino Interinstitucional de genomica de SARS-CoV-2' (PAIS Consortium)
Laboratorio de Diagnstico-UNIDAD COVID- Universidad Nacional de Hurlingham y Unidad de Genmica Instituto Nacional de Tecnologa Agropecuaria on behalf of 'Proyecto Argentino Interinstitucional de genomica de SARS-CoV-2' (PAIS Consortium)
Laboratorio de Diagnstico-UNIDAD COVID- Universidad Nacional de Hurlingham y Unidad de Genmica Instituto Nacional de Tecnologa Agropecuaria on behalf of 'Proyecto Argentino Interinstitucional de genomica de SARS-CoV-2' (PAIS Consortium)
Laboratorio de Enfermedades Emergentes y Reemergentes
Laboratorio de Infectologa Molecular Departamento de Bioqumica y Medicina Molecular Facultad de Medicina - Universidad Autnoma de Nuevo Len
Laboratory of Molecular Virology, School of Medicine, Pontificia Universidad Catolica de Chile
Centro Asistencial Docente y de Investigacion, Universidad de Magallanes
Laboratorio de Estudos de Virus Emergentes
Laboratorio de Estudos de Virus Emergentes - UNICAMP
Laboratorio de Pesquisa em Virologia, FAMERP, SJRP
Laboratorio de Referencia Nacional de Biotecnologa y Biologa Molecular. Instituto Nacional de Salud Peru
Laboratorio de Referencia Nacional de Biotecnologia y Biologia Molecular.Instituto Nacional de Salud.Peru
Laboratorio de Referencia Nacional de Enteropatgenos. Instituto Nacional de Salud del Per
Laboratorio de Referencia Nacional de Biotecnologa y Biologa Molecular. Centro Nacional de Salud Publica. Instituto Nacional de Salud Peru.
Laboratorio de Referencia Nacional de Biotecnologa y Biologa Molecular. Instituto Nacional de Salud Per
Laboratorio de Referencia Nacional de Biotecnologa y Biologa Molecular. Instituto Nacional de Salud Per.
Laboratorio de Referencia Nacional de Biotecnologa y Biologa Molecular. Instituto Nacional de Salud.
Laboratorio de Referencia Nacional de Biotecnologa y Biologa Molecular. Instituto Nacional de Salud.Per
Laboratorio de Referencia Nacional de Biotecnologia y Biologia Molecular.Instituto Nacional de Salud.Per
Laboratorio de Referencia Nacional de Virus Respiratorios. Centro Nacional de Salud Publica. Instituto Nacional de Salud Peru.
Laboratorio de Referencia Nacional de Biotecnologia y Biologia Molecular. Instituto Nacional de Salud Peru
Laboratorio de Referencial Nacional de Virus Respiratorios
Gencore- Universidad de los Andes
Instituto Nacional de Salud- Direccion de Investigacion en Salud Publica
Laboratorio de Salud Pblica de Bogot
LABOPAT
Biocdices SA.
Biocdices SA. on behalf of 'Proyecto Argentino Interinstitucional de genomica de SARS-CoV-2' (PAIS Consortium)
Laboratorio de Virologa y Microbiologa Molecular, Depto. de Microbiologa, Facultad de Medicina, Universidad de El Salvador
Laboratorio de Virologa y Microbiologa Molecular, Depto. de Microbiologa, Facultad de Medicina, Universidad de El Salvador.
Laboratorio de Virologa y Microbiologa Molecular, Depto. de Microbiologa, Facultad de Medicina, Universidad de El Salvador/INS-laboratorio de Ref. Ministerio de Salud
Laboratorio de Virologa y Microbiologa Molecular, Depto. de Microbiologa, Facultad de Medicina, Universidad de El Salvador/INS-laboratorio de Ref. Ministerio de Salud.
Laboratorio de Virologia del HUCA
Laboratorio de Virologia HUCA
Laboratorio di Genetica Medica Matera, Italy
Laboratorio di Genetica Medica, Matera, Italy
Laboratorio di Microbiologia e Virologia, Universit Vita-Salute San Raffaele, Milan

U.O. Igiene, Ospedale Policlinico San Martino
Istituto Zooprofilattico Sperimentale della Sicilia
Laboratorio di Riferimento Regionale della Sicilia Occidentale per lEmergenza COVID-19
Laboratorio Estatal de Salud Publica de Nuevo Leon
Instituto Nacional de Salud- Direccin de Investigacin en Salud Pblica, Universidad de los Andes- Gencore
Laboratorio SPOKE Biologia Molecolare -Azienda Ospedaliero Universitaria - AOU Cagliari
Instituto Butantan / USP-Pirassununga
laboratorio micorbiologia PO Cardarelli
Laboratorio Microbiologia e Virologia P.O. Cotugno A.O. dei Colli
Laboratorio Microbiologia e Virologia, P.O. Cotugno, A.O. dei Colli
Laboratorio Microbiologia P.O. Cardarelli
laboratorio microbiologia PO Cardarelli
laboratorio Microbiologia PO Cardarelli
Laboratorio microbiologia PO Cardarelli
Asociacin de Salud Integral / Clnica Familiar Luis ngel Garca
Laboratorio Nacional de Salud
Submitting lab:
Laboratorios Clnicos de Puebla
1. Tricity SARS-CoV-2 sequencing consortium: University of Gdansk, Medical University of Gdansk, Vaxican Ltd., Invicta Ltd. 2. National Institute of Public Health - National Institute of Hygiene, Warsaw, Poland
Indonesian Institute of Sciences (LIPI) and Laboratorium Kesehatan Daerah (Labkesda) Kota Depok
Indonesian Institute of Sciences (LIPI) and Laboratorium Riset Universitas Jenderal Soedirman
National Research and Innovation Agency (BRIN-Indonesia, VenomCoV Project Team); Laboratorium Riset Unsoed
Department of Microbiology, Russian Anti-Plague Research Institute
Laboratory Diagnostic, Veterinary Specialized Institute Kraljevo
Laboratory Diagnostic, Veterinary Specialized Institute Kraljevo
Laboratory Diagnostic, Veterinary Specialized Institute Kraljevo, Zicka 34, Kraljevo 360103, Serbia
genXone SA, Research & Development Laboratory; The Faculty of Mathematics, Informatics and Mechanics of the University of Warsaw
Laboratory for Functional Genome Analysis, Dept. Genomics, Gene Center of the LMU Munich
Laboratory for HIV and opportunistic infections diagnosis The Republican Research and Practical Center for Epidemiology and Microbiology (RRPCEM)
Eurofins Genomics Europe Sequencing GmbH
Laboratory for Molecular Diagnostics, IPHMN
Laboratory Medicine and Molecular Diagnostics
Laboratory Medicine, University of Washington
University of Washington, Laboratory Medicine
Laboratory Medicine, University of Washington
Laboratory Medicine, University of Washington, 1100 Fairview Ave N, PO Box 19024, E5-110, Seattle, WA 98109, USA
Laboratory Molecular Biology, Hemocentro de Ribeiro Preto, FMRP-USP
Laboratory of Applied Genetics
Laboratory of Biology, Department of Medicine, Democritus University of Thrace
Laboratory of Biotechnology, Center for Advanced Technologies
LABORATORY OF CLINICAL MICROBIOLOGY, FACULTY OF MEDICINE, UNIVERSITY OF INDONESIA
Laboratory of Communicable Diseasea
1. Laboratory of Communicable Diseases (Estonia); 2. Eurofins Genomics Europe Sequencing GmbH
Laboratory of Communicable Diseases
University of Florida
Laboratory of Experimental Virology, Somov Institute of Epidemiology and Microbiology
Group of Genomics and Postgenomic Technologies of Central Research Institute of Epidemiology
Group of Genomics and Postgenomic Technologies of Central Research Institute of Epidemilology
Laboratory of Genetics and Personalized Medicine, Zan Mitrev Clinic
Laboratory of Genomics & Bioinformatics, Institute of Immunology and Experimental Therapy, Polish Academy of Sciences
Prof. Gorgoulis Lab
Laboratory of Histology-Embryology
Instituto Nacional de Salud - Unidad de Secuenciacin y Genmica
Laboratory of Infectious Diseases Center of Beijing Ditan Hospital
Laboratory of Microbiology and Virology, Ospedale Amedeo di Savoia, ASL Citt di Torino
Laboratory of Microbiology, ASST Ospedale di Circolo, Varese
Laboratory of Microbiology, ASST Ospedale di Circolo, Varese viale borri 57 21100 Varese, Italy
Laboratory of Microbiology, ASST Settelaghi, Varese, Italy

Clinical and Experimental Pharmacology Lab, LR16SP02, National Center of Pharmacovigilance, University of Tunis El Manar, Tunis, Tunisia. 2- Neurodegenerative diseases and psychiatric troubles, LR18SP03, Razi Hospital, University of Tunis El Manar, Tunis, Tunisia. 3- Ministry of Health, National Observatory of New and Emerging Diseases, 1006, Tunis, Tunisia

Laboratory of Microbiology, University of Insubria and Ospedale Di Circolo, Varese, Italy

Laboratory of Microbiology, Varese

Quadram Institute Bioscience

Microbial Pathogenomics Lab - LAU

Laboratory of Molecular Biology, Blood Center of Ribeiro Preto, Faculty of Medicine of Ribeiro Preto, University of So Paulo

Laboratory of Molecular Biology, Hemocentro de Ribeiro Preto

Laboratory of Molecular Genetics

Laboratory of Molecular Genetics, 2nd Faculty of Medicine, Charles University in Prague, Prague, Czech Republic

Laboratory of Molecular Genetics, 2nd Faculty of Medicine, Charles University in Prague, Prague, Czech Republic

Laboratory of Molecular Virology

Laboratory of Molecular Virology, Department of Biomedical, Surgical and Dental Sciences University of Milano

Biobank Lab, Department of Molecular Biophysics, Faculty of Biology and Environmental Protection, University of Lodz

Laboratory of Respiratory Viruses Teaching and Clinical Center of the Medical University of Lodz

Laboratory of Virology

Laboratory of virology and molecular diagnostics

Institute of Public Health of Republic of North Macedonia Laboratory of Virology and Molecular Diagnostics

Laboratory of Virology, INMI Lazzaro Spallanzani IRCCS

Laboratory of Virology, INMI Lazzaro Spallanzani IRCCS, via Portuense 292, Rome 00149, Italy

Laboratory of molecular-genetic research, National Center for Expertise, Kazakhstan National Center for Biotechnology, Kazakhstan

Laboratory of molecular-genetic research, National Center of Expertise, Kazakhstan National Center for Biotechnology, Kazakhstan
Abdaliyev Askar, Tungushbayev Talgat, Sharipova Saule, Shevtsov Alexandr, Amirgazin Asylulan, Kamalova Dinara, Ramankulov Erlan, Balykbaev Kanat

National Center for Expertise, Kazakhstan Zonal Virology Laboratory

Laboratory of Oncology, Blood Center of Ribeiro Preto, Ribeiro Preto School of Medicine, University of So Paulo

Laboratory, Gravity Diagnostics

Laboratoy of Molecular Biology, Hemocentro de Ribeiro Preto, FMRP-USP

Bioinformatics and Biotechnology Laboratory (Labinftec/Federal University of Tocantins, Campus of Gurupi)

Laboratrio de Biologia Integrativa

Laboratrios de Genmica Funcional (FCA/UNESP) e Biologia Molecular (FMB-HC/UNESP) - Rede de Vigilncia Genmica (Vigenmica)/UNESP

Laboratrios Genmica Funcional (FCA/UNESP) e Biologia Molecular (FMB-HC/UNESP) - Rede de Vigilncia Genmica (Vigenmica)/UNESP

Laboratrio Baculovrus

Laboratrio de Imunofarmacologia - Instituto Oswaldo Cruz

Laboratrio de Microbiologia Molecular - Universidade FEEVALE

Molecular Microbiology Laboratory

Universidade Federal de Cincias da Sade de Porto Alegre

Laboratrio de Estudos de Vrus Emergentes - UNICAMP

Laboratrio de Pesquisa em Virologia, FAMERP, SJRP

Laboratrio de Virologia Molecular - Universidade Federal do Rio de Janeiro

Laboratrio de Virologia Molecular da Instituto Carlos Chagas da Fundao Oswaldo Cruz

Laboratrio de Biologia Integrativa, Instituto de Cincias Biolgicas, Universidade Federal de Minas Gerais

LABRESIS

State Center for Health Surveillance. Rio Grande do Sul State Secretary of Health (CEVS)

WallauLab on behalf of Fiocruz COVID-19 Genomic Surveillance Network

LABBE, Federal University of Pernambuco

WallauLab, Aggeu Magalhaes Institute

CEVS_SES_RS

State Center for Health Surveillance. Rio Grande do Sul State Secretary of Health

Kansas State University Veterinary Diagnostic Laboratory

Kansas State Veterinary Diagnostic Laboratory

United States Air Force School of Aerospace Medicine

US Air Force School of Aerospace Medicine

LANIIA-Nayarit

LATE - Laboratrio de Tcnicas Especiais - Hospital Israelita Albert Einstein

LBM ALPHABIO

Lebanese American University

Lednicky Laboratory at Emerging Pathogens Institute, University of Florida

Lednicky Laboratory, Emerging Pathogens Institute, University of Florida.

Lee Lab
Leiden University Medical Center
LESP Hidalgo GENE2LIFE
LESP HIDALGO GENE2LIFE
Instituto de diagnstico y Referencia Epidemiologicos (INDRE) Departamento de Virologa
T4 OLIGO
LHUB-ULB
LIC
Institute of Biotechnology, Life Sciences Center, Vilnius University
Lithuanian University of Health Sciences, Laboratory of Molecular Cardiology
Lithuanian University of Health Sciences, Molecular cardiology lab.
Medical Biotechnology Laboratory, Rabat Medical and Pharmacy School, Mohammed The Vth University in Rabat
Los Angeles County Public Health Laboratory
Northwestern University - Ozer Lab
LNCC
Maasstad ziekenhuis
U.S. Air Force School of Aerospace Medicine
Maine Health and Environmental Testing Laboratory
Tewhey Lab, The Jackson Laboratory
Health and Environmental Testing Laboratory
Integrative Pharmacogenomics Institute (iPROMISE)
Mako Medical Laboratories
Malawi Liverpool Wellcome Trust CLinical Research Program
Malaysia Genome Institute
New Jersey Public Health and Environmental Laboratories (NJ PHEL)
New Jersey Public Health and Environmental Laboratories (PHEL)
Maryland Department of Health Laboratories Administration
Maryland Public Health Laboratory
Maryland Public Health Laboratory (MD PHL)
Mason Lab
Massachusetts State Public Health Laboratory
Public Health Virology Laboratory
Laboratory for Functional Genome Analysis; Dept. Genomics; Gene Center of the LMU Munich
Maximum Containment Laboratory, National Institute of Virology
University of Washington Virology Lab
Medical Ain Shams Research Institute (MASRI), Ain Shams University
Medical Genetics Department, Kocaeli University
Medical Genetics Laboratory, Regional Centre of Medical Genetics, Emergency County Hospital Craiova
Medical Genetics, Pamukkale University
Medical Microbiology and Zoology Section, Okinawa Prefectural Institute of Health and Environment
Medical Microbiology, Radboud University Medical Center
Medical Research Center, Faculty of Medicine, Syarif Hidayatullah State Islamic University Jakarta
Medicina Genmica
Medicine and Surgery, University of Insubria
Medicine and Surgery, University of Insubria
University of Insubria
Medtimes Molecular Laboratory
MEPHI Aix Marseille University (AMU)
Michigan Department of Health and Human Services, Bureau of Laboratories
Micrbiological Laboratory,Lu`an CDC
No.409,Gaocheng Middle Road,Lu`an City,Anhui Provice
Micrbiology Laboratory,Lu`an CDC
Microbial Genome Sequencing Center, Microbial Genomic Epidemiological Laboratory
Microbial Genomics Laboratory, Institut Pasteur de Montevideo
Microbial Genomics Laboratory, Institut Pasteur Montevideo
Microbial Genomics Laboratory, Institut Pasteur Montevideo, Uruguay
Microbiologia e Virologia
Microbiologia e Virologia Cotugno

MDU-PHL
Microbiological Diagnostic Unit - Public Health Laboratory (MDU-PHL)
Microbiological Diagnostics Unit Public Health Laboratory
Microbiological Diagnostic Unit Public Health Laboratory
Microbiological Diagnostic Unit Public Health Laboratory, The Peter Doherty Institute for Infection and Immunity
Microbiological Diagnostic Unit Public Health Laboratory (MDU-PHL) and Victorian Infectious Diseases Reference Laboratory (VIDRL), The Peter Doherty Institute for Infection and Immunity, 792 Elizabeth Street, Melbourne, Vic 3000, Australia
Microbiological Diagnostic Unit Public Health Laboratory and Victorian Infectious Diseases Reference Laboratory, Doherty Institute
Microbiological Diagnostic Unit Public Health Laboratory and Victorian Infectious Diseases Reference Laboratory, The Peter Doherty Institute for Infection & Immunity
Microbiological Diagnostic Unit Public Health Laboratory and Victorian Infectious Diseases Reference Laboratory, The Peter Doherty Institute for Infection and Immunity
Microbiological Laboratory,Lu`an CDC
Microbiology
Microbiology & Immunology, University of North Carolina
Microbiology and Immunology department, Pasteur institute in Ho Chi Minh city, Pasteur Street, Ho Chi Minh, Ho Chi Minh 72408, Viet Nam
Microbiology and Immunology, University of South Alabama
Microbiology and Immunology, University of North Carolina, 450 West Drive, Chapel Hill, NC 27514, USA
Microbiology and Virology Laboratory, 'Policlinico Riuniti, Azienda Ospedaliero Universitaria, Foggia'
Microbiology and Virology Laboratory, Policlinico Riuniti, Azienda Ospedaliero Universitaria, Foggia
Department of Molecular Medicine, Computational Medicine Group, Univeresity of Padova, Padova, Italy
Microbiology and Virology Unit, Florence Careggi University Hospital
Department of Molecular Medicine,Computational Medicine Group,Univeresity of Padova,Padova,Italy
Microbiology Department - University Hospital Brussel
Microbiology Department & Molecular Biology CORE | CDB | Hospital Clinic | Barcelona
Microbiology Department, University Hospital Donostia
Phylogenomics Lab, CINBIO, Universidade de Vigo
Microbiology Detection Department, Hangzhou CDC, Mingshi Road 568, Hangzhou, Zhejiang 310021, China
Microbiology Detection, Hangzhou CDC
Microbiology Division, SC DHEC
Microbiology Division, South Carolina Department of Health and Environmental Control
Microbiology Division, South Carolina Department of Health and Environmental Control (SC DHEC)
Microbiology Division, SC DHEC
Microbiology Division, South Carolina Department of Health and Environmental Control
Microbiology Division, South Carolina Department of Health and Environmental Control Public Health Laboratory (SC DHEC PHL)
NBCC Sequencing Facility
Microbiology Laboratory,Lu`an CDC
Microbiology Service, Hospital Universitario Clinico San Cecilio, Granada
Genomes & Disease, Center for Research in Molecular Medicine and Chronic Diseases, University of Santiago de Compostela
Microbiology Unit ASST Grande Ospedale Metropolitano Niguarda, Milan, Italy
SEA Microbiome Unit, Faculty of Industrial Sciences & Technology, Universiti Malaysia Pahang
Microbiology, Babylon university college of medicine
Microbiology, Bangalore Medical College & Research Institute
Microbiology, University of Pennsylvania
Microbiology, Infectious Diseases and Immunology, Centre de Recherche du Centre Hospitalier de l'Universite de Montreal
Microbiology, Regional Medical Research Centre (ICMR), Dr. B Borkakoty, Scientist-E, Regional Medical Research Centre For NE Region (ICMR), Bokel, Dibrugarh, Assam 786001, India
Microbiology, The University of Hong Kong, Block T Queen Mary Hospital Pok Fu Lam Road, Hong Kong, China
Microbiology, Virology and Biemergency Laboratory-ASST FBF Sacco
Microvida
Mikrobiologie, RARI
Ministry of Health
Ministry Of Health- labs
Virology Unit, Department of Microbiology, Faculty of Medicine, Kuwait University
Ministry Of Health - Central Labs
Virology Unit, Department of Microbiology, Faulty of Medicine, Kuwait University
Biomedical Research Center (BRC), Qatar University / Qatar Genome Project (QGP)
Weill Cornell Medical College - Qatar (WCM-Q), Genomics Core Laboratory / Qatar Genome Project (QGP)
Missouri State Public Health Laboratory
Miyazaki Prefectural Institute for Public Health and Environment

MGZ Medical Genetics Center
Moderna Inc.
MOH - Jaber Al-Ahmad Hospital (Innovation Research Laboratory)
MOH - Jordan
Transcriptomics & Applied Genomics (TAG)
Molecular & Genomic Pathology Laboratory, Thomas Jefferson University Hospital
Molecular Biology and Virology lab, Faculty of Veterinary Medicine, Jordan University of Science and Technology
Molecular biology division, Institute of Clinical Biochemistry and Diagnostics, Charles University, Faculty of Medicine in Hradec Krlov and University Hospital Hradec Krlov
Eijkman Institute for Molecular Biology, National Research and Innovation Agency; Faculty Medicine and Health Sciences, Warmadewa University
Genomics Core Laboratory, New York Medical College
Molecular Biology, IZS Sicilia
Molecular diagnostic unit for viral haemorrhagic fevers and emerging viruses, Bouak CHU Laboratory
Project group Epidemology of Highly Pathogenic Microorganisms, Robert Koch-Institute
Molecular diagnostic unit for viral haemorrhagic fevers and emerging viruses, Bouake CHU Laboratory
Molecular Diagnostics Mater Dei Hospital
Molecular Diagnostics Pathology Department Mater Dei Hospital
Molecular Diagnostics Pathology Department Mater Dei Hospital Malta
Molecular Diagnostics Pathology Department Mater Dei Hospital MAlta
Molecular Genetic, Immuno Gene Center, Doctor stree, Tallat Bulding No. 19, Erbil 44001, Iraq
Molecular Hepatology Laboratory AOU Messina
Molecular Hepatology Laboratory AOU Messina (Italy)
Molecular Infectious Disease
Molecular Infectious Disease, Quest Diagnostics, 33608 Ortega Hwy, San Juan Capistrano, CA 92675, USA
Molecular Microbiology & Immunology, University of Missouri
Molecular Microbiology, Washington University of St. Louis
Molecular Pathology Division, Department of Pathology, Hong Kong Sanatorium & Hospital
Igenbio, Inc.
Molecular Pathology, University of Illinois at Chicago
Molecular Pathology, Mehr Pathobiology Lab
Molecular Pathology, Mehr Pathobiology Lab, Emam, Hashtgerd, Alborz 3361766538, Iran
Molecular Pathology, Pathology and Laboratory Medicine Institute, Cleveland Clinic, Ohio, USA
Molecular Pathology, University of Illinois at Chicago
Molecular Virology Unit, Microbiology and Virology Depertment, Fondazione IRCCS Policlinico San Matteo, Pavia
Universidade Federal do Parana (UFPR)
Montana Public Health Laboratory
Albert Einstein College of Medicine, Dept. of Microbiology & Immunology, Chandran lab
Monterey County Public Health Laboratory
Microbial Genomics lab LAU
Microbial Genomics Lab LAU Byblos
Microbial genomics lab LAU
Microbial Genomics Lab LAU
MPHL
MRC-University of Glasgow Centre for Virus Research
MRC/UVRI & LSHTM Uganda Research Unit, Uganda Virus Research Institute
Where sequence data have been generated and submitted to GISAID
MRC/UVRI & LSHTM UgandaResearch Unit , Rakai Health Sciences Program
MRC/UVRI & LSHTM UgandaResearch Unit, Central Public Health Laboratories
MRC/UVRI & LSHTM UgandaResearch Unit, Rakai Health Sciences Program
LBV Le Dantec
MRCG at LSHTM Genomics lab
MS PHL
University of Mississippi Medical Center, Molecular and Genomics Core Facility
MSK Microbiology Lab
Virology Unit, Department of Microbiology, Faculty of Medicine
Virology Unit, Department of Microbiology, Faculty of Medicine, Kuwait
Virology Unit, Department of Virology, Faculty of Medicine, Kuwait University
Virology Unit, Microbiology Department, Faculty of Medicine, Kuwait University
Multidisciplinary Research Unit, Institute of Medical Sciences, Banaras Hindu University, Varanasi - 221005

Multidisciplinary Research Unit, Institute of Medical Sciences, Banaras Hindu University, Varanasi -221005
Multiplex DX
MUSC Molecular Pathology Laboratory
MVZ Laborrzte Singen
Nagasaki Prefectural Institute of Environment and Public Health
Forschungszentrum Borstel
NAMRU-6
Nara Prefecture Institute of Health
National Center for Infectious and Parasitic Diseases
National Center for Infectious and Parasitic Diseases (NCIPD)
National Center for Infectious and Parasitic Diseases(NCIPD)
Institute of Molecular Biology NAS RA, Republic of Armenia, Department of Bioengineering, BioinformaticsInstitute and Molecular Biology IBMPh RAU, Republic of Armenia
National Center for Expertise, Kazakhstan National Center for Biotechnology, Kazakhstan
National Center for Expertise, National Center for Biotechnology, Kazakhstan
National Center of Infectious and Parasitic Diseases
National Centre For Cell Science INSACOG
National Institute of Virology, Pune
National Centre for Communicable Disease (NCCD) National Influenza Center
National Centre for Communication Disease (NCCD) National Influenza Center
NCDC/CSIR-IGIB
Programme in Emerging Infectious Diseases, Duke-NUS Medical School
National Centre for Infectious Diseases, National Centre for Infectious Diseases
National Genomics Core- Center for DNA Fingerprinting and Diagnostics (NGC-CDFD)- DBT's PAN-INDIA-1000 Genome consortium
Botswana Institute for Technology Research and Innovation
Botswana Institute for Technology Research and innovation
Division of Medical Virology, Stellenbosch University and National Health Laboratory Service (NHLS)
Stellenbosch University and NHLS
National Health Laboratory Services
National Health Laboratory Services, Virology
National Hematology and Transfusiology Center, Department of Medical Genetics
Oxford University Clinical Research Unit, Hanoi, Vietnam
National Influenza and other Respiratory Viruses Centre-Tunisia
National Influenza and other Respiratory Viruses Centre-Tunisia, Virology Unit, Microbiology Laboratory, Charles Nicolle Hospital, Boulevard 9 Avril, Tunis 1006, Tunisia
National Influenza and other Respiratory Viruses Centre-Tunisia,Virology Unit, Microbiology Laboratory, Charles Nicolle Hospital, Boulevard 9 Avril, Tunis, Tunis 1006, Tunisia
National Influenza Center - National Institute of Hygiene and Epidemiology (NIHE)
National Influenza Center (NIC). Virology department. Institut National d'Hygine.
National Influenza Center, Bahrain
National Influenza Center, National Institute of Hygiene and Epidemiology (NIHE)
National Institute of Hygiene and Epidemiology (NIHE)
National Influenza Center
National Influenza Centre
National Influenza Centre for Northern Greece
National Influenza Centre for Nothern Greece
The University of Hong Kong
National Institute for Allergy and Infectious Diseases Integrated Research Facility - Frederick (NIAID IRF- Frederick), National Institutes of Health (NIH)
National Institute for Biological Standards and Control
National Institute for Communicable Disease Control and Prevention (ICDC) Chinese CDC (China CDC)
National Institute for Infectious Diseases, INMI, 'L. Spallanzani' IRCCS
National Institute for Viral Disease Control & Prevention, CCDC
National Institute for Viral Disease Control & Prevention, China CDC
Institute of Viral Disease Control and Prevention, China CDC
National Institute for Viral Disease Control and Prevention, China CDC, Yunnan Provincial CDC
National Institute for Viral Disease Control and Prevention, China CDC, Beijing, China
Bioinformatics Division, National Institute of Biotechnology
National Institute of Biotechnology
Genomics Lab NIBD
National Key Laboratory of Gene Technology, Institute of Biotechnology (IBT)

National Institute of Infectious Diseases (NIID)
Dr. Qudrat-I-Khuda Road, Dhaka-1205, Bangladesh
Genomic Research Lab, BCSIR, Dr. Qudrat-E-Khuda Road, Dhaka-1205, Bangladesh
Genomic Research Lab, BCSIR, Dr. Qudrat-E-Khuda Road, Dhaka 1205, Bangladesh
Genomic Research Lab. BCSIR
Sher-e-Bangla Nagar, Agargaon, Dhaka-1207, Bangladesh.
GenExpress GmbH
Genomic Research Laboratory, Bangabandhu Sheikh Mujib Medical University
Genomic Research Laboratory, Bangabandhu Sheikh Mujib Medical University Shahbag, Dhaka_1000
National Institute of Public Health
MRC/UVRI & LSHTM Uganda Research Unit, National Institute of Public Health
National Institute of Virology, NIV Influenza
National Key Laboratory of Gene Technology, Institute of Biotechnology, Vietnam Academy of Science and Technology
CISLD (Clinical Institute of Special Laboratory Diagnostics), University Children's Hospital, University Medical Center Ljubljana
National Laboratory for Health, Environment and Food
National Laboratory for Health, Environment and Food;Institute of Microbiology and Immunology
NLZOH (National Laboratory for Health, Environment and Food) / CISLD (Clinical Institute of Special Laboratory Diagnostics), University Children's Hospital, University Medical Center Ljubljana
National Medical Research Center for Obstetrics, Gynecology and Perinatology named after Academician V.I.Kulakov of the Ministry of Healthcare of Russian Federation
DNA Sequencing and Synthesis Facility (oligo.pl), Institute of Biochemistry and Biophysics PAS
National Plateform bis UMONS/Jolimont
National Public Health Center, National Biosafety Laboratory
Center for Infection and Immunity, Columbia University
National Public Health Laboratory
African Centre of Excellence for Genomics of Infectious Diseases (ACEGID), Redeemer's University
African Centre of Excellence for Genomics of Infectious Diseases, Redeemers University
National Virology Reference Laboratory
National Public Health Laboratory, National Centre for Infectious Diseases
National Public Health Organization
MB1554
National Reference Center for Viruses of Respiratory Infections, Institut Pasteur, Paris, France
National Reference Laboratory
National Reference Laboratory for COVID-19, Pasteur Institute of Iran
NRL-HIV
National reference Laboratory, NCDC, Gaduwa, Abuja
National Reference Laboratory, NCDC, Gaduwa, Abuja, Nigeria
National Reference Laboratory, Nigeria Centre for Disease Control, Gaduwa, Abuja, Nigeria
National Reference Laboratory, Nigeria Centre for Disease Control, Gaduwa,Abuja, Nigeria
National Research Center for Translational Medicine (Shanghai), Ruijin Hospital affiliated to Shanghai Jiao Tong University School of Medicine & Shanghai Public Health Clinical Center
Kazakh National Agrarian University (KazNAU) TreeGene LLP Genetic Laboratory
National University of Sciences and Technology (NUST), Islamabad
National Veterinary Institute
Irish Coronavirus Sequencing Consortium - Helixworks
Irish Coronavirus Sequencing Consortium - Teagasc Moorepark
Irish Coronavirus Sequencing Consortium - Teagasc Oakpark
Computational Virology Group, Center for Bacteria and Viruses Resources and Bioinformation, Wuhan Institute of Virology, Chinese Academy of SciencesWuhan 430071, China
James Molecular Lab
Natonal Public Health Laboratory
Naval Medical Research Center Biological Defense Research Directorate
New Brunswick - Vitalit Health Network
New Brunswick Vitalit Health Network
NCDC Institute of Genomics and Integrative Biology
NCDC/IGIB
NCSLPH
UNMC COVID-19 Response Team
Nebraska Public Health Laboratory COVID-19 Response Team
NPHL COVID-19 Response Team

NPHL
Massive Bioinformatics ARGE Technologies Company
Neurovirology, National Institute of Mental Health and Neurosciences, Hosur Road, Bangalore, Karnataka 560029, India
Nevada State Health Lab
New England Biolabs
New Jersey Public Health and Environmental Laboratories
UFS Virology
NIAID, Laboratory of Parasitic Diseases
Virology Departement
International Centre for Genetic Engineering and Biotechnology (ICGEB) and ARGO Open Lab Platform for Genome Sequencing
National Reference Laboratory, NCDC, Gaduwa, Abuja
National Reference Laboratory, NCDC, Abuja
African Centre of Excellence for Genomics of Infectious Diseases (ACEGID), Redeemers University
African Centre of Excellence for Genomics of Infectious Diseases (ACEGID), Redeemers University, Ede
Niigata Prefectural Institute of Public Health and Environmental Sciences
Genomic Research Laboratory, Bangabandhu Sheikh Mujib Medical University Bangabandhu Sheikh Mujib Medical University (BSMMU) Shahbag, Dhaka_1000
Genomic Research Laboratory, BSMMU Bangabandhu Sheikh Mujib Medical University (BSMMU) i
2. Tricity SARS-CoV-2 sequencing consortium: University of Gdansk, Medical University of Gdansk, Vaxican Ltd., Invicta Ltd. 2. National Institute of Public Health - National Institute of Hygiene, Warsaw, Poland
New Jersey Public Health and Environmental Laboratories (NJ-PHEL)
New Jersey Public Health and Environmental Laboratories (NJPHEL)
NJ PHEL
NJ_PHEL Public Health and Environmental Laboratories
Newfoundland and Labrador - Eastern Health
Department for Public Health Microbiology Ljubljana, NLZOH
NLZOH, Laboratory for Virology
Noblis
North Carolina State Laboratory of Public Health
Norwegian Institute of Public Health
Oslo Helse
Norwegian Institute of Public Health, Department of Virology
Notre Dame Genomics & Bioinformatics Core Facility
NSU Genome Research Institute (NGRI)
NSU Genome Research Institute (NGRI), North South University
NSW Health Pathology - Institute of Clinical Pathology and Medical Research; Centre for Infectious Diseases and Microbiology Laboratory Services; Westmead Hospital; University of Sydney
Clinical microbiology, Sahlgrenska University Hospital
Clinical Microbiology, Sahlgrenska University Hospital,
MEBLAB
Botswana Harvard HIV Reference Laboratory,
WSSE WARSZAWA
BioInfo Experts, LLC
Bioinfoexperts
BioInfoExperts
Ohio Department of Health
Ohio Department of Health Laboratories
Ohio Depart of Health Laboratory
Ohio Department of Health laboratory
Polaris Molecular Laboratory
James Polaris Molecular Laboratory
OHSU-MM Lab
Ginkgo Bioworks Clinical Laboratory
Ginkgo BioworksClinical Laboratory
Oregon SARS-CoV-2 Genome Sequencing Center
OHSU Molecular Microbiology Laboratory
Oita Prefectural Institute of Health and Environment
Oklahoma Animal Disease Diagnostic Laboratory
Frana Lab
CEITEC MU

Biotechnology & OMICs Laboratory
Biotechnology & OMICs Laboratory, Natural & Medical Sciences Research Center, University of Nizwa
Department of Microbiology and Immunology-SQUH Department of Microbiology and Immunology, Sultan Qaboos University Hopsital, P.O 35, Postal code 123
Oman-National Influenza Center
Department ofMicrobiologyand Immunology- SQUH
Department ofMicrobiologyand Immunology-SQUH
Microbiology laboratory- Sultan Qaboos University Hospital
Oman-NIC
Department ofMicrobiologyand Immunology
Omega Diagnostics
omega Diagnostics at Mounes
Omega Diagnostics at Mounes
Omega Diagnostics st Mounes
Omega Diganostics at Mounes
Onega Diagnostics at Mounes
Omics Sciences Lab
Omics Sciences Laborator
Onderzoeksgroep Virologie
Ontario Agency for Health Protection and Promotion (OAHPP)
Operation Infectious Diseases, Naval Health Research Center
Orange County Public Health Laboratory
Orebro University Hospital
Public Health Wales Microbiology Cardiff Wales Specialist Virology Centre
Orion Laboratories
Clinical Laboratory Medicine, Kyoto University Graduate School of Medicine
U.O. Genomica - Istituto Zooprofilattico Sperimentale del Piemonte, Liguria e Valle d'Aosta
ATS SARDEGNA - ASSL Nuoro, Ospedale San Francesco, Laboratorio COVID e Laboratorio Specialistico UOC Ematologia
OUCRU
OUCRU/HTD
Oxford University Clinical Research Unit (OUCRU)
Oxford University Clinical Research Unit, Hanoi, Vietnam
Institute of Genomics and Integrative Biology - Council of Scientific and Industrial Research
P.O.CARDARELLI
Molecular Genetics Lab
Pamukkale University Department of Medical Genetics
Pandemic Response Lab, R&D
Lacen-PR
LACEN PR
Lacen/PR
Parana
Pascale
Department of Microbiology and Immunology - Pasteur Institute in Ho Chi Minh city
Pasteur Institute of Iran
Rapid Response Team
PathCare, Cape Town
Pathogen Discovery, Respiratory Viruses Branch, Division of Viral Diseases, Centers for Dieases Control and Prevention
Pathogen Discovery, Respiratory Viruses Branch, Division of Viral Diseases, Centers for Dieases Control and Prevention
Pathogen Genomics Lab King Abdullah University of Science and Technology (KAUST)
Pathogen Genomics Lab King Abdullah University of Science and Technology(KAUST)
Pathogen Sequencing Lab, National Institute for Biomedical Research (INRB)
Pathogen Sequencing Lab, National Institute for Biomedical Research (INRB)
Pathogenic microbiology laboratoryHuashan Hospital, Fudan University
Pathology and Laboratory Medicine Institute, Cleveland Clinic, Ohio, USA
CIDM-PH et al.
Pathology, Sidra Medicine
Pathology, UAB
Pathology, University of Alabama at Birmingham
Pathology, UAB

Pathology, University of Alabama at Birmingham
PathWest Laboratory Medicine WA
PathWest Laboratory Medicine WA Microbial Surveillance Unit
PCR Laboratory, The First Affiliated Hospital of Zhengzhou University, Zhengzhou, Henan, China
Pennsylvania Department of Health Bureau of Laboratories
ICMR-NATIONAL INSTITUTE OF VIROLOGY, MICROBIAL CONTAINMENT COMPLEX
Philippine Genome Center, University of the Philippines System
Plataforma de Vigilancia Molecular (PVM) - FIOCRUZ/BA
Plateforme Clinique de testing Namuroise
Arlon
UZA, Clinical Biology
Population Medicine and Diagnostic Sciences, Cornell University
Population Medicine and Diagnostic Sciences, Cornell University
Thai Red Cross Emerging Infectious Diseases Clinical Center and Faculty of Medicine, Chulalongkorn University
Animal Disease Diagnostic Laboratory Purdue University College of Veterinary Medicine
BioInfoExperts LLC
ASM - P.O. Madonna delle Grazie - Matera
PRIMBIO RESEARCH INSTITUTE,LLC
Alanagreh
Center of Genomics and bioinformatics, Bioinformatics laboratory
Pro-Vitam Diagnostics and Research Laboratory
Prof. Massimo Zollo CEINGE TASK-FORCE COVID19 - Regione Campania
Programa de Oncovirologia, Instituto Nacional de Cncer
Programme in Emerging Infectious Diseases, Duke-NUS Medical School
Protzer Lab, Gagneur Lab, Robert Koch Institut
Protzer Lab, Institut fr Medizinische Mikrobiologie und Hygiene, Gagneur Lab
Protzer Lab
Providence St. Joseph Health Molecular Genomics Laboratory
Provincial Public Health Reference Laboratory
Provincial Public Health Reference Laboratory (PPHRL)
ProvincialPublicHealthReferenceLaboratory(PPHRL)
ProvincialPublicHealthReferenceLaboratory(PPHRL)P&SHD
Public Health Agency of Canada - National Microbiology Laboratory
Public Health Authority of the Slovak Republic
Faculty of Natural Sciences, Comenius University in Bratislava
Public Health Laboratory, Saudi CDC
Public Health Laboratory - Infectious Disease Lab, Minnesota Department of Health Infectious Disease Laboratory Submission Group
Public Health Laboratory - Infectious Disease Lab, Minnesota Department of Health Infectious Disease Laboratory Submission Group
Public Health Laboratory, Minnesota Department of Health, 601 Robert St N, Saint Paul, MN 55164, USA
Public Health Ontario Laboratories
Public Health Ontario Laboratory
Public Health Virology-Forensic and Scientific Services (PHV-FSS)
Public Health Virology Laboratory, Forensic and Scientific Services, Queensland Health
Public Health Virology Laboratory, Forensics and Scientific Services, Queensland Health
Public Health Virology, Queensland Health Forensic and Scientific Services
Public Health Wales Microbiology Cardiff
West Java Health Laboratory
Genetics Working Group (Pokja Genetik) Faculty of Medicine, Public Health and Nursing Universitas Gadjah Mada (FK-KMK UGM); Disease Investigation Center Wates Ministry of Agriculture Indonesia; Department of Microbiology FK-KMK UGM; Laboratorium Diagnostik Yayasan Tahija World Mosquito Program (WMP) Yogyakarta Center for Tropical Medicine FK-KMK UGM; Integrated Research Center FK-KMK UGM; Department of Computer Science and Electronics FMIPA UGM; Balai Besar Teknik Kesehatan Lingkungan dan Pengendalian Penyakit (BBTKLPP) Yogyakarta
Universitas Sebelas Maret (UNS); Rumah Sakit UNS (RS-UNS); National Institute of Health Research and Development, Indonesian Ministry of Health
University of Sarajevo, Veterinary Faculty, Laboratory for Molecular Diagnostic and Research Laboratory
QIB, Quadram Institute Bioscience, Norwich Research Park, Norwich NR4 7UQ, United Kingdom
QUALITY CONTROL CHEMICAL BIOLOGICAL RISK, AOOR Villa Sofia Cervello Palermo
Hong Kong Children's Hospital
Queensland Health Forensic and Scientific Services
Melbourne Diagnostic Unit Public Health Laboratory (MDU-PHL)
Victorian Infectious Diseases Reference Laboratory (VIDRL) and the Melbourne Diagnostic Unit Public Health Laboratory (MDU-PHL)

Q Squared Solutions - QRTP facility

Quest Diagnostics

R. G. Lugar Center for Public Health Research, National Center for Disease Control and Public Health (NCDC) of Georgia.

Radboudumc

Microbial Pathogenomics Lab

Rafik Hariri University Hospital

MOA Lab

Rami Kantor lab

Rare and Infectious Disease Lab, Institute of Biomedical and gentic Engineering

Rare and Infectious Disease Lab, Institute of Biomedical and Gentic Engineering

Reditus Laboratories

Regional Hospital Liberec

Regional Science and Technology Center

Regional VRDL, ICMR-RMRC

Regional VRDL, Indian Council Of Medical Research-Regional Medical Research center (ICMR-RMRC)

Regional VRDL,Dibrugarh

Immunogenomics group, Institute of Life Sciences, Bhubaneswar

State Key Laboratory of Agriculture Microbiology, Huazhong Agric

Research and Medical Analysis Laboratory of Gendarmerie Royale

Research institute for Biotechnology and Bio-engineering, Isfahan University of Technology

Research Institute for Tropical Medicine, Molecular Biology Laboratory

Research platform for Transfusion-transmitted Disease, Institute of Blood Transfusion, Chinese Academy of Medical Sciences

Cicin Sain lab, Helmholtz Centre for Infection Research

University of Zagreb Centre for research and knowledge transfer in biotechnology

Respiratory virus Laboratory, Chinese Academy of Medical Science

Respiratory Viruses Branch, CDC

Respiratory Viruses Branch, US CDC

Respiratory Viruses Branch, CDC

Respiratory Viruses Branch, US CDC

Respiratory Viruses Unit, Microbiology Department, Vall d'Hebron University Hospital, Passeig Vall d'Hebron 119-129, Barcelona, Catalonia 08035, Spain

Rhode Island State Health Laboratories

Rhode Island State Health Laboratory

Laboratorio de Biologia Molecular Hemocentro de Ribeirao Preto

Ribeirao Preto

Laboratorio de Biologia Molecular Hemocentro de Ribeirao Preto SP

Robert Koch Institut

Robert Koch Institute, Bioinformatics MF1, Berlin, Germany

Robert Koch Institute, ZBS1 Highly Pathogenic Viruses & Bioinformatics MF1, Berlin, Germany

Multidrug-Resistant Organism Repository and Surveillance Network (MRSN)

Rocky Mountain Laboratories

Molecular Virology Laboratory of Oswaldo Cruz Foundation of Rondnia

Royal GD

Royal Hobart Hospital

Indonesian Institute of Sciences (LIPI)

National Research and Innovation Agency (BRIN-Indonesia, VenomCoV Project Team); West Java Health Laboratory

Genetics Working Group (Pokja Genetik) Faculty of Medicine, Public Health and Nursing Universitas Gadjah Mada (FK-KMK UGM); Disease Investigation Center Wates Ministry of Agriculture Indonesia; Department of Microbiology FK-KMK UGM; Laboratorium Diagnostik Yayasan Tahija World Mosquito Program (WMP) Yogyakarta Center for Tropical Medicine FK-KMK UGM; Integrated Research Center FK-KMK UGM; Department of Computer Science and Electronics

Genetics Working Group (Pokja Genetik) Faculty of Medicine, Public Health and Nursing Universitas Gadjah Mada (FK-KMK UGM); Disease Investigation Center Wates Ministry of Agriculture Indonesia; Department of Microbiology FK-KMK UGM; Laboratorium Diagnostik Yayasan Tahija World Mosquito Program (WMP) Yogyakarta Center for Tropical Medicine FK-KMK UGM; Integrated Research Center FK-KMK UGM; Department of Computer Science and Electronics FMIPA UGM

UNS-RS UNS

UNS/RS-UNS, Faculty of Medicine

UNS-RS UNS, Surakarta

Universitas Sebelas Maret (UNS); Rumah Sakit Universitas Sebelas Maret (RS-UNS), Surakarta; National Institute of Health Research and Development, Indonesian Ministry of Health , Jakarta).

Genetics Working Group (Pokja Genetik) Faculty of Medicine, Public Health and Nursing Universitas Gadjah Mada (FK-KMK UGM), Disease Investigation Center Wates Ministry of Agriculture Indonesia, Department of Microbiology FK-KMK UGM, Laboratorium Diagnostik Yayasan Tahija World Mosquito Program (WMP) Yogyakarta Center for Tropical Medicine FK-KMK UGM, Integrated Research Center FK-KMK UGM, Department of Computer Science and Electronics FMIPA UGM

Genetics Working Group (Pokja Genetik) Faculty of Medicine, Public Health and Nursing Universitas Gadjah Mada (FK-KMK UGM); Disease Investigation Center Wates Ministry of Agriculture Indonesia; Department of Microbiology FK-KMK UGM; Laboratorium Diagnostik Yayasan Tahija World Mosquito Program (WMP) Yogyakarta Center for Tropical Medicine FK-KMK UGM; Integrated Research Center FK-KMK UGM; Department of Computer

Stem Cell Lab, Universitas Pembangunan Nasional Veteran Jakarta

Stem Cell Lab, Universitas Pembangunan Nasional Veteran Jakarta (UPNVJ)

Stem Cell Lab. Universitas Pembangunan Nasional Veteran Jakarta (UPNVJ)

Eijkman Institute for Molecular Biology, National Research and Innovation Agency; Raden Mattaher Regional General Hospital

Genetics Working Group (Pokja Genetik) Faculty of Medicine, Public Health and Nursing Universitas Gadjah Mada (FK-KMK UGM); Disease Investigation Center Wates Ministry of Agriculture Indonesia; Department of Microbiology FK-KMK UGM; Laboratorium Diagnostik Yayasan Tahija World Mosquito Program

Mochtar Riady Institute for Nanotechnology-Universitas Pelita Harapan; Eijkman Institute for Molecular Biology, National Research and Innovation Agency

Universitas Sebelas Maret (UNS); Rumah Sakit Universitas Sebelas Maret (RS-UNS), Surakarta; National Institute of Health Research and Development, Indonesian Ministry of Health, Jakarta.

Russian Academy of Sciences, Federal Research Center for Virology and Microbiology

Rwanda National Laboratory

Rwanda National Reference Laboratory

Ryota Kumagai Tokyo Metropolitan Institute of Public Health

Ryota Kumagai Tokyo Metropolitan Institute of Public Health

Laboratorio Biologia Molecolare Sars Cov2 - UOC Laboratorio Analisi - Servizio Medicina di Laboratorio, Ospedale 'San Francesco' - ATS-ASSL Nuoro and Laboratorio specialistico UOC Ematologia - Ospedale 'San Francesco' - ATS-ASSL Nuoro

S.S. Genetica e Tecniche Omiche Avanzate Istituto Zooprofilattico Sperimentale del Piemonte, Liguria e Valle d'Aosta

S.M.S.Medical College, Jaipur, Rajasthan

SA Pathology

VPRL

Saitama City Institute of Health Science and Research

Saitama Institute of Public Health

Saitama Medical University

Saitama Prefectural Institute of Public Health

Salzkammergutklinikum Vcklabruck, Institut fr Pathologie

San Diego County Public Health Lab

San Diego County Public Health Laboratory

San Gallicano Dermatological Institute I.F.O.

Sanquin B.V.

Laboratorium Kesehatan Provinsi Jawa Barat; School of Life Sciences and Technology & School of Pharmacy-Institut Teknologi Bandung; Molecular Genetics Laboratory-Faculty of Medicine-Universitas Padjadjaran

Laboratorium Kesehatan Provinsi Jawa Barat; Molecular Genetics Laboratory-Faculty of Medicine-Universitas Padjadjaran; School of Life Sciences and Technology & School of Pharmacy-Institut Teknologi Bandung

SARS-CoV-2 Sequencing Castilla y Leon-Spain Consortium

ARGO Laboratorio Genomica ed Epigenomica

SC (UCO) Igiene e Sanit Pubblica, ASUGI, Trieste

SC Department of Health and Environmental Control

SC Microbiologia e Virologia AOUSS

School of Pharmacy

St. Jude Children's Research Hospital Infectious Diseases

School of Pharmacy, Shenandoah University

School of Pharmacy, Shenandoah University

School of Public Health, The University of Hong Kong, 21 Sassoon Road, Pokfulam, Hong Kong

PathoSense BV

Kazakh National Agrarian Research University (KazNARU), TreeGene LLP Genetic Laboratory

Kazakh National Agrarian University (KazNAU); TreeGene LLP Genetic Laboratory; National Scientific Center for Especially Dangerous Infections (NSCEDI)

Scientific Department, Division of Molecular Biology

Veterinary Specialized Institute Kraljevo, Serbia

Seattle Flu Study

Second Hospital of Anhui Medical University

Section of Microbiology Department of Molecular and Translational Medicine, University of Brescia

Sendai City Institute of Public Health

Sequencing and Bioinformatics Center

Sequencing and Bioinformatics Service & Molecular Epidemiology Laboratory, FISABIO-Public Health, Av.Catalunya, 21, Valencia, Valencia 46020, Spain

Sequencing and Bioinformatics Service and Molecular Epidemiology Research Group. FISABIO-Public Health
Sequencing and Bioinformatics Service and Molecular Epidemiology Research Group. FISABIO-Public Health
Sequencing and Bioinformatics Service FISABIO-Public Health
Sequencing Core Facility, National Institute for Communicable Diseases, 1 Modderfontein Road, Johannesburg, Gauteng 2131, South Africa
Hemocentro de Ribeirao Preto
Laboratrio de Biologia Molecular - Hemocentro de Ribeiro Preto
Laboratory Cell Biology of Viral Infection-INSERM unit 944
Laboratory of Cell Biology of viral infection, Unit INSERM-U944
Laboratoire de Virologie
Servicio de Microbiologa Hospital Ramn y Cajal
SeqCOVID-SPAIN consortium/IBV (CSIC)
Servicio de Microbiologia Hospital Ramon y Cajal
Sequencing and Bioinformatics Service and Molecular Epidemiology Research Group. FISABIO-Public Health, and SeqCOVID-Spain Consortium
Servicio Microbiologa Hospital La Paz
Sequencing and Bioinformatics Service and Molecular Epidemiology Research Group. FISABIO-Public Health.
Instituto Nacional Enfermedades Infecciosas C.G.Malbran
Servizo de Microbioloxa. Complexo Hospitalario de Santiago de Compostela
Servizo de Microbioloxa. Complexo Hospitalario de Santiago de Compostela21
Servizo de Microbioloxa. Complexo Hospitalario de Santiago de Compostela.
Servizo de Microbioloxa. Complexo Hospitalario Universitario de Santiago de Compostela
Ruer Bokovic Institute; Forensic Science Centre Ivan Vueti; University of Zagreb Faculty of Science
Shamir Medical Center (Asaf Harofe)
Shanghai Public Health Clinical Center & School of Public Health, Fudan University, Shanghai, China
Department of Pathology and Laboratory Medicine, University of California Los Angeles
Zhejiang Provincial CDC
Molecular Surveillance lab Sheikh Khalifa Medical City
Shenzhen Key Laboratory of Pathogen and Immunity, National Clinical Research Center for Infectious Disease, Shenzhen Third People's Hospital
Shiga Prefectural Institute of Public Health
Shimane Prefectural Institute of Public Health and Environmental Science
Shizuoka City Institute of Environmental Sciences and Public Health
Japan COVID-19 Open Data Consortium
Showa University PCR Center
Mochtar Riady Institute for Nanotechnology-Universitas Pelita Harapan
Mochtar Riady Institute for Nanotechnology, Universitas Pelita Harapan
Singapore General Hospital
siParadigm LLC
Saskatchewan - Roy Romanow Provincial Laboratory (RRPL)
SMS Medical College Jaipur
SMS Medical College jaipur
S.M.S. Medical College
SMS Medical College
SMS, Medical college
SMS, Medical College, Jaipur
MRC/UVRI & LSHTM Uganda Research Unit, South Sudan Ministry of Health, WHO South Sudan
Southern Nevada Public Health Laboratory
Special Operations Medical Research Division, Defence Services Medical Research Centre
Specialized Lab for COVID-19 Detection, Department of Genetic Engineering and Biotechnology
Spiez Laboratory
SRO AG
SRC VB Vector, Collection of microorganisms Department.
ELDA biotech
St.Vincent's University Hospital
Clinical virology Laboratory, Stanford University School of Medicine
Clinical Virology Laboratory, Stanford University School or Medicine
Stanford University School of Medicine, Clinical Virology Laboratory
State Hygienic Laboratory at the University of Iowa
Robert Koch Institute, ZBS1 Highly Pathogenic Viruses, Berlin, Germany
State Key Laboratory for Diagnosis and Treatment of Infectious Diseases, National Clinical Research Center for Infectious Diseases, First Affiliated Hospital, Zhejiang University School of Medicine, Hangzhou, China 310003

State Key Laboratory for Diagnosis and Treatment of Infectious Diseases, National Clinical Research Center for Infectious Diseases, First Affiliated Hospital, Zhejiang University School of Medicine, Hangzhou, China. 310003

State Key Laboratory for Emerging Infectious Diseases Department of Microbiology Li Ka Shing Faculty of Medicine The University of Hong Kong

State Key Laboratory of Biotherapy of Sichuan University

State Key Laboratory of Emerging Infectious Diseases, The University of Hong Kong

State Key Laboratory of Pathogen and Biosecurity, Beijing Institute of Microbiology and Epidemiology

State Key Laboratory of Respiratory Disease, National Clinical Research Center for Respiratory Disease, Guangzhou Institute of Respiratory Health, the First Affiliated Hospital of Guangzhou Medical University, National Clinical Research Center for Respiratory Disease, No. 195 Dongfeng Xi road, Yuexiu district, Guangzhou, China 510182, China

State Key Laboratory of Virology, Wuhan University

State Laboratories Division, Hawaii State Department of Health

State Research Center of Virology and Biotechnology VECTOR, Department of Collection of Microorganisms

Statens Serum Institute

Stefan S. NicolauInstitute of Virolog

285 Mihai Bravu Ave, Bucharest, Romania

SMU Metagenomics lab

Ribeiro Preto - SP

Susan Baker LAB

Unilabs Eskilstuna Sweden

Institute for Systems Biology

Eijkman Institute for Molecular Biology, National Research and Innovation Agency; Syiah Kuala University

Department of Microbiology, Institute of Biomedicine and Translational Medicine, University of Tartu

VETAL Animal Health Products Company, BSL3+ Production Laboratuary /Turkey

Taiwan Centers for Disease Control

Takayuki Hishiki Kanagawa Prefectural Institute of Public Health

Takayuki Hishiki Kanagawa Prefectural Institute of Public Health, Department of Microbiology, Kanagawa, Japan

Tampa General Hospital Esoteric Research & Development Lab

Tanjungpura University Hospital

Technical Support Units for Scientific Research (UATRS), National Centre for Scientific and Technical Research (CNRST)

Technology Centre, Guangzhou Customs

Tejgaon College bmb lab

Texas Children's Microbiome Center

Texas Department of State Health Services

Texas Department of State Health Services - TXDSHS

Texas Department of State Health Services (TXDSHS)

Sonora Quest Laboratories

Thai National Influenza Center, Department of medical Science, Ministry of Public Health, Thailand

Thai Red Cross Emerging Infectious Diseases Center and Faculty of Medicine, Chulalongkorn University

Carrington Lab, Department of Preclinical Sciences, Faculty of Medical Sciences, The University of the West Indies

Carrington Lab, Department of Preclinical Sciences, Faculty of Medical Sciences, The University of the West Indies, St Augustine Campus

Carrington Lab, Department of PreClinical Sciences, Faculty of Medical Sciences, The University of the West Indies, St Augustine Campus

The Centre for Clinical Infection & Diagnostics Research, KCL

The Department of Infectious Disease Prevention and Control, Henan Provincial CDC, The Agricultural South Road Road, Zhengzhou, Henan 450016, China

the First Affiliated Hospital of Guangzhou Medical University & BGI-Shenzhen

The First Affiliated Hospital of Guangzhou Medical University & BGI-Shenzhen

The First People's Hospital of Yunnan Province, Medical Faculty of Kunming University of Science and Technology, Department of Clinical Laboratory, No. 157, Jinbi Road, Xishan District, Kunming, Yunnan 650032, China

The Foundation for Medical Research

The Hong Kong Polytechnic University Molecular Diagnostic Laboratory

The Hospital for Sick Children

Sdlitn 136/24 165 03, Prague Czech Republic

State Veterinary Institute Prague and The National Institute of Public Health

The National Institute of Public Health and State Veterinary Institute Prague

The National Institute of Public Health Center for Epidemiology and Microbiology

The National Laboratory of Health, Environment and Food, Maribor, Slovenia

OSU Polaris Molecular Laboratory

The Ohio State University-James Molecular Lab at Polaris

The Ohio State University James Molecular lab

The University of Hong Kong

Tilia Laboratories s.r.o.

Fondazione Policlinico Universitario Agostino Gemelli
Fondazione Policlinico Universitario Agostino Gemelli IRCCS
University Campus BioMedico of Rome
TN DOH Lab Services
CSIR-Institute of Genomics and Integrative Biology
Tokushima Prefectural Public Health, Pharmaceutical and Environmental Sciences Center
Tokyo Medical and Dental University
Tokyo Metropolitan Institute of Public Health
Tokyo Metropolitan Institute of Public Health, Department of Microbiology
Tokyo Metropolitan Institute of Public Health, Department of Microbiology; 3-24-1, Hyakunin-cho, Shinjuku-ku, Tokyo 169-0073, Japan
Tottori Prefectural Institute of Public Health and Environmental Science
Toyama Institute of Health
THSTI Bioassay laboratory
Tripler Army Medical Center
Dept. of Clinical Investigation
Department of Clinical Investigation
Tropical Infectious Diseases Research and Education Centre (TIDREC), Universiti Malaya, Kuala Lumpur, Malaysia
TSGH-CP molecular lab
TSGH-CP molecular lab
TSGH-CP molecular lab, Division of Clinical Pathology, Department of Pathology
Tulare County Public Health Laboratory
Tumor Immunology Unit, Department of Health Sciences, University of Palermo School of Medicine and National Research Council of Italy - High Performance Computing and Networking Institute (CNR-ICAR), Corso Tukory, 211, Palermo 90134, Italy
Gen Era Diagnostics Corporation Life Science Research and Molecular Diagnostics
TwinStrand Biosciences, Inc.
U.O. Microbiologia e Virologia, Azienda Provinciale per i Servizi Sanitari Provincia Autonoma di Trento, Ospedale S.Chiara
U.O. Microbiologia Laboratorio Unico Centro Servizi AUSL della Romagna
unknown
Hospital of Lithuanian University of Health Sciences (LSMU) Kaunas Clinics
UC Davis Genome Center
UCD National Virus Reference Laboratory
UCD National Virus Reference Laboratory
1. Tricity SARS-CoV-2 sequencing consortium: University of Gdansk, Medical University of Gdansk, Vaxican Ltd., Invicta Ltd. 2. National Institute of Public Health - National Institute of Hygiene, Warsaw, Poland
Kruglyak Lab
UCSC Genomics Institute
University of Warwick, for the COVID-19 Genomics (COG) UK Consortium
UHTL
UHTL, University Hospitals
Center for Microbiome Research
UMC Groningen, Clinical Virology, Department of Medical Microbiology and Infection Prevention
UMC Utrecht
UMGC
UMR190-Unit des virus emergents
UMR190-Unit des virus mergents
Project group Epidemiology of Highly Pathogenic Microorganisms, Robert Koch-Institute
Unit de Virologie du CHU d'Angers
Unit for Biological Agents, Department for CBRN Defence and Security, Swedish Defence Research Agency
Unit for Laboratory Development and Technology Transfer, Public Health Agency of Sweden
Unit di Analisi del Rischio ed Epidemiologia Genomica, Istituto Zooprofilattico Sperimentale dell'Emilia Romagna e della Lombardia (IZSLER)
Gencell Pharma
Universidad del Valle, Universidad Nacional de Colombia-Sede Palmira, International Center for Tropical Agriculture
Universidad del Valle
Universidad del Valle,TAO-Lab, VIREM, NEAS Network
International Centre for Genetic Engineering and Biotechnology (ICGEB) and ARGO Open Lab Platform
International Centre for Genetic Engineering and Biotechnology (ICGEB)and ARGO Open Lab Platform
Universidad Industrial de Santander
Universidad Industrial de Santander.
UNAL

Laboratrio de Bioinformtica e Biotecnologia (Labinftec/UFT)
Alea Genetiki Centar
University of Sarajevo, Veterinary Faculty
University of Sarajevo Veterinary Faculty
University Clinical Research Center, University of Sciences
African Centre of Excellence for Genomics of Infectious Diseases (ACEGID), Redeemer's University, Nigeria
University Hospital Basel, Clinical Virology
University Hospital Basel, Labormedizin
Widera/Toptan
University Hospital Limerick
University Hospital Ostrava
University Hospitals of Geneva Laboratory of Virology
University Hospitals of Geneva Laboratory of Virology
University Hospitals of Geneva, Laboratory of Virology
University Institute of Biochemistry and Biotechnology, Pir Mehr Ali Shah Arid Agriculture University Rawalpindi, Shamsabad, Murree Road, Rawalpindi, Punjab 46300, Pakistan
Evans Laboratory
Microbial Genomics Lab, Lebanese American University, Byblos
Istituto Zooprofilattico Sperimentale dell'Abruzzo e del Molise 'G. Caporale'.
Rissland/Pollock
Fusion Genomics Corporation
University of Hong Kong-Shenzhen Hospital
Roy J. Carver Biotechnology Center
University or Iowa, Lung Biology and Cystic Fibrosis Research Center, Pezzulo Lab
Walter Reed Army Institute of Research
University of Miami Immunology and Histocompatibility Laboratory
University of Montana Genomics Core (UMGC)
University of Oregon Genomics and Cell Charactericzation Core Facility (GC3F)
University of Oregon Genomics and Cell Characterization Core Facility (GC3F)
University of Rochester Medical Center (URMC) LABS
University of Rome Tor Vergata: Departm Experim Medicine Chair of Virology
University of South Carolina Functional Genomics Core
University of Texas at Austin Genome Sequencing and Analysis Facility (UTGSAF)
University of Texas, Genomic Sequencing and Analysis Facility
University of Tokyo Hospital
University of Ulsan College of Medicine and Asan Medical Center
University of Washington Medical Center, Seattle Flu Study
University of Wisconsin-Madison AIDS Vaccine Research Laboratories
University of Wisconsin Madison, AIDS Vaccine Research Laboratories
UNZAVET and PATH
University of Zambia, School of Veterinary Medicine, Disease Control
Center for Genomics and System Biology
Department of Virology, Public Health Laboratories Division, National Institute of Health
Laboratoire Sciences et Technologies de la Sant (STS) Institut Suprieur des Sciences de la Sant Universit Hassan 1er, Settat, Morocco
Microbiology Division
Public Health Virology Laboratory, Forensic and Scientific Services (PHV-FSS)
Research Center Of Tropical and Infectious Of Medical Sciences
Walk Lab Montana State University
Laboratorio di Genomica e Patologia molecolare
Dipartimento di Biotecnologie Mediche
Dipartimento di Biotecnologie Mediche, University of Siena
UOC Microbiology and Virology Policlinico Umberto I Rome, Italy
Microbial Genome Sequencing Center; Microbial Genomic Epidemiology Laboratory
Microbial Genome Sequencing Center; Microbial Genomic Epidemiology Laboratory, University of Pittsburgh
Microbial Genomic Epidemiology Laboratory, University of Pittsburgh
URMC LABS
Utah Public Health Laboratory, Utah Public Health Laboratory Infectious Disease submission group
UTGSAF
Utsunomiya City Institute of Public Health and Environment

UW Virology lab
UW Virology Lab
Laboratory of Medical Microbiology, University of Antwerp, Campus Drie Eiken, S6.26, Universiteitsplein 1, 2610, Wilrijk, Antwerp, Belgium
VA DCLS
Vanda Pharmaceuticals
Vermont Integrative Genomics Resource
Veterinary Specialized Institue Kraljevo
Scientific Veterinary Institute Novi Sad
Veterinary Specialized Institute Nis, Serbia
Veterinary Specialized Institute Sabac, Serbia
VIDRL and MDU-PHL
Victorian Infectious Diseases Reference Laboratory and Microbiological Diagnostic Unit Public Health Laboratory, Doherty Institute
Victorian Infectious Diseases Reference Laboratory and Microbiological Diagnostic Unit Public Health Laboratory, Doherty Institute
Vietnam Academy of Science of Technology, Institute of Biotechnology VietNam
ViFU
Vilnius University Hospital Santaros Klinikos
Vilnius university hospital Santaros Klinikos, Center of Laboratory Medicine
Institute of Biotechnology, Life Sciences Center, Vilnius University and Thermo Fisher Scientific
Department of Biosystems Science and Engineering, ETH Zurich
Hagmattstrasse 14, 4123 Allschwil, Switzerland
Viollier AG
ETH ZURICH D-BSSE, Mattenstrasse 26, Switzerland
Viral vaccines, VSVRI- Veterinary serum and vaccine research institute
Virginia DCLS
Virginia Division of Consolidated Laboratories
Virginia Division of Consolidated Laboratory Services
Virginia Division of Consolidated Laboratory Services (DCLS)
ViroGenetics - BSL3 Laboratory of Virology; Human Genome Variation Research Group & Genomics Centre MCB; Bioinformatics Research Group
ViroGenetics - BSL3 Laboratory of Virology; Human Genome Variation Research Group & Genomics Centre MCB; Bioinformatics Research Group Department of Virology
Virologa Molecular, CMBC, IVIC
Laboratorio Specialistico di Ematologia, Ospedale San Francesco, via Mannironi 1, 08100 Nuoro
Virologisches Institut, Universittsklinikum Erlangen
icddr, b, 68, Shaheed Tajuddin Ahmed Sarani, Dhaka, Dhaka 1212, Bangladesh
Virology and Legal Medicine Laboratories, Department of Biomedical Sciences and Public Health, University Politecnica delle Marche
The Francis Crick Institute
Virology Department, Royal Infirmary of Edinburgh, NHS Lothian
National Centre for Communicable Deseases (NCCD)
Dipartimento di Scienze Biomediche e Cliniche, L.Sacco, Universit di Milano
Dipartimento di Scenze Biomediche e Cliniche, L. Sacco, Universit di Milano
Dipartimento di Scienze Biomediche e Sanit Pubblica, Universit Politecnica delle Marche
Virology Lab,Department of Pathology, National Cheng Kung University Hospital
VIROLOGY LABORATORY-CHU NICE
Virology Laboratory National Institute for Infectious Diseases 'Lazzaro Spallanzani' IRCCS
Instituto Nacional de Sade Pblica (INSP)
Virology Laboratory, INMI L. Spallanzani
Virology Laboratory, Ospedali Riuniti, Ancona
Virology section, Okayama Prefectural Institute for Environmental Science and Public Health
Virology Unit
Virology Unit AOUP
Virology Unit, Agrobiodiversity and Biotechnology Project, CIAT - International Center for Tropical Agriculture, Km 17 Recta Cali-Palmira, Palmira, Valle del Cauca 76520000, Colombia
Fondazione Pisana per la Scienza ONLUS
Virolgy Unit, AOUP
Virology unit, AOUP
Virology Unit, AOUP
Virology Unit, AOUP, Pisa
Virology Unit, Institut Pasteur de Madagascar
Virology Unit, Institut Pasteur du Cambodge (Sequencing done by: Jessica E Manning/Jennifer A Bohl at Malaria and Vector Research Research Laboratory, National Institute of Allergy and Infectious Diseases and Vida Ahyong from Chan-Zuckerberg Biohub)

Virology, Iran University of Medical Sciences
Virology, Istituto Zooprofilattico Sperimentale dell'Abruzzo e del Molise 'G. Caporale'
Virology, University of Washington
Virology, ICAR-National Research Centre on Equines
Virology, Institute for Medical Virology, Paul Ehrlich St. 40, Frankfurt 60590, Germany
Virology, International Centre for Diarrhoeal Disease Research (icddr)
Razi Vaccine and Serum Research Institute, Co.
Virology, National Veterinary College of Toulouse, 23 Chemin des Capelles, Toulouse 31076, France
Epigenetics, Saarland University
Virus Ecology Section, RML
Virus Ecology, NIH
Virus Ecology, Rocky Mountain Laboratories, National Institutes of Health
National Institute of Virology-Microbial Containment Complex, Indian Council of Medical Research
Virus Research Laboratory, Department of Zoology, Osmania University, Tarnaka, Hyderabad, Telengana 500007, India
Virus Research Laboratory, Department of Zoology, Osmania University,Hyderabad,India
Institute of Biotechnology, DNA Sequencing and Genomics Laboratory, University of Helsinki
18. Tricity SARS-CoV-2 sequencing consortium: University of Gdansk, Medical University of Gdansk, Vaxican Ltd., Invicta Ltd. 2. National Institute of Public Health - National Institute of Hygiene, Warsaw, Poland
25. Tricity SARS-CoV-2 sequencing consortium: University of Gdansk, Medical University of Gdansk, Vaxican Ltd., Invicta Ltd. 2. National Institute of Public Health - National Institute of Hygiene, Warsaw, Poland
Voivodship Sanitary - Epidemiological Station in Warsaw
Dr. Suman Das Lab - Vanderbilt University Medical Center (VUMC) (https://my.vanderbilt.edu/daslab/)
WACCBIP, University of Ghana, Volta Road, Legon-Accra, Ghana
KUWAIT CANCER CONTROL CENTER
Washington State Department of Health Public Health Laboratories
Washington University in St. Louis
Weifang CDC
West African Centre for Cell Biology of Infectious Pathogens (WACCBIP), University of Ghana, Volta Road, Legon-Accra, Ghana
The Ohio State University College of Medicine
African Centre of Excellence for Genomics of Infectious Diseases (ACEGID), Redeemer's University, Ede
Wisconsin State Laboratory of Hygiene Communicable Disease Division
Wisplinghoff Laboratories
Wits VIDA
Wojewdzka Stacja Sanitarno-Epidemiologiczna w Olsztynie, Laboratorium Bada Epidemiologiczno-Klinicznych
Wojewodzka Stacja Sanitarno-Epidemiologiczna w Olsztynie, Laboratorium Badan Epidemiologiczno-Klinicznych
Harris/Rasmussen Lab
Worobey Lab on behalf of the Arizona COVID-19 Genomics Union
Worobey Lab, Department of Ecology and Evolutionary Biology, University of Arizona
COVID-HUB-PL, Institute of Bioorganic Chemistry PAS, Z Noskowskiego 12/14, 61-704 Poznan, Poland
Beijing Genomics Institute (BGI)
Wuhan Institute of Virology, Chinese Academy of Sciences
State Key Laboratory of Agriculture Microbiology, Huazhong Agric Laboratory of Animal Virology, College of Veterinary Medicine
Wyoming Public Health Laborator
Tehran University of Medical Sciences
Yamagata Prefectural Institute of Public Health
Yamaguchi Prefectural Institute of Public Health and Environment
Yunnan Center for Disease Control Prevention, Institute for Acute Communicable Disease Prevention and Control
Biotechnology laboratory, Center for advanced technology
Zoontic and Exotic infection Diseases Division
Zoonotic and Exotic infection Diseases Division, Harbin Veterinary Research Institute, CAAS
Zurita & Zurita Laboratorios
Istituto Zooprofilattico Sperimentale dellAbruzzo e Molise G. CaporaleMEDICO COMPETENTE P.O. L'AQUILA
